# The *Xanthomonas euvesicatoria* type III effector XopAU is an active protein kinase that manipulates plant MAP kinase signaling

**DOI:** 10.1371/journal.ppat.1006880

**Published:** 2018-01-29

**Authors:** Doron Teper, Anil Madhusoodana Girija, Eran Bosis, Georgy Popov, Alon Savidor, Guido Sessa

**Affiliations:** 1 School of Plant Sciences and Food Security, Tel Aviv University, Tel Aviv, Israel; 2 Department of Biotechnology Engineering, ORT Braude College, Karmiel, Israel; 3 The Nancy & Stephen Grand Israel National Center for Personalized Medicine, Weizmann Institute of Science, Rehovot, Israel; University of Toronto, CANADA

## Abstract

The Gram-negative bacterium *Xanthomonas euvesicatoria* (*Xe*) is the causal agent of bacterial spot disease of pepper and tomato. *Xe* delivers effector proteins into host cells through the type III secretion system to promote disease. Here, we show that the *Xe* effector XopAU, which is conserved in numerous *Xanthomonas* species, is a catalytically active protein kinase and contributes to the development of disease symptoms in pepper plants. *Agrobacterium*-mediated expression of XopAU in host and non-host plants activated typical defense responses, including MAP kinase phosphorylation, accumulation of pathogenesis-related (PR) proteins and elicitation of cell death, that were dependent on the kinase activity of the effector. XopAU-mediated cell death was not dependent on early signaling components of effector-triggered immunity and was also observed when the effector was delivered into pepper leaves by *Xanthomonas campestris* pv. *campestris*, but not by *Xe*. Protein-protein interaction studies in yeast and *in planta* revealed that XopAU physically interacts with components of plant immunity-associated MAP kinase cascades. Remarkably, XopAU directly phosphorylated MKK2 *in vitro* and enhanced its phosphorylation at multiple sites *in planta*. Consistent with the notion that MKK2 is a target of XopAU, silencing of the MKK2 homolog or overexpression of the catalytically inactive mutant MKK2_K99R_ in *N*. *benthamiana* plants reduced XopAU-mediated cell death and MAPK phosphorylation. Furthermore, yeast co-expressing XopAU and MKK2 displayed reduced growth and this phenotype was dependent on the kinase activity of both proteins. Together, our results support the conclusion that XopAU contributes to *Xe* disease symptoms in pepper plants and manipulates host MAPK signaling through phosphorylation and activation of MKK2.

## Author summary

Many bacterial pathogens inject effector proteins into their eukaryotic host cells through the type III secretion system to modulate host cellular processes. Elucidating the function of bacterial effectors and identification of their host targets is important for understanding the molecular mechanisms of pathogenicity and host immunity. In this study, we analyzed the mode of action of XopAU, a type III effector from the pepper and tomato pathogen *Xanthomonas euvesicatoria*. We found that XopAU is a catalytically active protein kinase providing the first report of an effector from a plant bacterial pathogen that displays such an enzymatic activity. We show that expression of XopAU activates immune responses and contributes to the development of disease symptoms. Interestingly, XopAU-mediated phenotypes are altered when the effector is expressed by different species of *Xanthomonas*, suggesting an interplay between this effector and other species-specific virulence determinants. Furthermore, we provide biochemical and genetic evidence that XopAU interferes with host immune signaling by activating the MAPKK MKK2. Together, our results provide new insights into the interaction between the plant immune system and bacterial type III effector proteins.

## Introduction

Plant immunity against microbial pathogens relies on a complex detection and signaling network [1]. A first line of plant immune responses is activated by cell surface-exposed pattern recognition receptors (PRRs) that detect broadly conserved pathogen molecules (pathogen/microbe-associated molecular patterns, PAMP/MAMPs) [2]. Activation of PRRs initiates downstream signaling events that lead to the production of reactive oxygen species, stimulation of mitogen-activated protein kinase (MAPK) cascades, defense gene induction, release of ethylene, and callose deposition at the plant cell wall [3,4]. These host responses limit the growth of a large number of potential pathogens and are referred to as pattern-triggered immunity (PTI). Host-adapted pathogens overcome PTI through the activity of effector proteins that are targeted to the plant apoplast or delivered into the host cytoplasm [5]. To cope with these pathogens, plants have evolved other types of receptors known as resistance (R) proteins that specifically recognize effectors or their activity [6]. R proteins activate effector-triggered immunity (ETI) that consists of defense responses similar to PTI, but more robust and often accompanied by a localized cell death known as the hypersensitive response (HR) [7].

Mitogen-activated protein kinases (MAPKs) cascades play a fundamental role in plant immunity and are involved in both PTI and ETI signaling [8]. The tomato MAPKKK MAP3Kα and MAP3Kε were found to participate in signaling pathways that mediate elicitation of the ETI-associated HR in *N*. *benthamiana* plants, and to be required for disease resistance to bacterial pathogens in tomato [9,10]. The MEK2 MAPKK was identified as a central regulator of the HR elicited upon detection of effectors by several R proteins in *N*. *benthamiana* plants, and as required for tomato disease resistance to *Pseudomonas* and *Xanthomonas* bacteria [11]. Epistasis analysis revealed that MEK2 acts downstream of both MAP3Kα and MAP3Kε and upstream of the SIPK and WIPK MAP kinases [9,10,12]. Notably, SIPK and WIPK, and their respective Arabidopsis homologs MPK6 and MPK3, are also important regulators of PTI [13,14]. In line with these findings, the Arabidopsis MKK4 and MKK5, which are the MAPKKs upstream of MPK6 and MPK3, were also shown to participate in PTI signaling [13].

Many Gram-negative plant pathogenic bacteria utilize a type III secretion system to deliver effector proteins into the host cells [15]. Type III effector proteins contribute to bacterial virulence by subverting plant signaling pathways, suppressing immune responses, and modulating host metabolism and hormone signaling [16,17]. MAPK cascades have emerged as important targets of type III effectors of plant and mammalian bacterial pathogens [18]. For example, *Yersinia pestis* YopJ interferes with the activation of immune responses in mammalian cells by inhibiting phosphorylation of MAPKK6 through acetylation of Ser and Thr residues in the activation loop of the kinase [19]. The *Salmonella* phosphothreonine lyase effector SpvC irreversibly removes a phosphate from ERK1/2 MAPK to downregulate cytokine release from infected cells [20]. Several *Pseudomonas syringae* effectors were found to suppress immunity in Arabidopsis by interfering with the activity of components of MAPK cascades: HopAI1 encodes a phosphothreonine lyase that irreversibly removes a phosphate from MPK3 and MPK6 thereby suppressing PTI activation [21]. HopF2 inhibits PTI through inactivation of MKK5, the upstream MAPKK of MPK3 and MPK6, by ADP-ribosylation [22]. Finally, AvrB enhances plant susceptibility by promoting phosphorylation and activation of MPK4, which perturbs hormone signaling to the benefit of the bacterium [23].

The Gram-negative bacterium *Xanthomonas euvesicatoria* (*Xe*) is the causal agent of bacterial spot disease in pepper and tomato plants [24]. *Xe* bacteria penetrate into plant tissues through wounds and stomata, proliferate and colonize the apoplast of the aerial parts of the plants, and cause the appearance of water soaked lesions that develop into necrotic black spots. The ability of *Xe* to cause disease largely depends on the type III secretion system. To date, the pool of known *Xe* effectors includes approximately 35 proteins mostly identified in the 85–10 strain [25–27]. Biochemical activity and cellular targets have been elucidated only for a few *Xe* effectors. The XopD effector is a SUMO protease that alters host transcription to suppress hormone signaling [28]. The XopN and XopQ effectors target host 14-3-3 proteins to suppress PTI and ETI signaling, respectively [29,30]. The XopJ effector causes degradation of a proteasome subunit to suppress salicylic acid-mediated defense and protein secretion [31–33].

By a machine learning approach applied to the *Xe* strain 85–10, we have recently identified XopAU as a type III secreted effector and demonstrated its translocation into cells of pepper leaves [26]. XopAU is conserved in multiple *Xanthomonas* spp. and in a few *Acidovorax* spp., and encodes a putative serine/threonine protein kinase [26]. The *xopAU* gene from *Xe* 85–10 contains a plant-inducible promoter (PIP) box and expression of its homolog from *Xanthomonas citri* was found to be regulated by the HrpG/HrpX-regulon, which controls transcription of genes encoding structural components of the type III secretion system and some effector genes [34]. Here, we investigated XopAU molecular properties and virulence function. We found that XopAU is a catalytically active protein kinase that contributes to the development of disease symptoms in susceptible plants. In addition, we identified the MAPKK MKK2 as a binding partner and direct substrate of XopAU phosphorylation. Moreover, by genetic and functional analysis we provide evidence that MKK2 is required for the XopAU molecular function.

## Results

### The *Xanthomonas* type III effector XopAU encodes a catalytically active protein kinase

**Fig 1:**
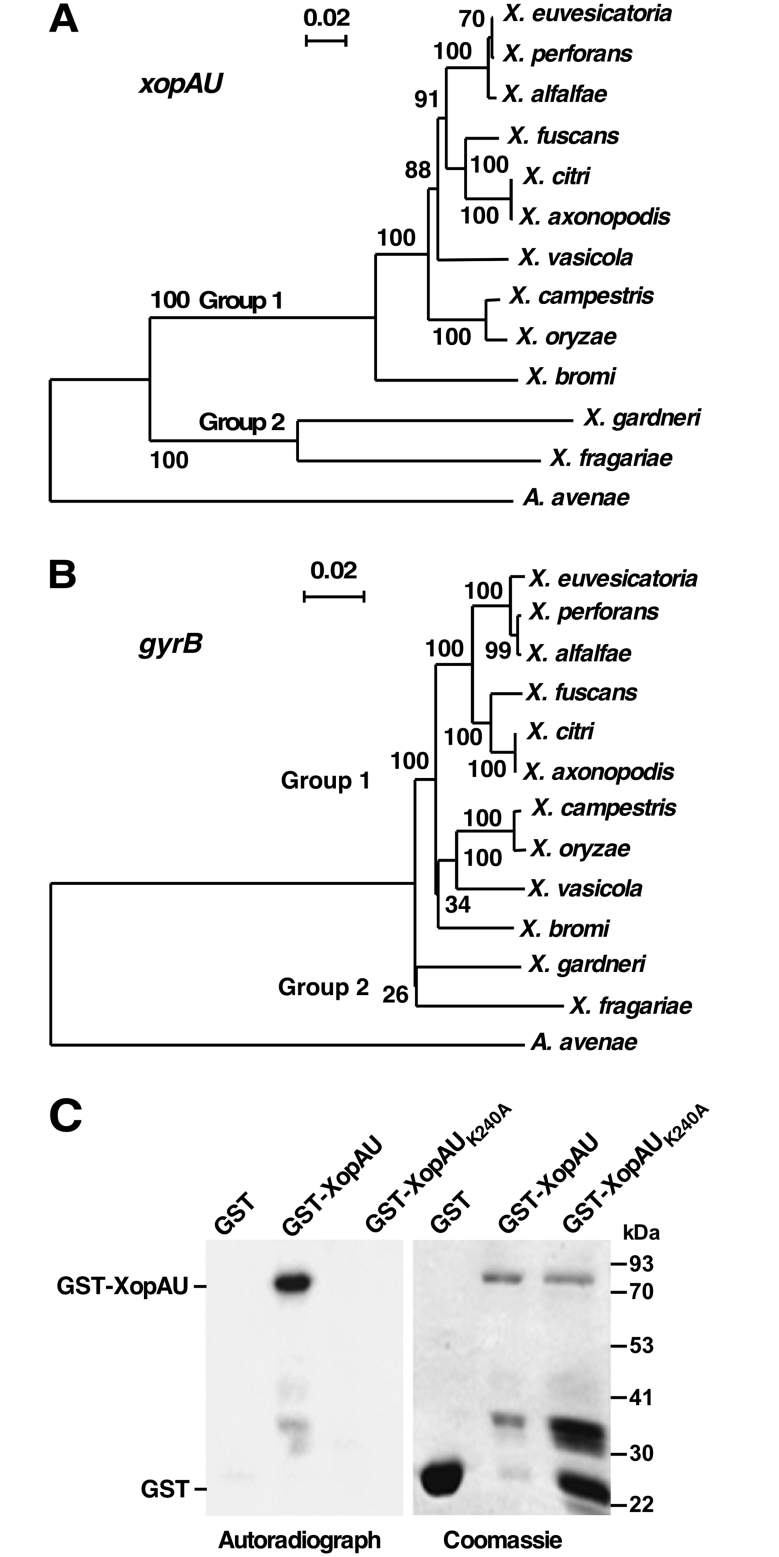
The *xopAU* gene of *Xe* strain 85–10 is conserved in multiple *Xanthomonas* species and encodes a protein kinase. Phylogenetic tree of *xopAU* (A) and *gyrB* (B) homologs generated with Clustal X [68] using the standard neighbor joining phylogenetic tree definitions. NCBI *xopAU* and *gyrB* accession numbers and a sequence alignment of the *xopAU* genes included in the tree are reported in S1 Table and S1 Fig, respectively. Bootstrap values of 100 replications are shown on nodes. (C) *In vitro* kinase assay performed by incubating the indicated proteins in the presence of [γ-^32^P]ATP. Proteins were separated by SDS-PAGE and detected by autoradiography or Coomassie staining.

XopAU is a type III secreted effector originally identified in the *Xanthomonas euvesicatoria* strain 85–10 (*Xe*) [26]. Homologs of the effector are present in multiple species of the *Xanthomonas* genus and in *Acidovorax* spp. Promoter regions of all *xopAU* homologs contain a PIP box motif (S1 Table) indicating that their expression is controlled by the HrpG/HrpX regulon [35]. To determine evolutionary relationships between *xopAU* homologous genes, a representative of each *Xanthomonas* species encoding *xopAU* and an *Acidovorax avenae* homolog were used to construct a phylogenetic tree (Fig 1A, S1 Fig and S1 Table). *xopAU* homologs classified into two allelic groups and their phylogenetic relationships correlated to the relationships among the corresponding *Xanthomonas* species that were deduced by a sequence comparison of the *gyrB* phylogenetic marker gene [36] (Fig 1 and S1 Table). This correlation suggests that the two alleles were transmitted vertically after their acquisition in parental strains. *xopAU* homologs of group 1 share a low degree of sequence similarity to group 2 homologs, have a different GC content (group 1, 62.9%-64.3%; group 2, 55.2%-55.8%), and a distinct genomic location (S1 Table; S2 Fig) implying that the two *xopAU* alleles were independently acquired by *Xanthomonas* spp. The *Xanthomonas* species containing the group 1 *xopAU* allele correspond to a complete clade in the *Xanthomonas* genus (Fig 1A) [37]. Conversely, *Xanthomonas* species containing the group 2 *xopAU* allele (*X*. *fragariae* and *X*. *gardneri*) are members of a clade that also includes the *X*. *arboricola* species [37], which does not encode a *xopAU* allele. The borders of the *xopAU* deletion in the genome of a *X*. *arboricola* strain are shown in S2B Fig.

Analysis of the NCBI conserved domains database revealed that *Xe* XopAU contains a putative protein kinase domain at the C-terminus (203–493 amino acids), which includes kinase subdomains I-XI and the majority of the residues that are nearly invariant throughout the kinase superfamily [38] (S3 Fig). Notably, the kinase nearly invariant residues identified in *Xe* XopAU are also conserved in its homologs from other *Xanthomonas* strains. To test whether XopAU is a catalytically active protein kinase, it was expressed in *E*. *coli* as a glutathione S-transferase (GST) fusion, purified and assayed for kinase activity in the presence of [γ-^32^P]ATP. The GST-XopAU fusion was able to autophosphorylate *in vitro* and its activity was abolished by the introduction of an alanine substitution at the conserved lysine (K240) of the putative ATP binding site (Fig 1C). Together, this analysis revealed that the *xopAU* gene is conserved in the genome of numerous *Xanthomonas* species and XopAU of the *Xe* strain 85–10 is a catalytically active protein kinase.

### *Agrobacterium*-mediated expression of XopAU *in planta* promotes activation of immune responses

**Fig 2:**
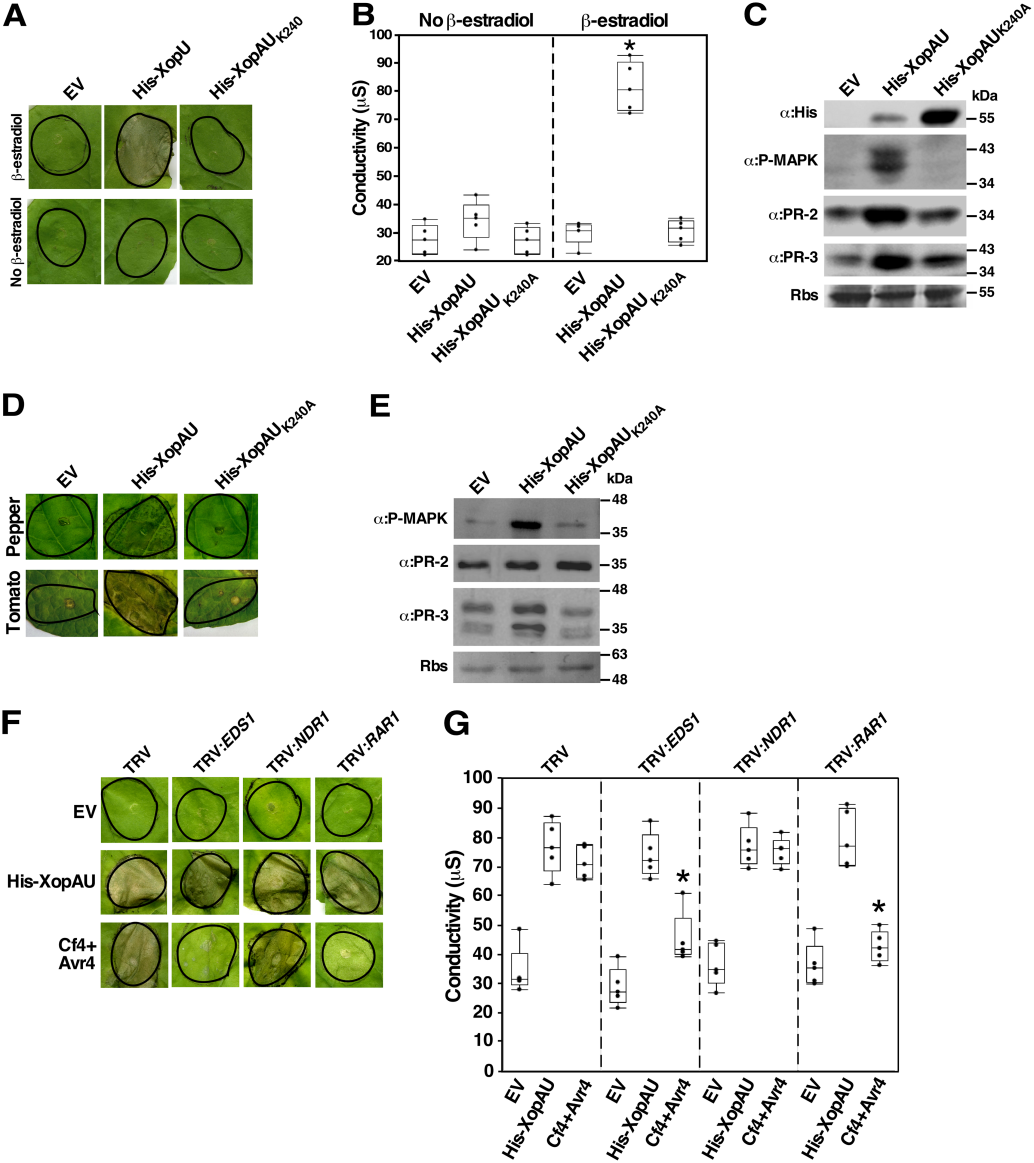
*Agrobacterium*-mediated expression of XopAU induces plant immune responses. (A-E) *N*. *benthamiana* leaves were infiltrated with *Agrobacterium* strains for the expression of His-XopAU or His-XopAU_K240A_ driven by an estradiol-inducible system, or carrying an empty vector (EV), and treated with 17β-estradiol 24 h later. (A) Photographs of inoculated areas at 48 h after 17β-estradiol application. (B) Electrolyte leakage at 24 h with or without 17β-estradiol application. The box plot displays 25^th^, 50^th^ (middle line) and 75^th^ percentiles (*n* = 5). An asterisk indicates a significant difference (Mann-Whitney U test, *p* value <0.05) compared to EV. (C) Total protein was extracted from *N*. *benthamiana* leaves 8 h after 17β-estradiol application and immunoblotted with the indicated antibodies. Rbs, Rubisco loading control stained by Ponceau S. (D) Leaves of the pepper line ECW30R or the tomato line Hawaii 7981 were infiltrated with *Agrobacterium* strains as in (A) and photographed at 96 h after 17β-estradiol application. (E) Total protein was extracted from pepper leaves at 8 h after 17β-estradiol application and immunoblotted with the indicated antibodies. (F and G) *N*. *benthamiana* plants were infected with TRV, TRV:*EDS1*, TRV:*NDR1* and TRV:*RAR1*. Leaves of silenced plants were inoculated with *Agrobacterium* (OD_600_ = 0.02) carrying an empty vector (EV), a vector for expression of His-XopAU from an estradiol-inducible system or Cf4/Avr4 driven by the CaMV 35S promoter. Inoculated leaves were treated with 17β-estradiol 24 h later. (F) Photographs of inoculated areas at 36 h after 17β-estradiol application. (G) Electrolyte leakage at 24 h after 17β-estradiol application. The box plot displays 25^th^, 50^th^ (middle line) and 75^th^ percentiles (*n* = 5). An asterisk indicates a significant difference (Mann-Whitney U test, *p* value <0.05) compared to EV. (A-G) Experiments were repeated at least three times with similar results.

To examine whether XopAU from *Xe* causes detectable phenotypes *in planta*, the effector was fused to a His tag (His-XopAU) and transiently expressed via *Agrobacterium* under the control of an estradiol inducible system in leaves of the *Xe* non-host plant *N*. *benthamiana*. At 24–48 hours after estradiol application, cell death was visible in leaf areas expressing His-XopAU and confirmed by a higher ion leakage than in leaf areas infiltrated with *Agrobacterium* carrying an empty vector (Fig 2A and 2B). No cell death or ion leakage was observed without estradiol application. Furthermore, the His-XopAU_K240A_ kinase deficient variant did not induce a visible cell death and ion leakage (Fig 2A and 2B) indicating that XopAU kinase activity was required for this phenotype. Expression of wild-type and kinase deficient His-XopAU variants in the infiltrated areas after estradiol application was confirmed by Western blot analysis (Fig 2C).

Because cell death is a typical immune response triggered by pathogens in resistant plants, we hypothesized that XopAU activates immune signaling. To test this hypothesis, we transiently expressed His-XopAU in *N*. *benthamiana* leaves and monitored phosphorylation of MAP kinases and accumulation of pathogenesis-related (PR) proteins, which are additional phenotypes associated with plant immunity [8,39]. Phosphorylation of MAP kinases was assessed by using antibodies against the phosphorylated form of mammalian MAP kinases of the ERK family that recognize also phosphorylated plant MAP kinases. Accumulation of PR proteins was monitored with antibodies against the tobacco PR-2 and PR-3 isoforms. As shown in Fig 2C, MAP kinase phosphorylation and PR protein accumulation were induced in leaves of *N*. *benthamiana* plants expressing His-XopAU, but no induction was observed in leaves expressing the kinase deficient variant His-XopAU_K240A_.

To examine whether XopAU induces immune responses in *Xe* host plants, wild-type and kinase deficient His-XopAU variants were transiently expressed via *Agrobacterium* in leaves of the pepper line ECW30R and tomato line Hawaii 7981. Similar as in *N*. *benthamiana* leaves, expression of wild-type but not kinase deficient His-XopAU in these plants induced a cell death at 48–72 hours after estradiol application (Fig 2D). In pepper leaves, cell death was accompanied by enhanced MAP kinase phosphorylation and higher accumulation of the PR-3 protein, but not of PR-2 (Fig 2E). Together, these results suggest that expression of XopAU via *Agrobacterium* activated immune signaling in *Xe* host and non-host plants.

To assess whether activation of immune signaling by XopAU is caused by recognition of the effector by an R protein, we tested if silencing of early components of ETI signaling affects XopAU-mediated cell death. The genes silenced in these experiments were *EDS1* and *NDR1*, which are required for ETI mediated by multiple *R* genes of the TIR-NBS-LRR and CC-NBS-LRR class, respectively, and *RAR1*, which is required for ETI mediated by multiple *R* genes of different structural classes [40]. Virus-induced gene silencing (VIGS) techniques based on the tobacco rattle virus (TRV) vector were used to silence the genes in *N*. *benthamiana* plants [41]. Four weeks after infection of plants with the TRV vector carrying fragments of the genes to be silenced, transcript levels of *NDR1*, *EDS1* and *RAR1* were reduced by about 60% to 80% in silenced plants as compared to plants infected with the TRV empty vector (S4 Fig). At this time, *Agrobacterium* strains expressing His-XopAU were used to inoculate silenced and control plants, and cell death was monitored visually and quantified by measuring ion leakage. As a control, silenced leaves were also inoculated with *Agrobacterium* expressing the *R* gene/effector gene pair *Cf4*/*avr4*, which elicits a hypersensitive response in *N*. *benthamiana* leaves that is dependent on expression of *EDS1* and *RAR1*, but not of *NDR1* [42]. As expected, silencing of *EDS1* and *RAR1*, but not of *NDR1* severely reduced *Cf4*/*avr4*-mediated cell death and ion leakage (Fig 2F and 2G). Conversely, cell death mediated by His-XopAU was not affected by silencing of any of the tested ETI signaling components (Fig 2F and 2G). These results suggest that it is unlikely that the cell death observed upon XopAU expression in leaf tissues is triggered by recognition of the effector by an R protein.

### XopAU promotes chlorosis and accumulation of PR proteins when delivered by *Xe* in pepper leaves

**Fig 3:**
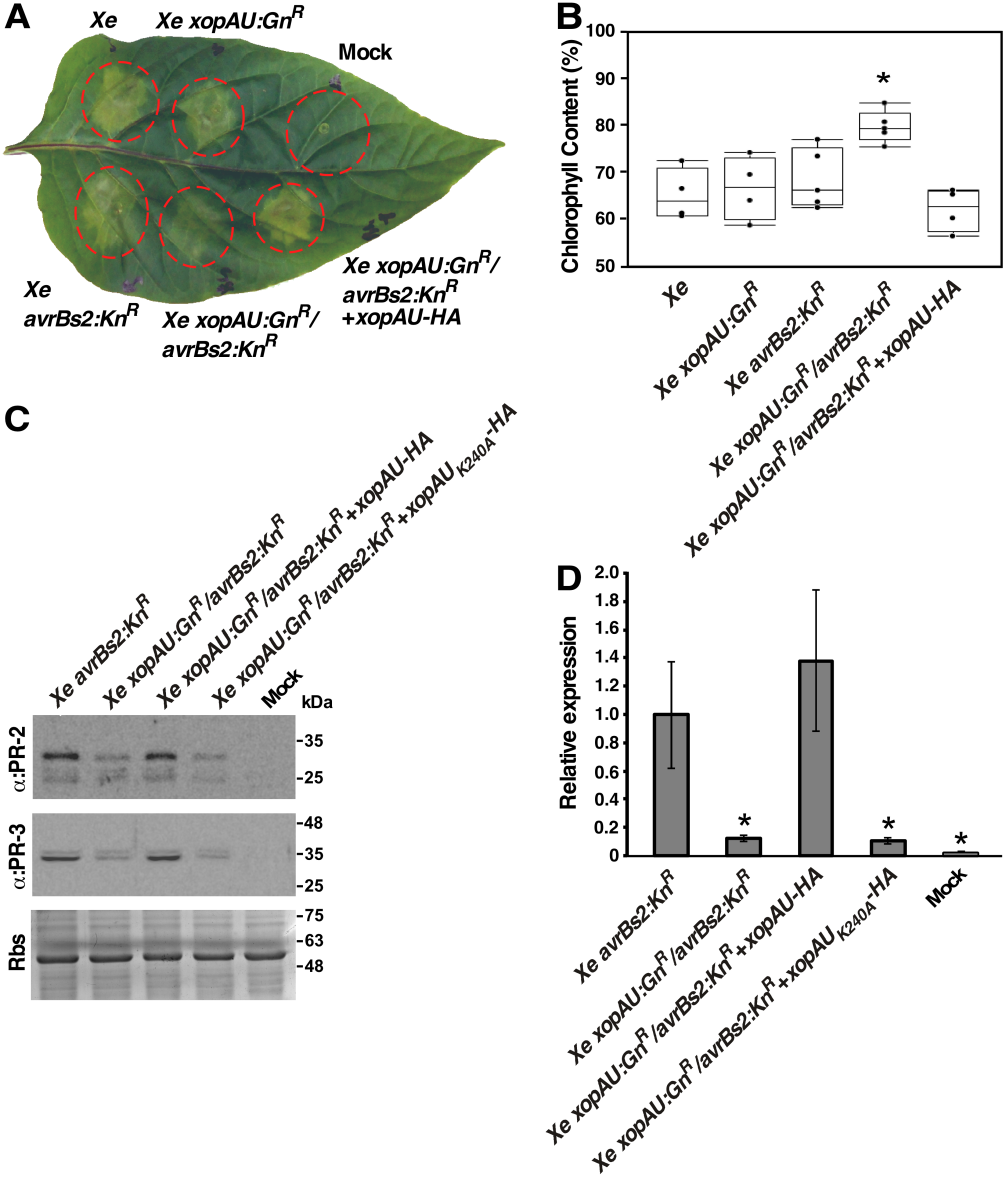
Phenotypic analysis of *xopAU* gene inactivation. Leaves of the pepper line ECW30R were syringe-infiltrated with a 10 mM MgCl_2_ mock solution or with suspensions (1 x 10^7^ CFU/ml) of the indicated strains. (A) Photograph of an inoculated leaf at 5 days post-inoculation (dpi). (B) Chlorophyll content relative to mock-inoculated areas at 5 dpi. The box plot displays 25^th^, 50^th^ (middle line) and 75^th^ percentiles (*n* = 4 or 5 biological repeats). An asterisk indicates a significant difference (Mann-Whitney U test, *p* value <0.05) relative to *Xe avrBs2*:*Kn*^*R*^. (C) Total protein was extracted from the infected leaves at 3 days post -inoculation (dpi) and immunoblotted with the indicated antibodies. Rbs, Rubisco loading control stained by Ponceau S. (D) mRNA abundance of the *PR-1* gene in the inoculated areas was measured by qRT-PCR at 72 h post-inoculation and calculated relative to areas inoculated with the *Xe avrBs2*:*Kn*^*R*^ strain. Values are means ± SD of three biological repeats. Asterisks indicate a significant difference (Student’s *t* test, *p* value < 0.05) relative to *Xe avrBs2*:*Kn*^*R*^.

To assess the contribution of XopAU to bacterial virulence, the corresponding gene was inactivated in *Xe* bacteria by insertion mutagenesis. The mutant strain *Xe xopAU*:Gn^R^ and wild-type *Xe* were used to infect ECW30R pepper plants that were then monitored for bacterial growth and development of disease symptoms. Disease symptoms were estimated visually and quantified by measuring chlorophyll content and ion leakage, as parameters of chlorosis and necrosis that are typically observed in pepper leaves infected by *Xe* bacteria. Leaves infected with the mutant strain *Xe xopAU*:Gn^R^ displayed a similar chlorophyll content, ion leakage and bacterial growth as leaves infected with wild-type *Xe* bacteria (Fig 3 and S5A Fig).

We hypothesized that a weak contribution of XopAU to *Xe* pathogenicity is more likely to be revealed in an attenuated bacterial strain. To test this possibility, we generated the double mutant strain *Xe xopAU*:Gn^R^/*avrBs2*:*Kn*^*R*^, mutated in both the *xopAU* and *avrBs2* effector genes. The AvrBs2 effector was chosen for this analysis because it was previously shown to contribute to *Xe* virulence activity and its deletion allowed to reveal the virulence activity of other effectors [43,44]. The double mutant along with wild-type *Xe* and the single mutants *Xe xopAU*:*Gn*^*R*^ and *Xe avrBs2*:*Kn*^*R*^ were used to infect ECW30R pepper plants that were then monitored for bacterial growth, chlorophyll content and ion leakage. In infected leaves, the *Xe avrBs2*:*Kn*^*R*^ mutant displayed reduced bacterial growth and ion leakage, but similar chlorophyll content, as compared to *Xe* wild-type and *Xe xopAU*:*Gn*^*R*^ (Fig 3 and S5A Fig). The *Xe xopAU*:*Gn*^*R*^*/avrBs2*:*Kn*^*R*^ double mutant was similar to *Xe avrBs2*:*Gn*^*R*^ in bacterial growth and ion leakage, but caused less chlorosis as indicated by a higher chlorophyll content (Fig 3 and S5 Fig). To confirm that the reduction in chlorotic symptoms was the result of a mutation in *xopAU*, the gene was re-introduced into the *Xe xopAU*:*Gn*^*R*^*/avrBs2*:*Kn*^*R*^ strain driven by its native promoter. When inoculated into pepper leaves the complemented *Xe xopAU*:*Gn*^*R*^*/avrBs2*:*Kn*^*R*^(*xopAU*) strain caused similar disease symptoms as the *Xe avrBs2*:*Kn*^*R*^ strain (Fig 3 and S5A Fig).

Because expression of His-XopAU via *Agrobacterium* activated immune signaling (see above), we tested whether deletion of the *xopAU* gene negatively affects the activation of defense responses. We monitored accumulation of the PR proteins PR-2 and PR-3 and mRNA levels of the *PR-1* gene in infected pepper leaves at 3 dpi. Western blot analysis revealed that PR-2 and PR-3 accumulation was lower in leaves inoculated with the *Xe xopAU*:*Gn*^*R*^*/avrBs2*:*Kn*^*R*^ double mutant strain than in leaves inoculated with the *Xe avrBs2*:*Kn*^*R*^ strain (Fig 3C). Leaves inoculated with the *Xe xopAU*:*Gn*^*R*^*/avrBs2*:*Kn*^*R*^ double mutant complemented with XopAU-HA driven by its native promoter, but not with the kinase deficient XopAU-HA_K240A_, accumulated similar PR-2 and PR-3 protein levels as leaves infected with the *Xe avrBs2*:*Kn*^*R*^ strain. Similarly, qRT-PCR analysis revealed that transcript levels of the *PR-1* gene were about 8.6-fold lower in leaves inoculated with the *Xe xopAU*:*Gn*^*R*^*/avrBs2*:*Kn*^*R*^ double mutant or with this strain complemented with the kinase deficient XopAU-HA_K240A_ than in leaves infected with the *Xe avrBs2*:*Kn*^*R*^ strain or with the double mutant strain complemented with XopAU-HA (Fig 3D)_._ These results indicate that expression of a catalytically active XopAU in *Xe* strains promotes the activation of defense responses in infected pepper leaves.

**Fig 4:**
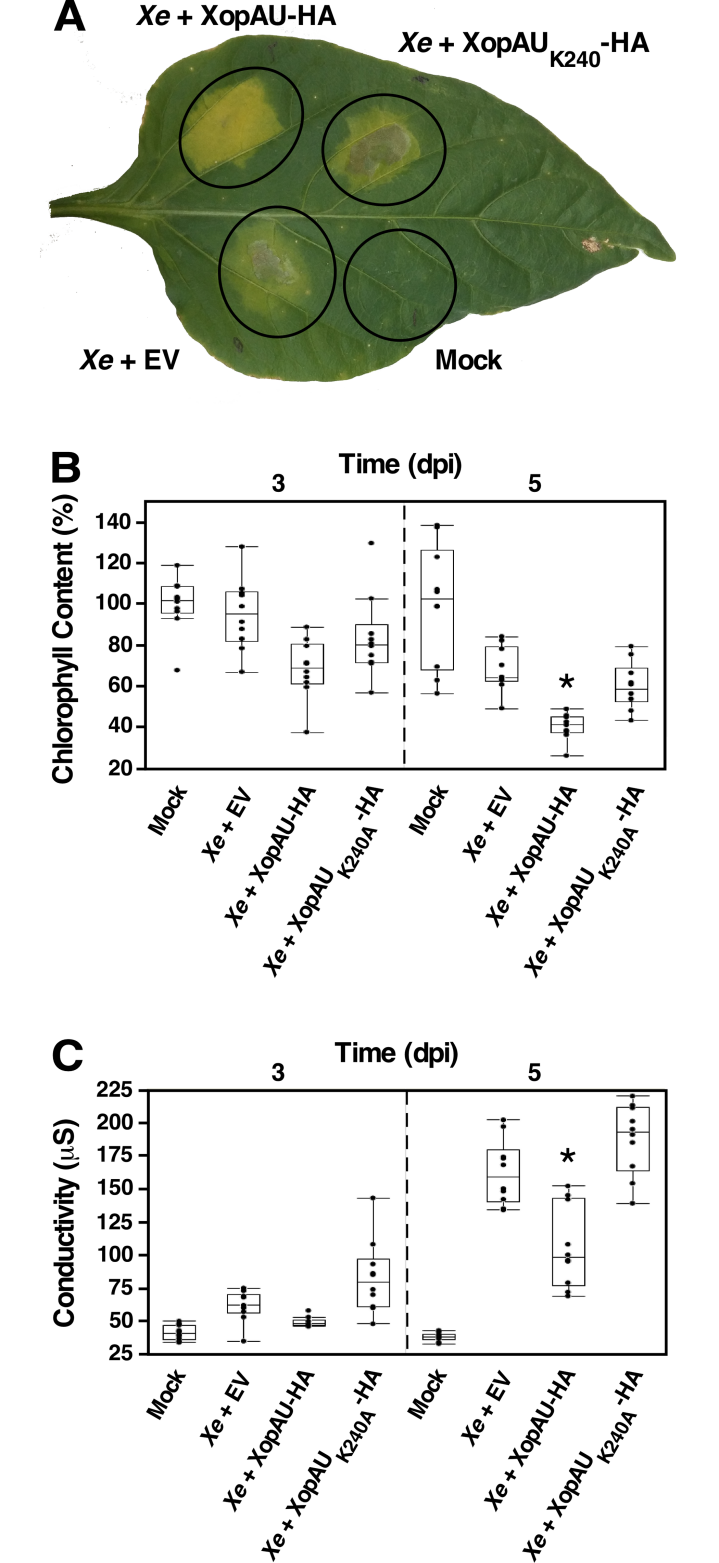
Phenotypic analysis of *Xe* strains overexpressing XopAU. Leaves of the pepper line ECW30R were syringe-infiltrated with a 10 mM MgCl_2_ mock solution or with suspensions (1 x 10^7^ CFU/ml) of *Xe* strains carrying a vector either empty (EV) or for expression of XopAU-HA or XopAU_K240A_-HA. (A) Photograph of an inoculated leaf at 5 days post-inoculation (dpi). (B) Chlorophyll content relative to mock-inoculated areas at 3 and 5 dpi. (C) Electrolyte leakage in the inoculated areas at 3 and 5 dpi. In B and C, box plots display 25^th^, 50^th^ (middle line) and 75^th^ percentiles (*n* = 10). Asterisks indicate a significant difference (Mann-Whitney U test, *p* value <0.05) relative to *Xe* containing an empty vector. Experiments were repeated at least three times with similar results.

To further assess the contribution of XopAU to development of disease symptoms caused by *Xe*, we engineered a *Xe* strain carrying a HA-tagged XopAU variant (XopAU-HA) driven by a constitutive *lac* promoter in a broad-host plasmid. Overexpression of XopAU-HA in this strain was validated by Western blot analysis (S6A Fig). A mock solution, *Xe* bacteria overexpressing XopAU-HA or carrying an empty vector were infiltrated into ECW30R pepper leaves that were then monitored for the development of chlorosis and necrosis (*i*.*e*. chlorophyll content and ion leakage, respectively) at 1, 3 and 5 days post-inoculation (dpi). *Xe* overexpressing XopAU-HA displayed lower chlorophyll content and reduced ion leakage, compared to *Xe* containing an empty vector (Fig 4). To confirm that the observed phenotype is due to the biochemical activity of XopAU, we generated an *Xe* strain overexpressing the catalytically inactive XopAU_K240A_-HA variant. No difference was observed in chlorosis and ion leakage between pepper plants inoculated with *Xe* overexpressing XopAU_K240A_-HA and *Xe* carrying an empty vector (Fig 4). Together, observations obtained by using *Xe xopAU*:*Gn*^*R*^*/avrBs2*:*Kn*^*R*^ double mutant and *Xe* bacteria overexpressing XopAU-HA suggest that the XopAU effector participates in the development of disease symptoms.

**Fig 5:**
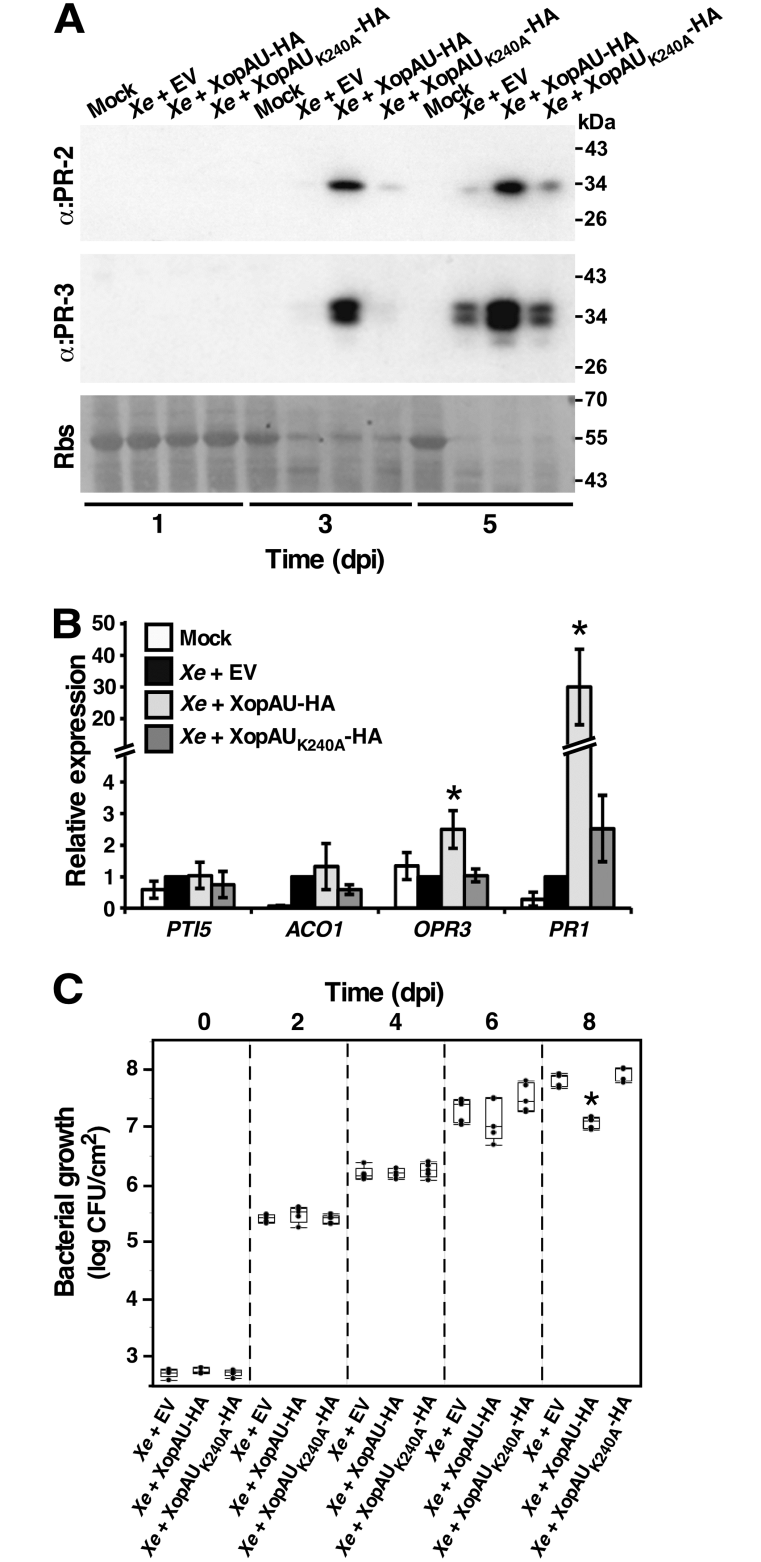
Expression of defense and stress marker genes in leaves infected with *Xe* overexpressing XopAU. Leaves of the pepper line ECW30R were syringe-infiltrated with a 10 mM MgCl_2_ mock solution (Mock) or with suspensions (1 x 10^7^ CFU/ml) of *Xe* strains carrying a vector either empty (EV) or for expression of XopAU-HA or XopAU_K240A_-HA. (A) Total protein was extracted from the infected leaves at the indicated days post-inoculation (dpi) and immunoblotted with the indicated antibodies. Rbs, Rubisco loading control stained by Ponceau S. (B) mRNA abundance of the indicated genes in the inoculated areas was measured by qRT-PCR at 16 h post-inoculation and calculated relative to areas inoculated with the *Xe* strain carrying an empty vector. Values are means ± SE of three biological repeats. Asterisks indicate a significant difference (Student’s *t* test, *p* value < 0.05) relative to *Xe* containing an empty vector. (C) Leaves were infiltrated with bacterial suspensions (1 x 10^5^ CFU/ml) of *Xe* strains carrying a vector either empty (EV) or for expression of XopAU-HA or XopAU_K240A_-HA, and bacterial growth was quantified at the indicated dpi. A box plot displays 25^th^, 50^th^ (middle line) and 75^th^ percentiles (*n* = 5). Asterisks indicate a significant difference (Mann-Whitney U test, *p* value <0.05) relative to *Xe* containing an empty vector. Experiments were repeated at least three times with similar results.

Next, we tested whether infection of ECW30R pepper leaves with *Xe* overexpressing XopAU-HA caused activation of defense responses as observed when the effector was expressed via *Agrobacterium*. First, we monitored accumulation of PR proteins in infected leaf tissues by Western blot analysis. Both PR-2 and PR-3 accumulated at higher levels in plants inoculated with *Xe* overexpressing XopAU-HA than in plants inoculated with *Xe* carrying an empty vector or overexpressing the kinase deficient variant XopAU_K240A_-HA at 3 and 5 dpi (Fig 5A). We then assessed the mRNA levels of four genes (*PTI5*, *ACO1*, *OPR3* and *PR-1*), whose expression reflects the activation of different defense and stress pathways, at the early stages of infection (16 hours after inoculation). qRT-PCR analysis revealed that transcript levels of the *PR-1* gene, which is known to be induced by salicylic acid and pathogen attack [45], were about 30 fold higher in pepper leaves inoculated with *Xe* overexpressing XopAU-HA than in plants inoculated with *Xe* carrying an empty vector or overexpressing the kinase deficient variant XopAU_K240A_-HA (Fig 5B). The mRNA levels of the *PTI5* and *ACO1* genes, which are involved in ethylene signaling and biosynthesis, respectively [46], were not significantly altered by overexpression of XopAU-HA (Fig 5B). Finally, transcripts of the *OPR3* gene, which encodes a component of the jasmonic acid biosynthesis pathway and is induced by wounding [47], displayed only a slight induction (about 2 fold) when leaves were infected with *Xe* overexpressing XopAU-HA (Fig 5B). This analysis suggests that XopAU overexpression activated plant defense signaling. Because accumulation of PR proteins might affect the ability of bacteria to colonize the plant, we examined whether overexpression of XopAU-HA affects bacterial growth in pepper leaves. As shown in Fig 5C, *Xe* bacteria overexpressing XopAU-HA displayed a similar growth as wild-type and bacteria overexpressing XopAU_K240A_-HA up to 6 dpi, and a reduced growth only at the late stages of infection (8 dpi), which may be ascribed to high accumulation of PR proteins.

### Delivery of XopAU in plant cells by *Xcc* causes cell death

**Fig 6:**
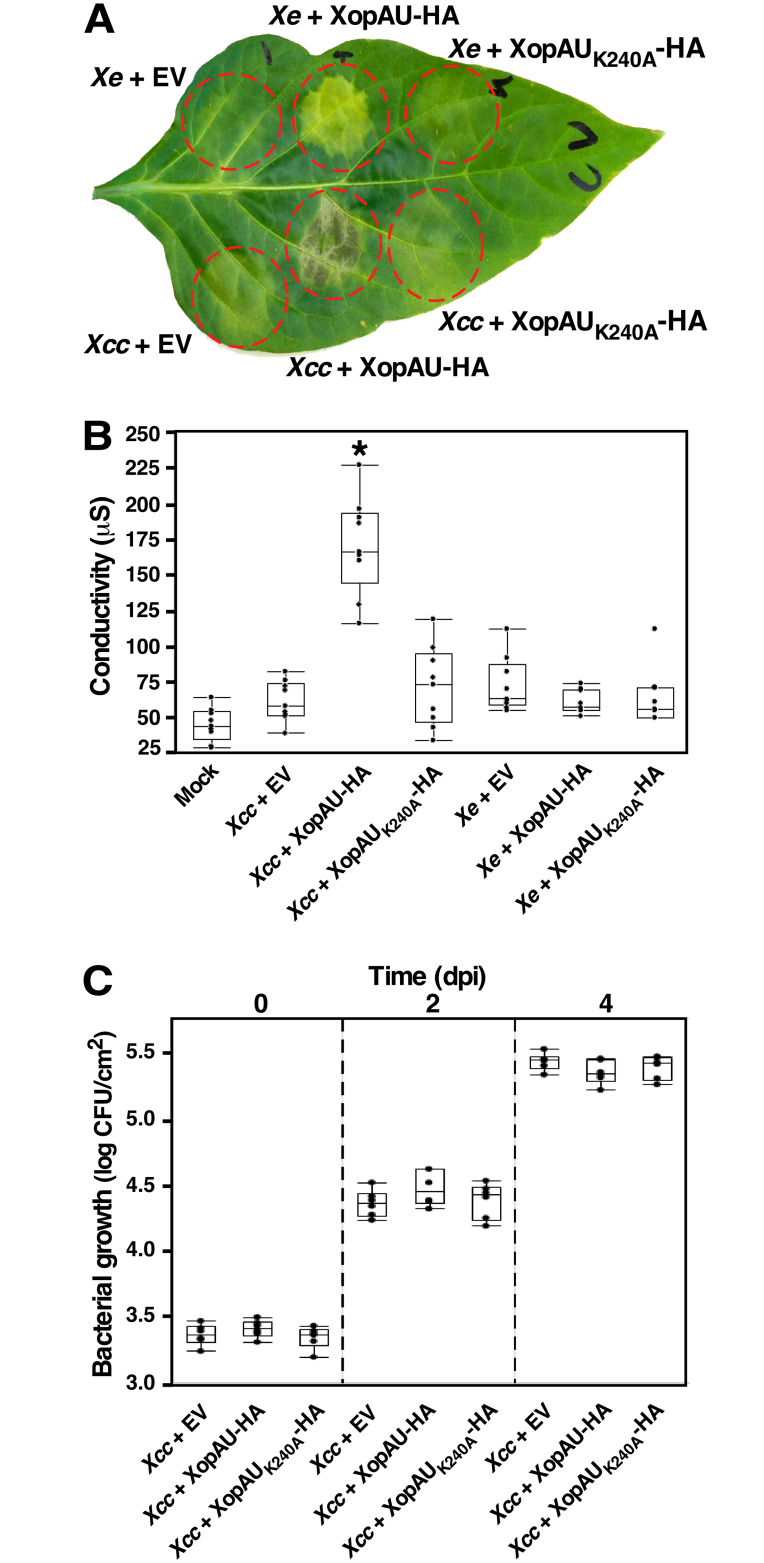
Cell death caused by delivery of XopAU in pepper cells by *Xanthomonas campestris* pv. *campestris*. Leaves of the pepper line ECW30R were syringe-infiltrated with a 10 mM MgCl_2_ mock solution (Mock) or with suspensions (1 x 10^7^ CFU/ml) of *Xe* or *Xcc* strains containing a vector for expression of XopAU-HA and XopAU_K240A_-HA, or an empty vector (EV). (A) Photograph of an inoculated leaf at 3 days post-inoculation (dpi). (B) Electrolyte leakage at 2 dpi. (C) Leaves were syringe-infiltrated with bacterial suspensions (1 x 10^5^) of *Xe* and *Xcc* strains as in (A) and bacterial growth was quantified at the indicated dpi. In (B) and (C), box plots display 25^th^, 50^th^ (middle line) and 75^th^ percentiles (in A, *n* = 7 or 9; in C, *n* = 6). An asterisk indicates a significant difference (Mann-Whitney U test, *p* value <0.05) relative to *Xe* containing an empty vector. Experiments were repeated three times with similar results.

Activation of defense responses by XopAU is accompanied by cell death when XopAU is expressed through *Agrobacterium* but not when the effector is delivered in plant cells by *Xe* bacteria. This discrepancy may be related to the interplay between XopAU and other species-specific virulence determinants. To explore this possibility, a plasmid for overexpression of XopAU-HA or XopAU_K240A_-HA was mobilized into the crucifer pathogen *Xanthomonas campestris* pv. *campestris* strain 8004 (*Xcc*), which does not encode a XopAU homolog. ECW30R pepper leaves were inoculated with *Xe* or *Xcc* strains overexpressing XopAU-HA, the kinase deficient variant XopAU_K240A_-HA or an empty vector, and cell death was monitored visually and quantified by ion leakage. Leaf areas inoculated with *Xcc* overexpressing XopAU-HA displayed cell death and a concomitant increase in ion leakage at 2 to 3 dpi, while *Xcc* containing an empty vector or overexpressing XopAU_K240A_-HA did not induce any visible phenotype (Fig 6A and 6B). At the same time, overexpression of XopAU-HA through *Xe* induced strong chlorosis (Fig 6A). To examine if the cell death induced by the expression of XopAU from *Xcc* is host specific, *Xcc* bacteria overexpressing XopAU-HA, XopAU_K240A_-HA or an empty vector were infiltrated into the leaves of *N*. *benthamiana*. Leaves inoculated with *Xcc* overexpressing XopAU-HA, but not XopAU_K240A_-HA or an empty vector, displayed cell death, which was confirmed by increased ion leakage at 24–48 h after inoculation (S7A and S7B Fig), thus indicating that this phenotype was not host specific. Overexpression of XopAU in *Xcc* did not affect bacterial growth in infected pepper and *N*. *benthamiana* leaves (Fig 6C and S7C Fig). It should be pointed out that expression of XopAU-HA and XopAU_K240A_-HA was higher in *Xcc* as compared to *Xe* bacteria as detected by Western blot analysis (S6A Fig). Together, these observations suggest that the phenotype caused by XopAU when delivered by *Xe* may be tuned by bacterial determinants absent in *Xcc* strains. Alternatively, the different phenotypes may derive from the higher expression levels of XopAU-HA in *Xcc* compared to *Xe*.

### MEK2 is required for elicitation of cell death caused by XopAU in *N*. *benthamiana* leaves

**Fig 7:**
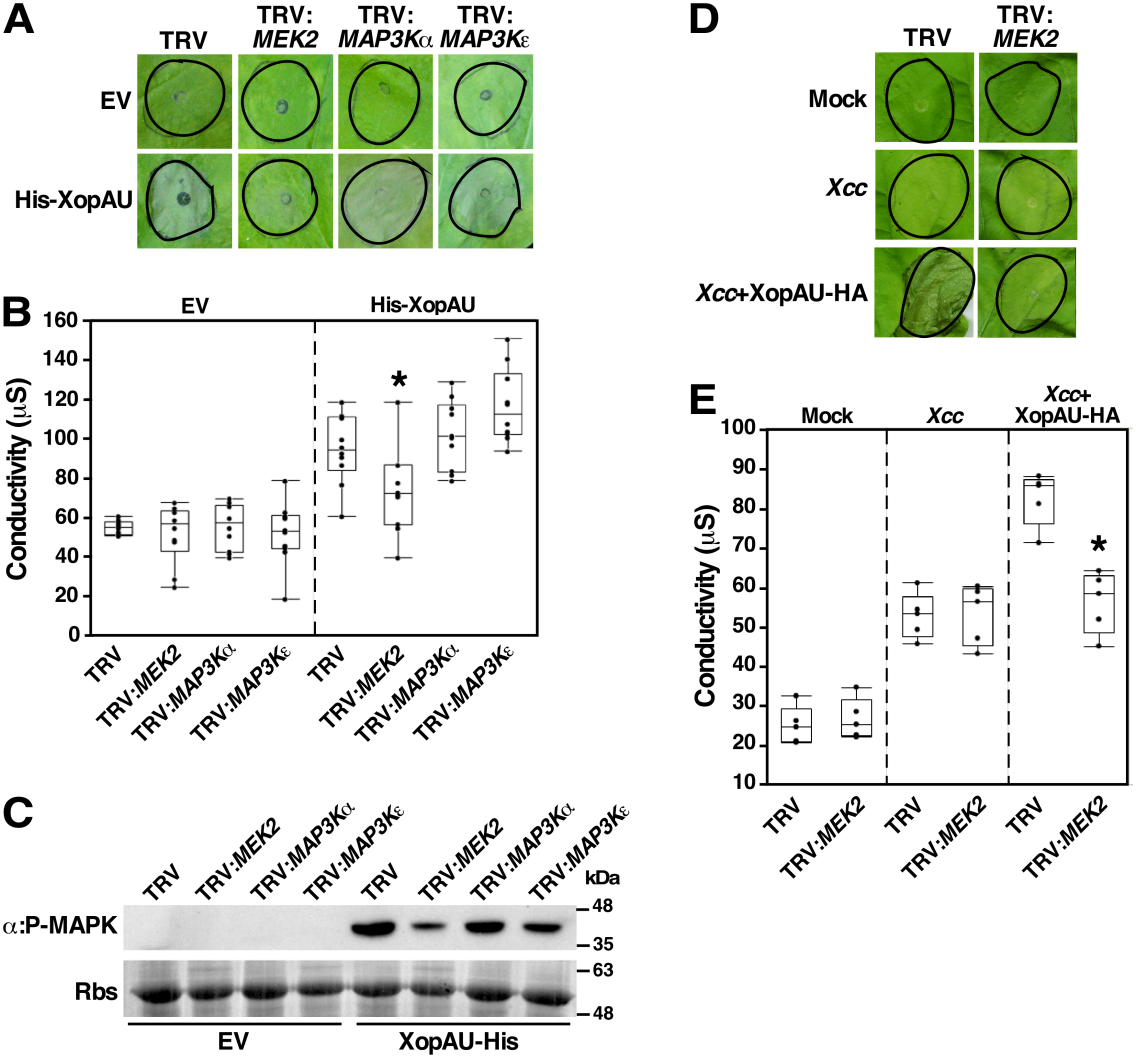
Silencing of *MEK2* in *N*. *benthamiana* reduces XopAU-induced cell death. *N*. *benthamiana* plants were infected with TRV, TRV:*MEK2*, TRV:*MAP3Kα* and TRV:*MAP3Kε*. In (A), (B), and (C), leaves of silenced plants were inoculated with *Agrobacterium* (OD_600_ = 0.02) carrying a vector either empty (EV) or for expression of His-XopAU from an estradiol-inducible system, and treated with 17β-estradiol 24 h later. (A) Photographs of inoculated areas at 36 h after 17β-estradiol application. (B) Electrolyte leakage at 24 h after 17β-estradiol application. (C) Total proteins were extracted at 12 h after 17β-estradiol application and samples were immunoblotted with α:P-MAPK antibodies. Rbs, Rubisco loading control stained by Ponceau S. In (D**)** and (E**)**, leaves of silenced plants were inoculated with mock or suspensions (5 x 10^7^ CFU/ml) of *Xcc* carrying a vector either empty (EV) or for expression of XopAU-HA. (D) Photographs of inoculated areas at 48 h after *Xcc* inoculation. (E) Electrolyte leakage at 24 h after *Xcc* inoculation. In (B**)** and (E**)**, box plots display 25^th^, 50^th^ (middle line) and 75^th^ percentiles. (in B, *n* = 10; in E, *n* = 5 or 7). Asterisks indicate a significant difference (Mann-Whitney U test, *p* value <0.05) relative to TRV empty control. Experiments were repeated three times with similar results.

MAP kinase cascades were previously shown to be involved in cell death signaling associated with plant immunity [12]. We hypothesized that XopAU induces immune responses by manipulating and activating components of MAP kinase cascades. To test this hypothesis, we examined the ability of His-XopAU to elicit cell death in *N*. *benthamiana* plants that were silenced either for the *MEK2* gene, which encodes a positive regulator of cell death, or for the *MAP3Kα* and *MAP3Kε* genes, which encode MAPKKKs acting upstream of MEK2 [9,10]. For gene silencing by VIGS, *N*. *benthamiana* plants were inoculated with *Agrobacterium* strains containing plasmids for the expression of TRV either empty or carrying a fragment of the gene to be silenced. Four weeks later, *MEK2*, *MAP3Kα* and *MAP3Kε* transcript levels were reduced by at least 75% in silenced plants as compared to TRV-infected plants (S4 Fig). At this time, *Agrobacterium* strains expressing His-XopAU were used to inoculate silenced and control plants, and cell death was monitored visually and quantified by measuring ion leakage. Cell death and ion leakage induced by His-XopAU were significantly reduced in *MEK2*-silenced plants compared to *MAP3Kα-* and *MAP3Kε*-silenced plants, and to TRV-infected plants (Fig 7A and 7B). In addition, Western blot analysis revealed that phosphorylation of MAPKs induced by His-XopAU was reduced in the *MEK2-*silenced plants (Fig 7C). Similarly, a reduction in cell death and ion leakage was also observed in *MEK2*-silenced plants challenged with *Xcc* overexpressing XopAU (Fig 7D and 7E).

**Fig 8:**
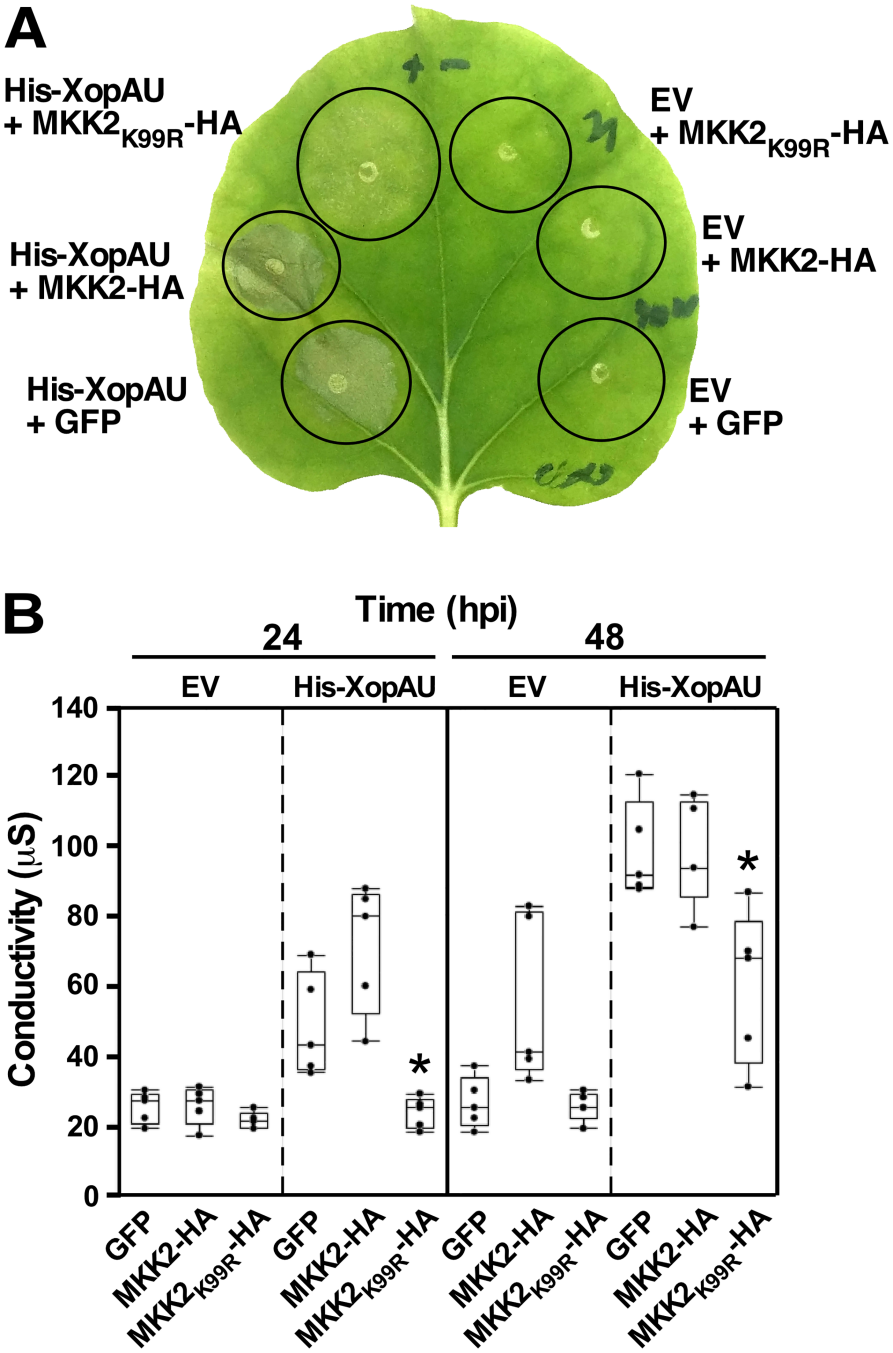
Expression of MKK2_K99R_ suppresses cell death mediated by XopAU. *N*. *benthamiana* leaves were inoculated with *Agrobacterium* to co-express His-XopAU or an empty vector (EV) with MKK2-HA, MKK2_K99R_-HA or GFP. Expression was driven by an estradiol-inducible system and 17β-estradiol was applied 24 h after agro-infiltration. (A) Photograph taken at 48 h after 17β-estradiol application. (B) Electrolyte leakage at 24 h and 48 h after 17β-estradiol application. A box plot displays 25^th^, 50^th^ (middle line) and 75^th^ percentiles. (*n* = 5). Asterisks indicate a significant difference (Mann-Whitney U test, *p* value <0.05) relative to co-expression of His-XopAU with a GFP control. The experiment was repeated three times with similar results.

To provide additional evidence that cell death induced by XopAU requires a functional MEK2, we examined whether expression of the catalytically inactive variant of the tomato MEK2 homolog MKK2 (MKK2_K99R_) causes a dominant negative effect on XopAU-mediated cell death. His-XopAU was co-expressed *via Agrobacterium* in *N*. *benthamiana* leaves with MKK2-HA, MKK2_K99R_-HA, or an unrelated protein (GFP) driven by an estradiol inducible system. Cell death was visually monitored in the inoculated leaves and quantified by measuring ion leakage at 48 h after estradiol application. Expression of MKK2_K99R_-HA, but not that of MKK2-HA or GFP, significantly reduced the cell death and ion leakage induced by His-XopAU (Fig 8) indicating that a catalytically active MEK2/MKK2 is required for XopAU-mediated cell death.

### Co-expression of XopAU and MKK2 causes a growth inhibition phenotype in yeast

**Fig 9:**
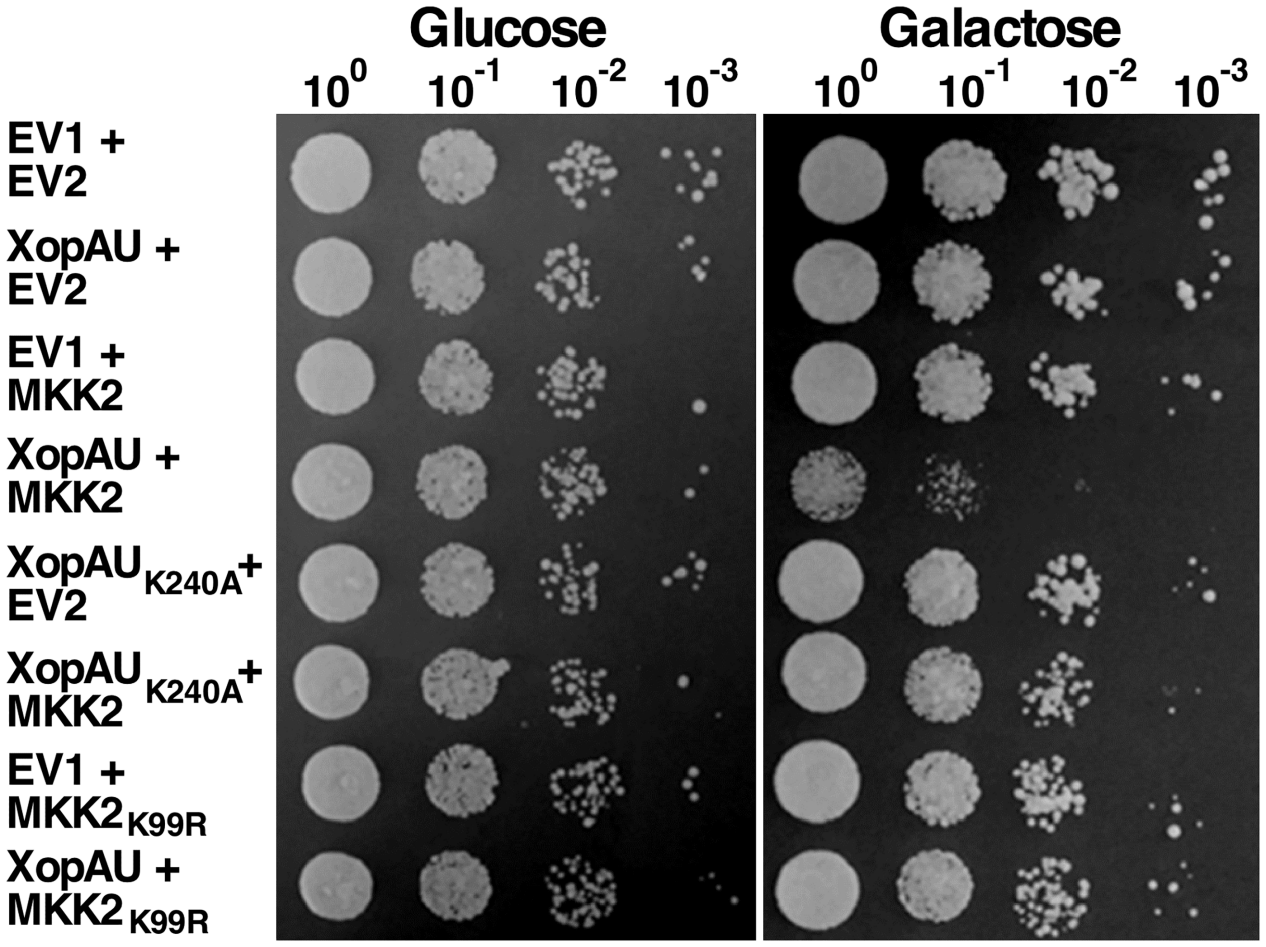
Co-expression of XopAU and MKK2 inhibits yeast growth. Yeast cultures containing the pGML10 vector either empty (EV1) or for expression of XopAU and XopAU_K240A_, and the pGMU10 vector either empty (EV2) or for expression of MKK2 and MKK2_K99R_ were normalized to OD_600_ = 0.1, and serial dilutions were spotted onto selective media containing 2% glucose or 2% galactose and 1% raffinose. Plates were incubated at 30°C for 72 h (2% glucose) or 96 h (2% galactose and 1% raffinose) and photographed. The experiment was repeated three times with similar results.

Expression of certain type III effectors in yeast has been shown to cause phenotypes that can be exploited to elucidate effector function, biochemical activity and host targets [48]. To test whether XopAU causes a detectable phenotype in the yeast *Saccharomyces cerevisiae*, the effector was fused to a c-myc tag, expressed in the yeast strain W303 driven by the *GAL1* promoter, and protein accumulation was confirmed by Western blot analysis (S6B Fig). The effect of XopAU on yeast growth was examined by serially diluting yeast cultures that carry a vector either empty or for expression of the effector and plating them onto repressing (glucose) or inducing (galactose) media. The strain expressing XopAU exhibited similar growth as the control strain containing an empty vector both in repressing and inducing media (Fig 9).

Because MEK2/MKK2 was required for XopAU-mediated phenotypes *in planta*, we hypothesized a similar requirement in yeast. To test this hypothesis, yeast were engineered to express under the control of the *GAL1* promoter either XopAU, XopAU kinase deficient (XopAU_K240A_), MKK2 and MKK2 kinase deficient (MKK2_K99R_) with an empty vector or the following protein combinations: XopAU with MKK2, XopAU with MKK2_K99R_, and XopAU_K240A_ with MKK2. All the proteins were fused to a c-myc tag and their expression was validated by Western blot analysis (S6B Fig). Yeast co-expressing XopAU with MKK2 displayed a significant reduced growth when plated on inducing medium, but not on repressing medium, as compared to yeast strains expressing each protein alone or protein combinations that included a kinase deficient variant of either XopAU or MKK2 (Fig 9). These results indicate that XopAU required MKK2 to cause growth inhibition in yeast and this phenotype was dependent on the kinase activity of both proteins.

### XopAU interacts with tomato MKK2 and MAP kinases

**Fig 10:**
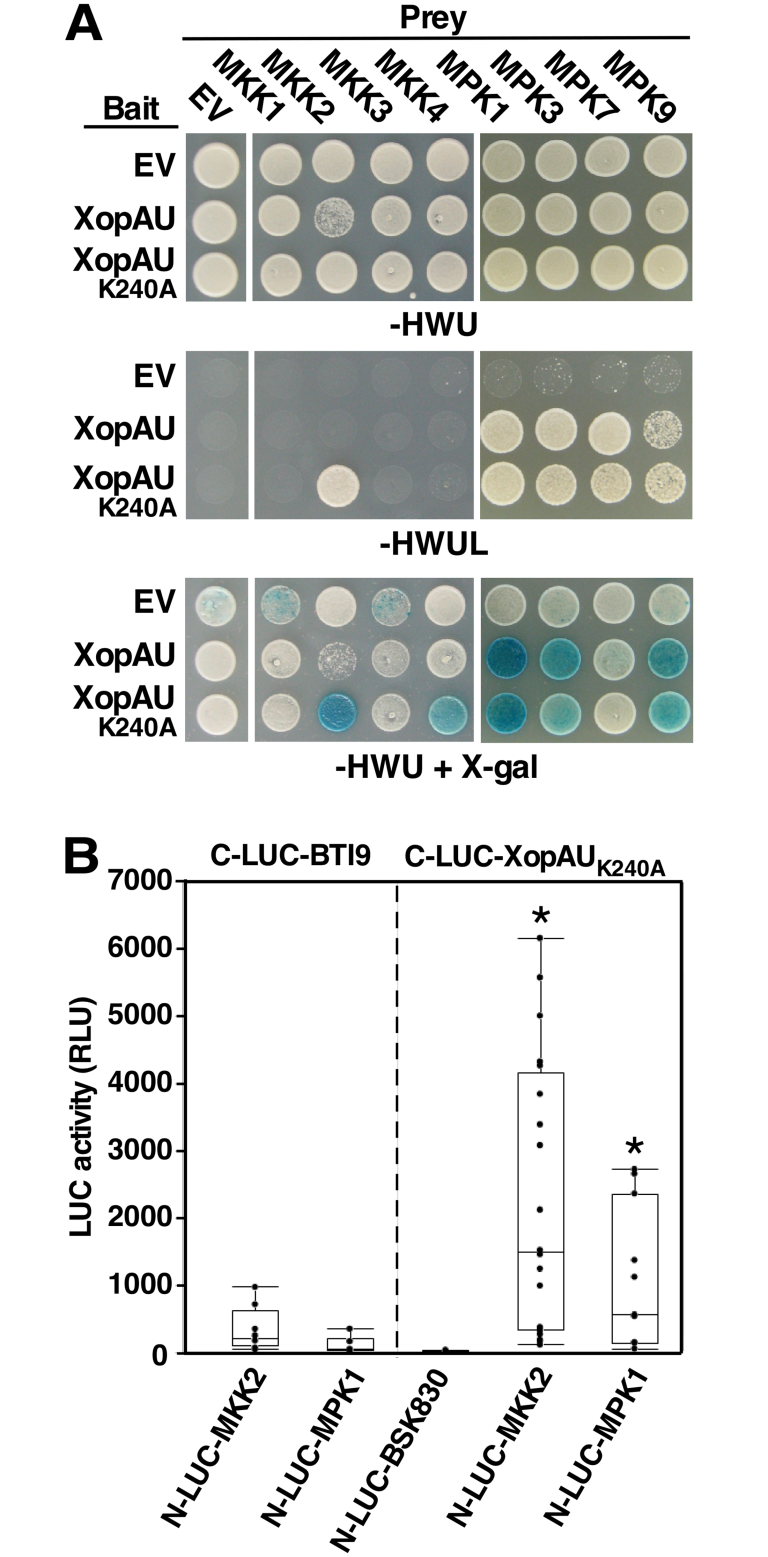
Physical interaction of XopAU with components of plant MAPK cascades in yeast and *in planta*. (A) Yeast expressing the indicated combinations of bait and prey were spotted on either selective medium (-HWUL) or non-selective medium (-HWU) with or without the addition of X-gal. (B) The indicated combinations of fusions proteins were co-expressed in epidermal cells of *N*. *benthamiana* leaves via *Agrobacterium*, and luciferase activity was quantified as relative luciferase units (RLU) at 48 h post-infiltration. C-LUC and N-LUC indicate the luciferase N-terminal (N-LUC) or C-terminal (C-LUC) region, respectively. A box plot displays 25^th^, 50^th^ (middle line) and 75^th^ percentiles. (*n* = at least 6). Asterisks indicate a significant difference (Mann-Whitney U test, *p* value <0.05) relative to C-LUC empty and C-LUC-BTI9. The experiment was repeated three times with similar results.

Because XopAU-mediated phenotypes were dependent on MEK2/MKK2 *in planta* and yeast, we hypothesized that MKK2 is a direct plant target manipulated by XopAU virulence activity. To explore this hypothesis, we tested whether XopAU physically interacts with MKK2 in a yeast two-hybrid system. To this aim, XopAU and its catalytically deficient form XopAU_K240A_ were used as bait in yeast cells that expressed the tomato MAPKKs MKK1, MKK2, MKK3 or MKK4 as preys. All bait and prey proteins were expressed in the yeast cells as confirmed by Western blot analysis (S6C and S6D Fig). While XopAU did not interact with any of the tomato MAPKKs, XopAU_K240A_ specifically interacted with MKK2, as evident by activation of the reporter genes *LEU2* and *lacZ* (Fig 10A). XopAU_K240A_ also interacted with *N*. *benthamiana* MEK2 and pepper MKK2 (S8 Fig). The lack of interaction between the catalytically active XopAU and MKK2 could be the consequence of the growth inhibition phenotype observed when both proteins were co-expressed in yeast (Fig 9).

In parallel investigation aimed at the identification of additional candidate plant targets of the effector, XopAU was used as bait in a yeast two-hybrid screen of a tomato cDNA library [49]. This screen identified three MAPKs (MPK1, MPK3, and MPK9) that consistently interacted with the kinase active and inactive forms of XopAU resulting in the activation of both reporter genes (Fig 10A and S6D Fig).

Next, we used split luciferase complementation assays to validate *in planta* protein-protein interactions that were observed in yeast. Wild-type XopAU could not be used in these experiments because it caused cell death when fused to the C-terminus of the firefly luciferase protein (C-LUC) and failed to accumulate in leaves. Instead, we used XopAU_K240A_ that was fused to C-LUC and co-expressed in *N*. *benthamiana* leaves through *Agrobacterium* along with MPK1 (representative of the XopAU-interacting MAP kinases) or MKK2 fused to the N-terminus of the firefly luciferase (N-LUC). As negative controls, C-LUC-XopAU_K240A_ was co-expressed with N-LUC fused to the tomato receptor-like cytoplasmic kinase BSK830, while N-LUC-MPK1 and N-LUC-MKK2 were co-expressed with C-LUC fused to the kinase domain of the tomato receptor-like kinase BTI9. Expression of all the fusion proteins was validated by Western blot analysis (S6E Fig). Protein-protein interactions *in planta* were quantified by measurements of luminescence at 48 h after agro-infiltration. Co-expression of C-LUC-XopAU_K240A_ and N-LUC-MPK1 or N-LUC-MKK2 resulted in emission of significantly higher luminescence compared to the negative controls indicating a physical interaction *in planta* between these two pairs of fusion proteins (Fig 10B). Together, these results indicate that XopAU physically interacts with multiple components of MAP kinase cascades at the MAPK and MAPKK levels.

### XopAU phosphorylates MKK2 *in vitro* and promotes phosphorylation of MKK2 at multiple sites *in planta*

**Fig 11:**
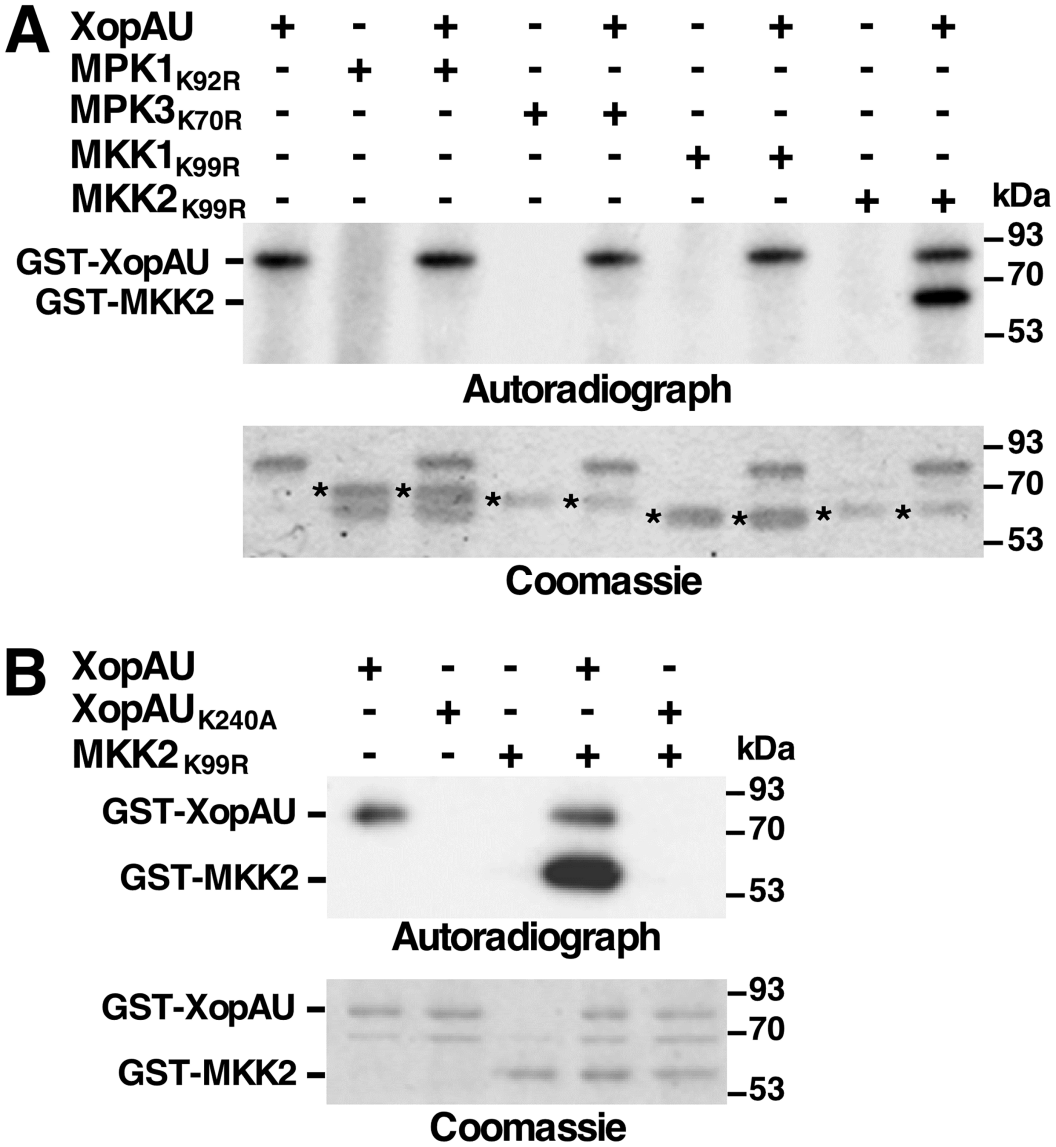
XopAU phosphorylates MKK2. *In vitro* kinase assays testing phosphorylation of XopAU-interacting proteins by XopAU (A), and MKK2_K99R_ by XopAU_K240A_ (B). The indicated proteins were incubated in a kinase assay in the presence of [γ-^32^P]ATP, fractionated by SDS-PAGE, and exposed to autoradiography (upper panel) or stained by Coomassie (lower panel). Asterisks in (A) mark bands corresponding to proteins that were tested as XopAU substrates. The experiments were repeated three times with similar results.

*In vitro* kinase assays were performed to test whether proteins that physically interacted with XopAU are substrates of XopAU phosphorylation. Kinase deficient variants of MPK1 (MPK1_K92R_), MPK3 (MPK3_K70R_), MKK2 (MKK2_K99R_) and MKK1 (MKK1_K99R_), which did not interact in yeast with XopAU and thus served as a negative control, were expressed as GST fusions in *E*. *coli*, purified and incubated with GST-XopAU in the presence of [γ-^32^P]ATP. As shown in Fig 11A, GST-XopAU phosphorylated GST-MKK2_K99R_, but not GST-MPK1_K92R_, GST-MPK3_K70R_ or GST-MKK1_K99R_. The kinase deficient GST-XopAU_K240A_ was not able to phosphorylate GST-MKK2_K99R_ confirming that labeling of GST-MKK2_K99R_ was dependent on the GST-XopAU catalytic activity (Fig 11B).

The effect of XopAU on the phosphorylation state of MKK2 was then examined *in planta*. To this aim, MKK2 tagged with a HA epitope tag (MKK2-HA) was co-expressed via *Agrobacterium* in leaves of *N*. *benthamiana* plants along with His-XopAU in the wild-type or the kinase deficient form (His-XopAU_K240A_). Expression of MKK2-HA, His-XopAU and His-XopAU_K240A_ was driven by an estradiol inducible system. MKK2-HA was immunoprecipitated from leaf samples, which were collected at 12 h after estradiol application, digested with trypsin, and analyzed by quantitative mass spectrometry of phosphopeptides. This analysis identified six residues (Thr33, Ser73, Tyr176, Thr215, Ser221 and Ser269) that were differentially phosphorylated in the presence of His-XopAU compared to His-XopAU_K240A_ (Table 1 and S4 Table). Remarkably, expression of His-XopAU resulted in an average increase of 200 fold in the phosphorylation of both Thr215 and Ser221 (Table 1 and S4 Table), which are part of the S/TxxxS/T activation motif of MKK2 [12]. Phosphorylation of Thr33, Ser73, Tyr176 and Ser269 was also enhanced upon expression of XopAU by about 36, 28, 51 and 73 folds, respectively (Table 1). Together, these observations demonstrate that XopAU phosphorylated MKK2 *in vitro* and either directly or indirectly promoted phosphorylation of multiple MKK2 sites *in planta*, possibly resulting in its activation.

**Table 1:** XopAU-mediated phosphorylation of MKK2 *in planta*.

Peptide	Site	Fold change*
RTDL**T**LPLPQR	T33	42 ± 5
TDL**T**LPLPQR	T33	30 ± 7
IG**S**GTGGTVYK	S73	28 ± 6
QVLSGL**Y**YLHR	Y176	51 ± 14
VLAQ**T**MDPCNSSVGTIAYMSPER	T215	43 ± 4
VLAQ**T**MDPCN**S**SVGTIAYMSPER	T215, S221	215 ± 118
FPF**S**VGR	S269	73 ± 46
DVDNPNVVR	-	0.6 ± 0.3
Total ion current	-	1 ± 0.2

Phosphorylated residues are underlined and bold. The ratio of the DVDNPNVVR peptide of MKK2, which does not contain any phosphorylation site, and total ion current between samples expressing His-XopAU and His-XopAU_K240A_ were determined to confirm that similar protein amounts were analyzed for the compared samples.

*Change in the amount of MKK2 phosphorylated peptides between samples expressing His-XopAU and His-XopAU_K240A_ ± SE (n = 3).

## Discussion

In this study, we uncovered biochemical properties of the *Xanthomonas euvesicatoria* type III effector XopAU that encodes a protein kinase and contributes to the development of disease symptoms in pepper plants. In addition, we show that XopAU manipulates MAP kinase signaling by activating the immunity-associated MAPKK MKK2.

This is the first report of a type III effector of phytopathogenic bacteria that encodes a catalytically active serine/threonine protein kinase representing a novel enzymatic activity for type III effectors acting within plant cells. Type III effectors with protein kinase activity were previously identified in bacterial pathogens that infect mammalian cells and they include the YpkA/YopO effector from *Yersinia* and OspG from *Shigella* [50]. YpkA/YopO phosphorylates the heterotrimeric G-protein Gα_q_ in the GTP binding loop inhibiting Gα_q_ activation and signal transduction [51]. While OspG substrates are yet to be identified, its kinase activity is required to inhibit degradation of phosphorylated IkBα and NF-κB activation induced by TNF-α stimulation, resulting in the interference of host innate immune responses [52]. Interestingly, kinase activity of both YpkA/YopO and OspG requires binding of a host factor for activation (i.e. actin and ubiquitin, respectively) possibly to prevent undesired activity while in the bacterium [53–55]. It will be interesting to test whether XopAU is a constitutively active kinase or it is activated in the plant cell by binding of a host factor or by posttranslational modifications.

Protein-protein interaction studies revealed that XopAU interacts in yeast with multiple tomato MAPKs and with the immunity-associated MAPKK MKK2. The interactions of XopAU with the MPK1 MAP kinase and MKK2 were also confirmed *in planta*. Moreover, MKK2, but not the MAPKs, was a substrate of XopAU phosphorylation *in vitro*. In several instances components of MAP kinase cascades were found to be targeted by type III effectors. With the exception of activation of the MAP kinase MPK4 by the *P*. *syringae* effector AvrB [23], other effector-MAPK interactions results in inactivation of the host MAPKs and interference with MAPK signaling. For example, members of the HopAI1 effector family of phosphothreonine lyases from plant and animal bacterial pathogens interact with MAP kinases and suppress their activities by irreversibly removing a phosphate to inhibit host immune responses [56]. Similarly, *Yersinia* YopJ and *P*. *syringae* HopF2 inhibit the signaling ability of MAPKKs by acetylation and ADP-ribosylation, respectively [19,22]. Our results demonstrate that this is not the case for XopAU. In fact, expression of XopAU *in planta* promoted phosphorylation of MAP kinases and MKK2 in their activation domains, and induced plant defense responses that are typically observed upon MKK2 activation [12]. The possibility that XopAU-mediated activation of defense responses is the result of recognition of the effector by a plant R protein is unlikely because silencing of early components of ETI signaling in *N*. *benthamiana* plants did not affect XopAU-mediated cell death.

Expression of XopAU along with MKK2 *in planta* enhanced phosphorylation of MKK2 at six residues, including Thr215 and Ser221 that are part of the S/TxxxS/T MKK2 activation motif. It remains to be established whether XopAU directly phosphorylates these residues *in planta* or activates a mechanism resulting in their phosphorylation by another kinase(s). Additional host proteins, such as the XopAU-interacting protein MPK1, MPK3 and MPK9 may be involved in the activation of MKK2 by XopAU. MPK1 might modulate XopAU activity and substrate affinity by phosphorylation, and promote phosphorylation and activation of MKK2 by XopAU. Alternatively, XopAU might enhance MPK1 phosphorylation by MKK2 by acting as a scaffold protein that interacts and bridges between MKK2 and MPK1.

Functional evidence provides further support to the biochemical data for a role of MKK2 as a target of XopAU. *In planta*, silencing of *NbMEK2*, the *N*. *benthamiana* ortholog of *MKK2*, or overexpression of a kinase dead variant of MKK2 suppressed XopAU-mediated cell death and MAPK phosphorylation indicating that MKK2 is required for XopAU molecular function. In yeast, expression of both XopAU and MKK2 in a catalytic active form, but not that of each protein alone, resulted in growth inhibition suggesting molecular cooperation between the two proteins. MKK2 does not have a closely related homolog in yeast, while its downstream MAPKs, MPK1 and MPK3, share 53% and 48% identity to FUS3 (NCBI acc. num. AAA34613.1) and HOG1 (NCBI acc. num. AJV50684.1), respectively. Interestingly, activation of either FUS3 or HOG1 pathways was reported to promote cell cycle arrest [57,58]. Based on these observations and on the finding that MKK2 is a substrate of XopAU phosphorylation *in vitro*, we hypothesize that the growth inhibition phenotype caused by co-expression of MKK2 and XopAU in yeast is a result of MKK2 activation by XopAU and subsequent MKK2 initiation of yeast MAPK cascades involved in cell cycle arrest.

Gene inactivation analysis in an attenuated *Xe* strain revealed that XopAU contributes to the appearance of disease symptoms in susceptible pepper plants, but not to bacterial growth. In addition, molecular analysis demonstrated that XopAU overexpression activates plant defense responses in *Xe* host and non-host plants. Accumulation of defense proteins reaches high levels at late stages of infection in pepper leaves infected with *Xe* overexpressing XopAU and may be the source of the decreased bacterial growth observed for this strain at 8 dpi. Remarkably, defense responses induced by XopAU were accompanied by the appearance of chlorosis when the effector was expressed by *Xe*, or by cell death when XopAU was delivered/expressed by *Agrobacterium* and *Xcc*. These different phenotypes may be related to different XopAU expression levels in the various experimental systems, as observed in *Xe* and *Xcc* bacteria overexpressing XopAU. Alternatively, the differential response observed when XopAU is delivered/expressed by different bacteria may result from inhibition of XopAU-mediated cell death by other *Xe* determinants. Possible XopAU antagonists are *Xe* 85–10 type III effectors, such as XopE1 and XopM, that are absent in *Xcc* 8004 and were found to suppress cell death induced by a constitutively active form of the immunity-associated MAPKK MEK2 [59], the tobacco ortholog of MKK2, which we report here to be activated by XopAU. Additional candidates are effectors that were shown to suppress ETI-dependent or independent cell death. For example, XopB inhibits ETI-related cell death triggered by recognition of the AvrBsT effector in pepper plants, as well as cell death induced by several other effectors in tobacco [60]. AvrBsT suppresses the ETI-related cell death induced by AvrBs1 in pepper [61], while XopJ delays the appearance of necrotic disease symptoms interfering with host salicylic acid signaling [32].

Despite the fact that MKK2 was identified as a target of XopAU, it is yet to be established how activation of this immunity-associated MKK by XopAU may contribute to *Xe* pathogenicity. MAP kinase cascades activated by tomato MKK2 and its orthologs in other plant species have been implicated as key signaling modules not only in plant immunity [8], but also in other physiological processes, such as the response to abiotic stress (*e*.*g*. wounding, osmotic and oxidative stress), stomata development and floral senescence [62–66]. It is possible that XopAU-mediated activation of MKK2 selectively induces a subset of cellular responses that are beneficial to the pathogen. Alternatively, activation of defense responses through MKK2 could be connected to the contribution of XopAU to the development of disease symptoms. In support of this hypothesis, a correlation was observed between the appearance of chlorotic symptoms and accumulation of PR proteins in pepper leaves infected with *X*e strains expressing catalytically active and inactive variants of XopAU.

In summary, we provide evidence that XopAU is a functional protein kinase that manipulates host MAPK signaling by activating the immunity-associated MAPKK MKK2. In addition, based on the different phenotypes observed when the effector is expressed by different bacteria, we propose a functional interaction between XopAU and other bacterial determinant(s). This study provides new insights about a possible role for activation of host immunity-associated MAPK cascades in disease development.

## Materials and methods

### Phylogenetic analysis

The genomic region of *Xe* 85–10 (NZ_CP017190.1) from position 4,861,200 to 4,862,753 (base pairs), which contains the ORF of the *xopAU* gene [67], was used to search homologous sequences in bacterial genomes of the non-redundant NCBI database. The *xopAU* and *gyrB* genes from a representative strain for each *Xanthomonas* species were selected for the phylogenetic analysis (S1 Table). Phylogenetic analysis was performed by using the neighbor joining method based on the *xopAU* and *gyrB* sequence alignments that were obtained by using Clustal X [68]. The bootstrap consensus tree inferred from 100 replicates is taken to represent the evolutionary history of the taxa analyzed.

### Plant material, bacterial and yeast strains

Bacterial and yeast strains used in this study are listed in S2 Table and were grown as follows: *Escherichia coli* in Lysogeny Broth (LB) medium at 37°C; *Xanthomonas euvesicatoria* (*Xe*), *Xanthomonas campestris* pv. *campestris* (*Xcc*), and *Agrobacterium tumefaciens* in LB medium at 28°C; yeast (*Saccharomyces cerevisiae*) at 30°C in selective synthetic complete medium supplemented with 2% glucose, or 2% galactose and 1% raffinose [69].

Plant cultivars used in this study are: pepper (*Capsicum annuum*) ECW20R [43] and ECW30R [70], *Nicotiana benthamiana* [71], and tomato (*Solanum lycopersicum*) Hawaii 7981 [72].

### DNA manipulation

Plasmid constructs used in this study are described in S3 Table. For cloning, DNA fragments were amplified from the *Xanthomonas* genome or cDNA of pepper, tomato or *N*. *benthamiana* plants, using Phusion DNA Polymerases (Thermo Fisher Scientific, Inc. Waltham MA, USA) or PrimeSTAR HS DNA Polymerase (Clontech Laboratories, Inc. Mountain View CA, USA). Site-directed mutagenesis was carried out using the QuikChange II kit (Agilent technologies, Inc. Santa Clara CA, USA). Sequences of oligonucleotides used in this study are available upon request.

### Mutagenesis and overexpression in *Xanthomonas* bacteria

To generate an *Xe* insertion mutant in the *avrBs2* gene (XCV0052) by single crossover, an *avrBs2* DNA fragment (187–827 bp) was cloned into the pVIK165 plasmid. The obtained plasmid was mobilized into the *Xe* or *Xe xopAU*:Gn^*R*^ [67] strains and bacteria were plated on LB media with kanamycin selection. Gene disruption was verified by PCR and loss of *Xe* avirulence in resistant ECW20R pepper plants. For overexpression of the *xopAU* gene, the *xopAU* coding region was fused to a HA epitope tag and cloned into the pBBR1MCS2 broad host vector driven by the *lac* promoter. For complementation of the *Xe xopAU*:Gn^*R*^*/avrBs2*:*Kn*^*R*^ strain, the *xopAU* gene was cloned with its native promoter (646 bp upstream to the start codon) into the pBBR1MCS-3 plasmid in reverse orientation to the *lac* promoter. Plasmids were mobilized into *Xanthomonas* strains by triparental mating [73].

### *Agrobacterium*-mediated transient expression

Binary vectors were transformed into *Agrobacterium* GV2260 by electroporation. For transient expression, *Agrobacterium* overnight cultures were pelleted, resuspended in induction medium (10 mM MgCl_2_, 10 mM MES pH 5.6, 200 mM acetosyringone), and incubated at 25°C with shaking for 4 h. Bacterial cultures were diluted to OD_600_ = 0.1 and infiltrated into leaves of *N*. *benthamiana*, pepper or tomato plants using a needleless syringe. When using the XVE estradiol-inducible system [74], plants were sprayed with an induction solution (5 μM 17β-estradiol, 1% Tween-20) at 24 h after agro-infiltration.

### Plant inoculations, measurement of bacterial growth, chlorophyll content and ion leakage

For inoculation, 7-week-old pepper or 4-weeks-old *N*. *benthamiana* and tomato plants were infiltrated with bacterial suspensions (10^5^ CFU/mL when monitoring bacterial growth; 10^7^ CFU/mL when measuring ion leakage and chlorophyll content) in 10 mM MgCl_2_ by using a needleless syringe.

For measurement of bacterial growth, three 1-cm-diameter leaf discs were sampled from at least three plants and ground in 1 mL of 10 mM MgCl_2_. Bacterial numbers were determined by plating 10 μL from 10-fold serial dilutions and counting the resulting colonies.

For measurements of chlorophyll content, 10–20 1-cm^2^ leaf disks were sampled for each treatment, placed in a tube containing 2 ml of acetone, and incubated overnight in the dark. Absorption was determined at OD_660_ and OD_642_. Total chlorophyll content was quantified with the equation: 7.12 × OD_660_ + 16.8 × OD_642_ [75]. Chlorophyll content was calculated for each inoculated leaf area relative to a mock-infiltrated area of the same leaf.

For the measurements of ion leakage, two 1.5-cm-diameter leaf disks were sampled from inoculated areas of at least five plants, and floated in 10-mL tubes containing 5 mL of double-distilled water for 4 h at 25 °C with shaking. Conductivity was measured using a DDS-12DW conductivity meter (BANTE Instruments, Shanghai, China).

### Yeast two-hybrid interactions

Yeast two-hybrid (Y2H) interactions and library screening were conducted as described [76]. To enable the use of 17β-estradiol to activate the GAL1 promoter, the yeast EGY48 strain was integrated with the Gal4-ER-VP16 transactivator [77] and renamed EGY48ES. The *xopAU* and *xopAU*_K240A_ genes were cloned into the bait plasmid pEG202 and plasmids were transformed into EGY48ES by lithium acetate transformation. Baits were tested for interactions with either a tomato cDNA library [49] or tomato proteins (S3 Table) that were fused to the pB42 transcriptional activation domain in the prey plasmid pJG4-5. Expression of prey constructs was induced by growing yeast on media supplemented with 2% glucose and 0.5 μM of 17β-estradiol. Y2H interactions were tested using the *LEU2* and *lacZ* reporter genes by plating yeast on selective media plates lacking leucine or containing x-gal, respectively.

### Yeast growth inhibition assays

The *xopAU* and *MKK2* genes were cloned into the yeast galactose inducible expression vectors pGML10 and pGMU10, respectively. Plasmids were co-transformed into the yeast strain W303 by lithium acetate transformation. For monitoring growth, yeast cultures were grown overnight at 30°C in liquid selective media containing 2% glucose, washed twice in 10 mM MgCl_2_, and normalized to OD_600_ = 0.1. Ten-fold serial dilutions were spotted (10 μl) onto repressing (2% glucose) or inducing (2% galactose and 1% raffinose) solid selective media, plates were incubated in 30°C for 72–96 h, and monitored visually for yeast growth inhibition.

### Split luciferase complementation assay

The *xopAU*, *xopAU*_K240A_, *MKK2*, *MPK1*, *BSK830* (GenBank acc. num. XP_004252882.1) and *BTI9* [78] genes were cloned in frame to firefly luciferase fragments in the binary vectors pCAMBIA:N-LUC and pCAMBIA:C-LUC [79]. The obtained vectors were transformed into *Agrobacterium* and co-expressed in *N*. *benthamiana* leaves. Luciferase activity was measured at 48 h after infiltration: 3 mm diameter leaf disks were harvested and floated in 100 μL water in a white 96-well plate. Samples were supplemented with 0.5 mM D-luciferin (Sigma-Aldrich, St. Louis MO, USA) and incubated in the dark for 10 min. Luminescence was measured using a Veritas Microplate Luminometer (Promega Corporation, Madison WI, USA).

### Expression and purification of GST fusion proteins in *E*. *coli*

*MPK1*_K92R_, *MPK3*_K70R_, *MKK2*, *MKK2*_K99R_, *MKK1*_K99R_, *xopAU* and *xopAU*_K240A_ were cloned into the pGEX-4T-1 GST fusion expression vector (GE Healthcare, Little Chalfont, UK). Plasmids were transformed into *E*. *coli* Rosetta strain (MERCK, Kenilworth NJ, USA). Bacterial cultures were grown at 37°C while shaking to OD_600_ = 0.4–0.6, supplemented with 0.1 mM Isopropyl β-D-1-thiogalactopyranoside (IPTG), and incubated overnight at 16°C with shaking. Bacteria were pelleted, resuspended in binding buffer (Tris pH 7.4 containing 1 mM PMSF, 5 μg/mL leupeptin and 5 μg/mL aprotinin), lysed using a French press and centrifuged. Supernatants were incubated with glutathione agarose (Sigma-Aldrich) and proteins were purified according to manufacturer’s instructions.

### *In vitro* kinase assay

GST fusion proteins (0.1–0.5 μg) were incubated in a kinase assay solution [50 mM Tris-HCl, pH 7.0, 1 mM dithiothreitol, 10 mM MgCl_2_, 20 μM ATP, 10 μCi [γ-^32^P]ATP (3,000 Ci/mmol; PerkinElmer, Inc. Waltham MA, USA)] at 25°C for 30 min. Reactions were stopped by the addition of SDS-sample buffer. Half of the reaction volume was fractionated on SDS-PAGE and stained with Coomassie blue. The second half was fractionated on SDS-PAGE, transferred onto a PVDF membrane, and the membrane was exposed to autoradiography.

### Virus-induced gene silencing

For TRV infection, cotyledons of one-week-old *N*. *benthamiana* plants were co-infiltrated with *Agrobacterium* containing pTRV1 and pTRV2 in 1:1 ratio as described [10]. TRV2 plasmids used for silencing are described in S3 Table. Plants were grown in a growth chamber at 20°C in long day conditions (16 h light, 8 h dark).

### RNA isolation and quantitative RT-PCR

Total RNA was isolated from leaves (50 mg) using the SV total RNA isolation system (Promega Corporation). RNA samples (2 μg) were reverse-transcribed using qScript cDNA Synthesis Kit (Quanta BioSciences, Inc. Gaithersburg MD, USA) and subjected to quantitative RT-PCR using gene specific primers (available upon request). cDNAs were amplified using the SYBR Premix Ex Taq II (Clontech Laboratories) and the Mx3000P qPCR System (Agilent technologies, Inc. Santa Clara CA, USA). The *GAPDH* gene was used for normalization, and gene expression was calculated by the comparative *C*_t_ method [80].

### Protein extraction

For protein extraction from yeast and bacteria, overnight cultures were pelleted, resuspended in lysis buffer (4% SDS, 100 mM Tris/HCl pH 7.6, 0.1 M dithiothreitol) and incubated in 95°C with SDS sample buffer for 10 min. For protein extraction from plant tissues, 3–6 leaf disks of 1 cm diameter were frozen in liquid nitrogen, homogenized in extraction buffer (100 mM Tris pH 7.4, 1% Triton X-100, 1 mM PMSF, 5 μg/mL leupeptin, 5 μg/mL aprotinin, 50 mM NaF and 1 mM Na_3_VO_4_)_,_ and centrifuged.

### Immunoprecipitation

MKK2-HA was transiently co-expressed in *N*. *benthamiana* leaves with either His-XopAU or His-XopAU_K240A_ driven by the XVE estradiol inducible system [74]. Ten gram of leaf tissues was harvested at12 h after estradiol application and ground in liquid nitrogen. Protein extraction buffer was added to the powder and samples were centrifuged. The supernatant was collected, centrifuged again, filtered through Miracloth and incubated overnight at 4°C with Monoclonal α:HA-agarose (Sigma-Aldrich) on a tube roller. HA-agarose beads were washed twice in Tris pH 7.4 and submitted to phosphopeptide mass-spectrometry analysis.

### LC-MS/MS analysis

Mass spectrometry analysis was performed at The Nancy & Stephen Grand Israel National Center for Personalized Medicine, Weizmann Institute of Science. For the identification of MKK2 sites phosphorylated *in planta* in the presence XopAU or XopAU_K240A_, immunoprecipitated samples were analyzed by LC-MS/MS as described [81]. Briefly, samples were subjected to in-solution, on-bead, trypsin digestion, and separated by using Split-less Nano Ultra Performance Liquid Chromatography (nanoUPLC; 10 kpsi NanoAcquity, Waters). The nanoUPLC was coupled online through a nanoESI emitter (10 μm tip; New Objective; Woburn, MA, USA) to a quadrupole orbitrap mass spectrometer (Q Exactive Plus, Thermo Scientific) using a FlexIon nanospray apparatus (Proxeon). For “Discovery” analysis and calculation of total ion current data was acquired in Data Dependent Acquisition (DDA) mode, using a Top 12 method [82]. For “Targeted” analysis, data was acquired in parallel reaction monitoring (PRM) mode [83], using an inclusion list containing all relevant peptides in the phosphorylated or un-modified form, as well as MKK2 peptide DVDNPNVVR. For DDA data analysis, raw data was imported into the Expressionist software (GeneData). The software was used for retention time alignment and peak detection of precursor peptides. A master peak list was generated from all MS/MS events and sent for database searching using Mascot v2.5 (Matrix Sciences). Data was searched against a protein database containing all available *Nicotianoideae* protein sequences from UniprotKB [84], *Agrobacterium tumefaciens* protein sequences, tagged MKK2 and XopAU, and 125 common laboratory contaminant proteins. Peptide identifications were filtered such that the global false discovery rate was maximum of 1%, and were then imported back to Expressionist to annotate identified peaks and calculate peptide intensities. For “Targeted” analysis [85] of MKK2 peptides, raw data were imported into the Skyline software [86]. Each peptide was manually curated to select the three most intense and reliable transitions, as well as to determine exact peak boundaries. Spectral libraries from the DDA experiments were also constructed and used in Skyline to evaluate the confidence in peak peptide assignment. Peak areas were then exported to a Microsoft Excel file, where the ratio of phosphorylated versus unmodified species of the peptide was calculated for each experiment. This ratio was then used to determine differential phosphorylation of MKK2 between samples that expressed XopAU or XopAU_K240A_ (S4 Table). The intensity of the DVDNPNVVR peptide of MKK2, which does not contain any phosphorylation site, was determined in each sample and along with total ion current of the analyzed samples was used to compare MKK2 and total protein levels.

## Supporting information

S1 Table Accession numbers and sequences of *xopAU and gyrB* homologs. (XLSX)

S2 Table Bacterial strains used in this study. (DOCX)

S3 Table Plasmids used in this study. (DOCX)

S4 Table Quantification of MKK2 peptides phosphorylated *in planta* in the presence of XopAU. (XLSX)

S1 Fig Nucleic acid sequence alignment of *xopAU* homologs. Nucleic acid sequences of *xopAU* homologs were aligned with ClustalX multiple sequence alignment tool. *S*equences and corresponding NCBI accession numbers are reported in S1 Table. A dashed line represents a gap in the alignment. Asterisks indicate nucleotide conserved in all the homologs. Nucleotides are color-coded.
(PDF)

S2 Fig Schematic representation of genomic regions flanking *xopAU* alleles in selected *Xanthomonas* strains. (A) Genomic location of the *xopAU* allelic variant of group 1 *Xanthomonas* strains in *X*. *euvesicatoria* (acc. num. NC_007508.1) and *X*. *oryzae* (acc. num. CP003057.2). (B) Genomic location of the *xopAU* group 2 allelic variant in *X*. *fragariae* (acc. num. CP016830.1) and *X*. *gardneri* (acc. num. CP018728.1), and corresponding genomic region in the *X*. *arboricola* strain (acc. num. CP012251.1), which does not contain the *xopAU* allele. Numbers and arrows represent genomic location and open reading frames (ORF), respectively. Locus tags are indicated below each ORF, which are colored based on DNA sequence homology.
(PDF)

S3 Fig Amino acid sequence alignment of XopAU homologs. Protein sequences of the XopAU homologs (S1 Table) were aligned with the COBALT multiple sequence alignment tool (https://www.ncbi.nlm.nih.gov/tools/cobalt/cobalt.cgi?) using default parameters. Blue fonts represent identical amino acids; yellow fonts represent nearly invariant residues in the protein kinase superfamily [38]. Roman numerals above the sequence indicate conserved kinase subdomains [38].
(PDF)

S4 Fig Silencing efficiency in *N*. *benthamiana* plants. *N*. *benthamiana* plants were infected with TRV, TRV:*MEK2*, TRV:*MAP3Kα*, TRV:*MAP3Kε*, TRV:*EDS1*, TRV:*NDR1*, and TRV:*RAR1*. Four weeks after infection, qRT-PCR was used to assess the expression of the targeted gene in the silenced plants relative to plants infected with empty TRV. Values are means ± SE of three biological repeats.
(TIF)

S5 Fig Inactivation of the *xopAU* gene does not affect ion leakage and bacterial growth in pepper leaves. Leaves of the pepper line ECW30R were syringe-infiltrated with a 10 mM MgCl_2_ mock solution or with suspensions (1 x 10^7^ CFU/ml) of the following *Xe* strains: *Xe* wild-type, *Xe xopAU*:*Gn*^*R*^, *Xe avrBs2*:*Kn*^*R*^, *Xe xopAU*:*Gn*^*R*^*/avrBs2*:*Kn*^*R*^, and *Xe xopAU*:*Gn*^*R*^*/avrBs2*:*Kn*^*R*^ complemented with *xopAU*. Electrolyte leakage (A) and bacterial growth (B) in the inoculated areas were quantified at the indicated days post-inoculation (dpi). The box plots display 25^th^, 50^th^ (middle line) and 75^th^ percentiles (in A, *n* = 4; in B, *n* = 5). An asterisk indicates a significant difference (Mann-Whitney U test, *p* value <0.05) compared to *Xe avrBs2*:*Kn*^*R*^.
(TIF)

S6 Fig Western blot analysis to assess protein expression in bacteria, yeast and plants. Total protein was extracted from *Xe* or *Xcc* bacteria (A), yeast (B, C and D), and *N*. *benthamiana* plants (E), separated by SDS-PAGE and immunoblotted with the indicated antibodies. In (E) asterisks indicate bands corresponding to the full-length proteins.
(TIF)

S7 Fig Cell death caused by delivery of XopAU in *N*. *benthamiana* cells through *Xanthomonas campestris* pv. *campestris* bacteria. *N*. *benthamiana* leaves were syringe-infiltrated with a 10 mM MgCl_2_ mock solution (Mock) or with suspensions (5 x 10^7^ CFU/ml) of *Xcc* strains containing a vector for expression of XopAU-HA and XopAU_K240A_-HA, or an empty vector (EV). (A) Photograph of an inoculated leaf at two days post-inoculation (dpi). Electrolyte leakage (B) and bacterial growth (C) in the inoculated areas was quantified at the indicated hours (hpi) and days post-inoculation (dpi), respectively. The box plots display 25^th^, 50^th^ (middle line) and 75^th^ percentiles (*n* = 5). An asterisk indicates a significant difference (Mann-Whitney U test, *p* value <0.05) compared to *Xcc* containing an EV.
(TIF)

S8 Fig Physical interaction of XopAU with MKK2 homologs from *N*. *benthamiana* (NbMEK2) and pepper (CaMKK2) in yeast. (A) Yeast expressing the indicated combinations of bait and prey were spotted on either selective medium (-HWUL) or non-selective medium (-HWU) with or without the addition of X-gal. (B) Western blot analysis to assess expression of NbMEK2 and CaMKK2 in yeast. Total protein was extracted from yeast, separated by SDS-PAGE and immunoblotted with α:HA antibodies.
(TIF)
