# Supplementary material for: The Xanthomonas euvesicatoria type III effector XopAU is an active protein kinase that manipulates plant MAP kinase signaling
Source: PLoS Pathog. 2018 Jan 29;14(1):e1006880. doi: 10.1371/journal.ppat.1006880 (PMC5805367; doi:10.1371/journal.ppat.1006880)
Supplement: S2 Table — (DOCX) [file ppat.1006880.s002.docx]

**S2 Table.** Bacterial and yeast strains used in this study.

| **Bacterial strain** | **Relevant characteristics** | | | **Reference or source** |
| --- | --- | --- | --- | --- |
| **Escherichia coli** |  | | |  |
| DH5α | RecA, lacZΔ, M15 | | | Invitrogen, San Diego, CA, USA |
| S-17-1 λpir | RK2 tra regulon, pir, host for pir-dependent plasmid pVIK165 | | | [[1](#_ENREF_1)] |
| Rosetta | BL21 derivative, for expression of fusion proteins | | | MERCK, Kenilworth  NJ, USA |
| ***Xanthomonas euvesicatoria*** | |  |  |  |
| 85-10 | *Rif^R^ | | | [[2](#_ENREF_2)] |
| 85-10 *avrBs2:Kn^R^* | 85-10 derivative containing insertion in *avrBs2* (XCV0052)*,* Kn^R^, Rif^R^ | | | This study |
| 85-10 *xopAU:Gn^R^* | 85-10 derivative containing insertion in *xopAU* (XCV1196), Gn^R^, Rif^R^ | | | [[3](#_ENREF_3)] |
| 85-10 *xopAU*:Gn^R^/*avrBs2:Kn^R^* | 85-10 derivative containing insertions in *xopAU* and *avrBs2,* Gn^R^, Kn^R^, Rif^R^ | | | This study |
| ***Xanthomonas campestris* pv. *campestris*** | | |  |  |
| 8004 | Rif^R^ | | | [[4](#_ENREF_4)] |
| **Agrobacterium tumefaciens** |  | | |  |
| GV2260 | C58 background, carries Ti plasmid pGV2260 (pTiB6S3DT-DNA), Rif^R^, Ap^R^ | | | [[5](#_ENREF_5)] |
| **Yeast strain** |  | | |  |
| EGY48 | *MATa, his3, trp1, ura3, LexAop*(x6)-*LEU2* | | | Invitrogen, San Diego, CA, USA |
| EGY48ES | EGY48 derivative, expression Gal4-ER-VP16 under estradiol inducible promoter | | | This study & [[6](#_ENREF_6)] |
| W303 | *MAT*a/*MAT*α *leu2-3,112 trp1-1 can1-100 ura3-1 ade2-1 his3-11,1* | | | [[7](#_ENREF_7)] |

*Rif^R^, Kn^R^, Gn^R^ and Ap^R^ indicate resistance to rifampicin, kanamycin, gentamicin, and ampicillin, respectively.

**REFERENCES**

1. Simon R, Priefer U, Puhler A. A broad host range mobilization system for *in* *vivo* genetic engineering: transposon mutagenesis in gram negative bacteria. Nat Biotechnol 1983; 1: 784-791.

2. Thieme F, Koebnik R, Bekel T, Berger C, Boch J, et al. Insights into genome plasticity and pathogenicity of the plant pathogenic bacterium *Xanthomonas campestris* pv. *vesicatoria* revealed by the complete genome sequence. J Bacteriol 2005; 187: 7254-7266.

3. Teper D, Burstein D, Salomon D, Gershovitz M, Pupko T, et al. Identification of novel *Xanthomonas euvesicatoria* type III effector proteins by a machine‐learning approach. Mol Plant Pathol 2015; 17: 398-411.

4. Turner P, Barber C, Daniels M. Evidence for clustered pathogenicity genes in *Xanthomonas campestris* pv. *campestris*. Mol Gen Genet 1985; 199: 338-343.

5. Deblaere R, Bytebier B, De Greve H, Deboeck F, Schell J, et al. Efficient octopine Ti plasmid-derived vectors for *Agrobacterium*-mediated gene transfer to plants. Nucleic Acids Res 1985; 13: 4777-4788.

6. Quintero MJ, Maya D, Arévalo-Rodríguez M, Cebolla Á, Chávez S. An improved system for estradiol-dependent regulation of gene expression in yeast. Microb Cell Fact 2007; 6: 10.

7. Ralser M, Kuhl H, Ralser M, Werber M, Lehrach H, et al. The *Saccharomyces cerevisiae* W303-K6001 cross-platform genome sequence: insights into ancestry and physiology of a laboratory mutt. Open Biol 2012; 2: 120093.
