# Supplementary material for: The Xanthomonas euvesicatoria type III effector XopAU is an active protein kinase that manipulates plant MAP kinase signaling
Source: PLoS Pathog. 2018 Jan 29;14(1):e1006880. doi: 10.1371/journal.ppat.1006880 (PMC5805367; doi:10.1371/journal.ppat.1006880)
Supplement: S3 Table — (DOCX) [file ppat.1006880.s003.docx]

**S3 Table.** Plasmids used in this study.

| **Plasmid** | **Characteristics** | **Reference or source** |
| --- | --- | --- |
| pER8 | Binary vector for 17β-estradiol inducible expression driven by the XVE system. Sp^R^ | [[1](#_ENREF_1)] |
| pER8:*His-xopAU* | For estradiol inducible transient expression. Sp^R^ | This study |
| pER8:*His-xopAU*_K240A_ | For estradiol inducible transient expression. Sp^R^ | This study |
| pER8:*MKK2*-*HA* | For estradiol inducible transient expression of tomato *MKK2* (NCBI acc. NP_001234588.1). Sp^R^ | [[2](#_ENREF_2)] |
| pER8:*MKK2*-*HA*(K99R) | For estradiol inducible transient expression of tomato *MKK2*. Sp^R^ | [[2](#_ENREF_2)] |
| pTRV1 (pYL192) | VIGS TRV RNA1-based vector. Kn^R^ | [[3](#_ENREF_3)] |
| pTRV2 (pYL170) | VIGS TRV RNA2-based vector. Kn^R^ | [[3](#_ENREF_3)] |
| pTRV2:*NbMEK2* | TRV2 containing the 878-1,119 bp fragment of *NbMEK2* (NCBI acc. num. AB360636.1). Kn^R^ | [[4](#_ENREF_4)] |
| pTRV2:*NbMAP3Kα* | TRV2 containing the 1-322 bp fragment of *NbMAP3Kα* (NCBI acc. num. AY500155.1). Kn^R^ | [[5](#_ENREF_5)] |
| pTRV2:*NbMAP3Kε* | TRV2 containing the 898-1308 bp fragment of *NbMAP3Kε* (NCBI acc. num. GU205153.1). Kn^R^ | [6] |
| pTRV2:*NbEDS1* | TRV2 containing the 1187-1909 bp fragment of NtEDS1 (NCBI acc. num. AF479625). KnR | This Study |
| pTRV2:*NbNDR1* | TRV2 containing the 218-779 bp fragment of *NbNDR1* (NCBI acc. num. AY438029). Kn^R^ | This Study |
| pTRV2:*NtRAR1* | TRV2 containing the 186-654 bp fragment of *NtRAR1* (NCBI acc. num. AF480487). Kn^R^ | [7] |
| p*Cf4* | Plasmid used for transient expression of *Cf-4* in *N. benthamiana*. Expression driven by 35S promoter. Kn^R^ | [8] |
| p*Avr4* | Plasmid used for transient expression of *Avr4* in *N. benthamiana*. Expression driven by 35S promoter. Kn^R^ | [8] |
| pCAMBIA1300:*N-LUC* | Split luciferase transient expression system. Expression of the N-terminal 416 aa of firefly luciferase driven by the CaMV 35S promoter. Kn^R^ | [9] |
| pCAMBIA1300:*C-LUC* | Split luciferase transient expression system. Expression of the C-terminal 398-550 aa of firefly luciferase driven by the CaMV 35S promoter. Kn^R^ | [9] |
| pCAMBIA1300:*N-LUC-SlMKK2*  **Table S3:** Plasmids used during this study | For transient expression of *N-LUC* fused to tomato *MKK2* driven by the CaMV 35S promoter. Kn^R^ | This study |
| pCAMBIA1300:*N-LUC*-*SlMPK1* | For transient expression of *N-LUC* fused to tomato *MPK1* (NCBI acc. num. AY261512.1) driven by the CaMV 35S promoter. Kn^R^ | This study |
| pCAMBIA1300:*N-LUC-SlBSK830* | For transient expression of *N-LUC* fused to C-terminal region of tomato *BSK830* (NCBI acc. num. XP_004252882.1) driven by the CaMV 35S promoter. Kn^R^ | This study |
| pCAMBIA1300:*xopAU-C-LUC* | For transient expression of *Xe* effector *xopAU* fused to *C-LUC* driven by the CaMV 35S promoter. Kn^R^ | This study |
| pCAMBIA1300:*xopAU*_K240A_*-C-LUC* | For transient expression of *Xe* effector *xopAU*_K240A_ fused to *C-LUC* driven by the CaMV 35S promoter. Kn^R^ | This study |
| pCAMBIA1300:*SlBTI9-C-LUC* | For transient expression of the 991-1881 bp fragment of tomato *BTI9* (NCBI acc. num. NP_001233773.1) fused to *C-LUC* driven by the CaMV 35S promoter. Kn^R^ | This study |
| pRK2073 | Helper plasmid for triparental matings. Sp^R^ | [10] |
| pBBR1MCS-2 | Bacterial broad-host-range vector. *lac* promoter is upstream to MCS. Kn^R^ | [11] |
| pBBR1MCS-3 | Bacterial broad-host-range vector. *lac* promoter is upstream to MCS. Tet^R^ | [11] |
| pBBR1MCS-2:*xopAU* | For *lac* driven expression of the *Xe* 85-10 effector *xopAU* in *Xe* and *Xcc*. Kn^R^ | This study |
| pBBR1MCS-2:*xopAU*_K240A_ | For *lac* driven expression of the *Xe* 85-10 effector *xopAU*_K240A_ in *Xe* and *Xcc*. Kn^R^ | This study |
| pBBR1MCS-3 promoter + *xopAU* | For native promoter driven expression of the *Xe* 85-10 effector *xopAU*. *xopAU* plus 5' 646 bp was cloned in reverse orientation to *lac* promoter. Tet^R^ | This study |
| pVIK165 | Suicide plasmid, R6K ori. Kn^R^ | [12] |
| pVIK165:*avrBs2* (187-827) | Containing the 187-827 bp fragment of *avrBs2*. Kn^R^ | This study |
| pGEX-4T-1 | GST-fusion expression vector. Ap^R^ | GE healthcare, Little Chalfont, UK |
| pGEX-4T-1:*xopAU* | For expression in *E. coli*. Ap^R^ | This study |
| pGEX-4T-1:*xopAU* _K240A_ | For expression in *E. coli*. Ap^R^ | This study |
| pGEX-4T-1:*SlMKK2*_K99R_ | For expression in *E. coli*. Ap^R^ | This study |
| pGEX-4T-1:*SlMKK2*_K99R/T33A_ | For expression in *E. coli*. Ap^R^ | This study |
| pGEX-4T-1:*SlMKK1*_K99R_ | For expression in *E. coli*. Ap^R^ | This study |
| pGEX-4T1:*SlMPK1*_K92R_ | For expression in *E. coli*. Ap^R^ | This study |
| pGEX-4T1:*SlMPK3*_K70R_ | For expression in *E. coli*. Ap^R^ | [13] |
| pGML10 | Yeast galactose inducible expression vector. Ap^R^, Leu | [14] |
| pGMU10 | Yeast galactose inducible expression vector. Ap^R^, Ura | [14] |
| pGML10:*xopAU* | For expression in yeast. Ap^R^, Leu | This study |
| pGML10:*xopAU*_K240A_ | For expression in yeast. Ap^R^, Leu | This study |
| pGMU10:*SlMKK2* | For expression in yeast. Ap^R^, Ura | This study |
| pGMU10:*SlMKK2*_K99R_ | For expression in yeast. Ap^R^, Ura | This study |
| pEG202 | Y2H bait vector. LexA DNA binding domain. Ap^R^. His | Invitrogen, San Diego, CA, USA |
| pJG4-5 | Y2H prey vector. B42 activation domain fused to HA tag is expressed under the galactose inducible *GAL1*promoter. Ap^R^. Trp | Invitrogen, San Diego, CA, USA |
| pSH18-30 | Y2H *lacZ* reporter vector. Express *LacZ* under the LexA operator. Ap^R^, Ura | Invitrogen, San Diego, CA, USA |
| pEG202:*xopAU* | Expression of *lexA*-*xopAU* fusions for testing Y2H interactions. Ap^R^. His | This study |
| pEG202:*xopAU*_K240A_ | Expression of *lexA*-*xopAU* (K240A) fusions for testing Y2H interactions. Ap^R^. His | This study |
| pJG4-5:*SlMPK1* | Galactose inducible expression of *B42AD-SlMPK1* (NCBI acc. num. AY261512.1) fusion for testing Y2H interactions. Ap^R^. Trp | This study |
| pJG4-5:*SlMPK3* | Galactose inducible expression of *B42AD-SlMPK3* (NCBI acc. num. NM_001247431.1) fusion for testing Y2H interactions. Ap^R^. Trp | This study |
| pJG4-5:*SlMPK7* | Galactose inducible expression of *B42AD-SlMPK7* (NCBI acc. num. NM_001246968) fusion for testing Y2H interactions. Ap^R^. Trp | This study |
| pJG4-5:*SlMPK9* | Galactose inducible expression of *B42AD-SlMPK9* (NCBI acc. num. NM_001246832) fusion for testing Y2H interactions. Ap^R^. His | This study |
| pJG4-5:*SlMKK1* | Galactose inducible expression of *B42AD-SlMKK1* (NCBI acc. num. NM_001247815.1) fusion for testing Y2H interactions. Ap^R^. Trp | This study |
| pJG4-5:*SlMKK2* | Galactose inducible expression of *B42AD-SlMKK2* (NCBI acc. num. NP_001234588.1) fusion for testing Y2H interactions. Ap^R^. Trp | This study |
| pJG4-5:*SlMKK3* | Galactose inducible expression of *B42AD-SlMKK3* (NCBI acc. num. NM_001247662.1) fusion for testing Y2H interactions. Ap^R^. Trp | This study |
| pJG4-5:*SlMKK4* | Galactose inducible expression of *B42AD-SlMKK4* (NCBI acc. num. NM_001247666.1) fusion for testing Y2H interactions. Ap^R^. Trp | This study |

Rif^R^, Kn^R^, Gn^R^, Tet^R^ and Ap^R^ indicate resistance to rifampicin, kanamycin, gentamicin, tetracycline and ampicillin, respectively.

Trp, His, Leu, and Ura indicate that the vector provide yeast with autotrophy to tryptophan, histidine, leucine and uracil, respectively.

**REFERENCES**

1. Zuo J, Niu QW, Chua NH. An estrogen receptor‐based transactivator XVE mediates highly inducible gene expression in transgenic plants. Plant J 2000; 24: 265-273.

2. Pedley KF, Martin GB. Identification of MAPKs and their possible MAPK kinase activators involved in the Pto-mediated defense response of tomato. J Biol Chem 2004; 279: 49229-49235.

3. Liu Y, Schiff M, Dinesh-Kumar SP. Virus-induced gene silencing in tomato. Plant J 2002; 31: 777-786.

4. Ekengren SK, Liu Y, Schiff M, Dinesh-Kumar SP, Martin GB. Two MAPK cascades, NPR1, and TGA transcription factors play a role in Pto-mediated disease resistance in tomato. Plant J 2003; 36: 905-917.

5. del Pozo O, Pedley KF, Martin GB. MAPKKKα is a positive regulator of cell death associated with both plant immunity and disease. EMBO J 2004; 23: 3072-3082.

6. Melech‐Bonfil S, Sessa G. Tomato MAPKKKε is a positive regulator of cell‐death signaling networks associated with plant immunity. Plant J 2010; 64: 379-391.

7. Liu Y, Schiff M, Marathe R, Dinesh‐Kumar SP. Tobacco Rar1, EDS1 and NPR1/NIM1 like genes are required for N‐mediated resistance to tobacco mosaic virus. The Plant Journal. 2002; 30:415-429.

8. Van der Hoorn RA, Laurent F, Roth R, De Wit PJ. Agroinfiltration is a versatile tool that facilitates comparative analyses of Avr 9/Cf-9-induced and Avr 4/Cf-4-induced necrosis. Molecular Plant-Microbe Interactions. 2000;13:439-446.

9. Chen H, Zou Y, Shang Y, Lin H, Wang Y, et al. Firefly luciferase complementation imaging assay for protein-protein interactions in plants. Plant Physiol 2008; 146: 368-376.

10. Leong SA, Ditta GS, Helinski DR. Heme biosynthesis in Rhizobium. Identification of a cloned gene coding for delta-aminolevulinic acid synthetase from *Rhizobium meliloti*. J Biol Chem 1982; 257: 8724-8730.

11. Kovach ME, Elzer PH, Hill DS, Robertson GT, Farris MA, et al. Four newderivatives of the broad-host-range cloning vector pbbr1mcs, carrying different antibiotic-resistance cassettes. Gene 1995; 166: 175-176.

12. Kalogeraki VS, Winans SC. Suicide plasmids containing promoterless reporter genes can simultaneously disrupt and create fusions to target genes of diverse bacteria. Gene 1997; 188: 69-75.

13. Mayrose M, Bonshtien A, Sessa G. LeMPK3 is a mitogen-activated protein kinase with dual specificity induced during tomato defense and wounding responses. J Biol Chem 2004; 279: 14819-14827.

14. Iha H, Tsurugi K. Shuttle-vector system for *Saccharomyces cerevisiae* designed to produce C-terminal-Myc-tagged fusion proteins. Biotechniques 1998; 25: 936-938.
