# Supplementary material for: The Xanthomonas euvesicatoria type III effector XopAU is an active protein kinase that manipulates plant MAP kinase signaling
Source: PLoS Pathog. 2018 Jan 29;14(1):e1006880. doi: 10.1371/journal.ppat.1006880 (PMC5805367; doi:10.1371/journal.ppat.1006880)
Supplement: S1 Fig — Nucleic acid sequences of xopAU homologs were aligned with ClustalX multiple sequence alignment tool. Sequences and corresponding NCBI accession numbers are reported in S1 Table. A dashed line represents a gap in the alignment. Asterisks indicate nucleotide conserved in all the homologs. Nucleotides are color-coded. (PDF) [file ppat.1006880.s005.pdf]

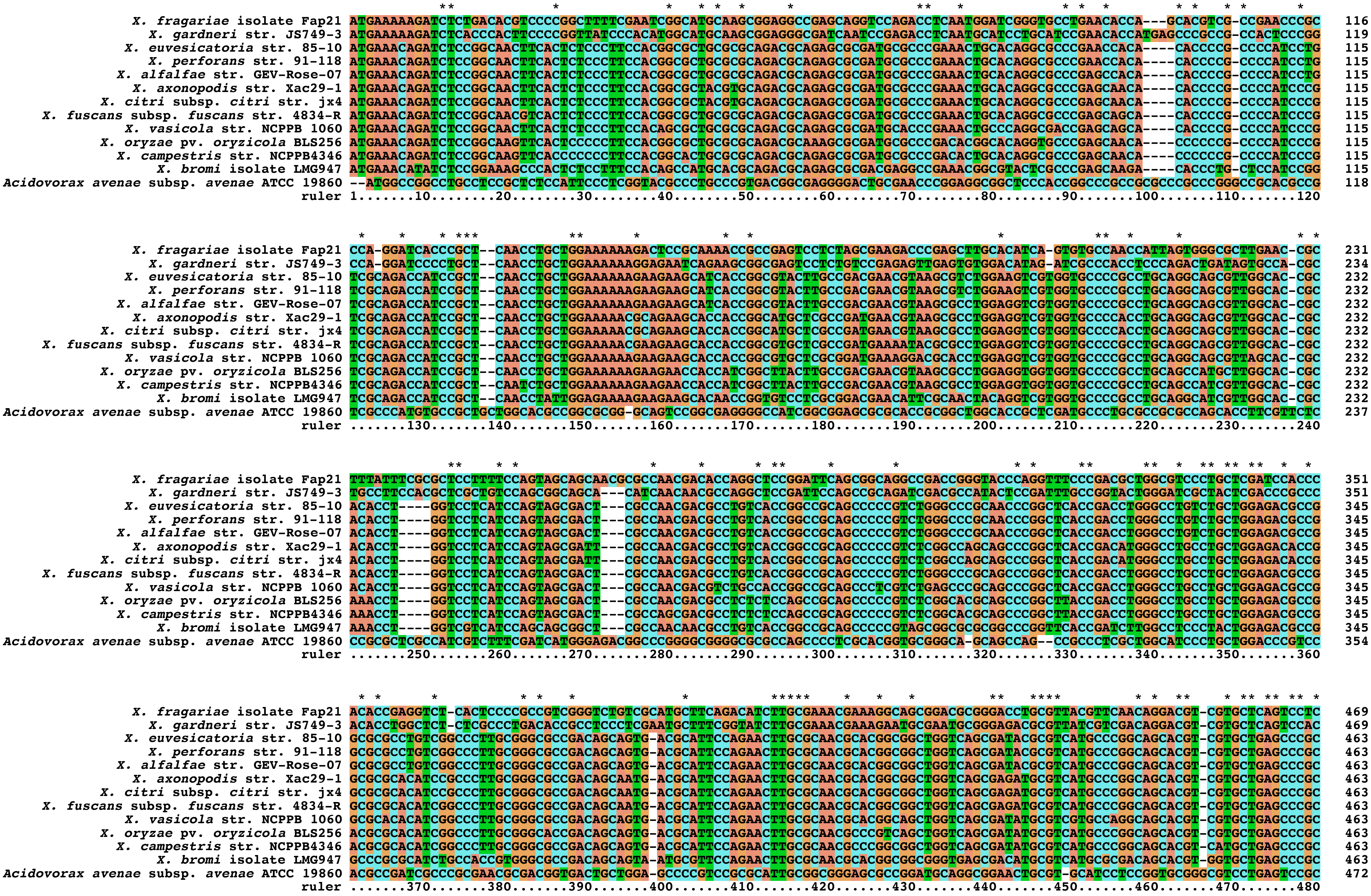

X. fragariae isolate Fap21
X. gardneri str. JS749-3
X. euvesicatoria str. 85-10
X. perforans str. 91-118
X. alfalfae str. GEV-Rose-07
X. axonopodis str. Xac29-1
X. citri subsp. citri str. jx4
X. fuscans subsp. fuscans str. 4834-R
X. vasicola str. NCPPB 1060
X. oryzae pv. oryzicola BLS256
X. campestris str. NCPPB4346
X. bromi isolate LMG947
Acidovorax avenae subsp. avenae ATCC 19860
ruler

```
                                                 *        *   *  *    *     *    **   *          *  *     **** ** ** **    **  *  *    ** **       **      * *
                X. fragariae isolate Fap21 GCACAGTAAGGTCTCTCGCCGCCAAGTCTCAGCGTCCAGAAGTGATCGAAAGACTGTCGACATTGAAACCTGGTGCGCTGGAAATCGAGAACGGCTTCCTCCCTGTGCGGTTGGAGCGAG  589
                  X. gardneri str. JS749-3 GCACACTAAAGGACCTCGCCAGCAATTCTCCGCGTCCAGAGCTGCTCACGAAATTATCACTCTTGAAACCTGGTGCGCTGGAAATCGAGAATGGCTTCCTCCCTGTACGGCTTGAAGGAA  589
               X. euvesicatoria str. 85-10 GCCTGGTGAAATCGCTGACCAGCCGCTCCGAACGCCCGGACTTGATCCAGAAGCTCTCGGCGTTGAAGCCCGGCGCCCTGGATGTAGACAATGGATTTCTCCACGTAACCCTGGAAACCG  583
                  X. perforans str. 91-118 GCCTGGTGAAATCGCTGACCAGCCGCTCCGAACGCCCGGACTTGATCCAGAAGCTCTCGGCGTTGAAGCCCGGCGCCCTGGATGTAGACAATGGATTTCTCCACGTAACCCTGGAAACCG  583
              X. alfalfae str. GEV-Rose-07 GCCTGGTGAAATCGCTGACCAGCCGCTCCGAACGCCCGGACTTGATCCAGAAGCTCTCGGCGTTGAAGCCCGGCGCCCTGGATGTAGACAATGGATTTCTCCACGTAACCCTGGAAACCG  583
                X. axonopodis str. Xac29-1 GTCTGGTGAAATCGCTGACCAGCCGATCCGAACGCCCGGACTTGATCCAGAAGTTATCGGCGTTGAAGCCAGGCGCCCTGGATGTAGACACTGGTTTTCTCAAGGTGACGCTGGAAACCG  583
            X. citri subsp. citri str. jx4 GTCTGGTGAAATCGCTGACCAGCCGATCCGAACGCCCGGACTTGATCCAGAAGTTATCGGCGTTGAAGCCAGGCGCCCTGGATGTAGACACTGGTTTTCTCAAGGTGACGCTGGAAACCG  583
     X. fuscans subsp. fuscans str. 4834-R GTCTGGTGAAATCGCTGACCAGCCGATCCGAACGCCCGGACCTGATCCAGAAGTTATCGGCGTTGAAGCCAGGCGCCCTGGATGTAGACACTGGTTTTCTCCAGGTGACGCTGGAAACCG  583
               X. vasicola str. NCPPB 1060 GTCTGGTGAAATCGCTGACCAGCCGATCCGAACGCCCGGACTTGATCCAGAAGCTCTCGGCGTTGAAGCCCGGCGCCTCGGATGTAGATGGTGGGTTTCTCCACGTGACGCTGGAAACCG  583
            X. oryzae pv. oryzicola BLS256 GCCTGGTGAAATCGCTGACCAGCCGATCCGAACGCCCGGACTTGATCCAGAAGCTATCGGCGTTGAAGCCCGGCGCCCTGGATGTAGACAGTGGGTTTGTCCGCGTGACGCTGGAAACCG  583
              X. campestris str. NCPPB4346 GCCTGGTGAAATCGCTGACCAGCCGATCCGAACGCCCGGACTTGATCCAGAAGCTATCGGCGTTGAAGCCCGGCGCCCTGGATGTAGACAGTGGGTTTGTCCGCGTGACGCTGGAAACCG  583
                   X. bromi isolate LMG947 GTTTGGTGAACTCGCTGACCAGCCGATCCGAACGCCCGGACTTGATTCTCAAACTATCGGCCTTGAAGCCCGGCGCGTTGGATGTGGACGGCGGCTTTCTCCAGGTGACACTGGGAACCC  583
Acidovorax avenae subsp. avenae ATCC 19860 ACGTGGTGCATTCCATGGGCAGCCCGCCCG---GCTGGGAAGCGC------GGCTGGCGGCGCTGAAGCCGGGCGCGGCCGAGGTGCAGCAGGGGTTCGAGCGGGTCGATCTCGACACGG  583
                                     ruler .......490.......500.......510.......520.......530.......540.......550.......560.......570.......580.......590.......600

                                                    *             *        *** *      ****    ***** ****        ** ** ** ** *** *     ** *   **  *  *   *  *    **
                X. fragariae isolate Fap21 CCCACGACAGCGATCCTCGAACCCTTCAGGGCATG-CGGCCGATCGGCAGTGGCGCTTCCGGGTCGGCCTATGCTGTGCGGCTGGCTGAAGATTTCTGGCGTGCTGGAGAGAATTGCGGG  708
                  X. gardneri str. JS749-3 CGCACAACGGCGACACCCAAAACCTCAAGGACATG-CAGCCACTCGGAAAAGGCGCTTCCGGGCGAGCCTATGCTGTGCGTCTGGCTGAGGATTTCTGGCGTGGCGAAGAAAATTGCGGG  708
               X. euvesicatoria str. 85-10 GGCACGCAGGCACTGCAGGAACGCTGGACGCCATG-CCCACCGTCGGCACCGGCGCATCCGCCTCGACCTACGCGGTGCGGCTGGCCGAAGATCTTTGGCAGGGCGGGCAAAATTGCGGA  702
                  X. perforans str. 91-118 GGCACGCAGGCACTGCAGGAACGCTGGACGCCATG-CCCACCGTCGGCACCGGCGCATCCGCCTCGACCTACGCGGTGCGGCTGGCCGAAGATCTTTGGCAGGGCGGGCAAAATTGCGGA  702
              X. alfalfae str. GEV-Rose-07 GGCACGCAGGCACTGCAGGAACGCTGGACGCCATG-CCCACCGTCGGCACCGGCGCATCCGCCTCGACCTACGCGGTGCGGCTGGCCGAAGATCTTTGGCAGGGCGAGCAAAATTGCGGA  702
                X. axonopodis str. Xac29-1 GGCACGCAGGCACTGCAGGAACGCTGGACGCCATG-CCCACCGTCGGCACCGGCGCATCCGCCTCAACCTACGCGGTGCGGCTGGCCGAAGATCTCTGGCAGGGCGAGCAACATTGCGGA  702
            X. citri subsp. citri str. jx4 GGCACGCAGGCACTGCAGGAACGCTGGACGCCATG-CCCACCGTCGGCACCGGCGCATCCGCCTCAACCTACGCGGTGCGGCTGGCCGAAGATCTCTGGCAGGGCGAGCAACATTGCGGA  702
     X. fuscans subsp. fuscans str. 4834-R GGCACGCCGGCACTGCAGGCACGCTGCACGCAATG-CCCACCGTCGGCACCGGCGCCTCCGCCTCGACCTACGCGGTGCGGCTGGCCGAAGATCTCTGGCAGGGCGGGCAACATTGCGGC  702
               X. vasicola str. NCPPB 1060 GACACGCAGGCACTGCAGGAACGCTGGACGCCATG-CCAACCGTCGGCACCGGCGCATCCGCCTCGACCTACGCGGTGCGGCTGGCCGAAGATCTTTGGCAGGGCGGGCAAAATTGCGGA  702
            X. oryzae pv. oryzicola BLS256 GGCACACAGGCACTGCAGGAATGCTGGACGCAATG-CCAACCGTCGGCAGCGGCGCATCCGCCTCGACCTACGCGGTTCGGCTGGCCGAAGATCTTTGGCAGGACGGGCAAAATTGCGGA  702
              X. campestris str. NCPPB4346 GGCACACAGGCAGTGCAGGAACGCTGGACGCAATG-CCAACCGTCGGCAGCGGCGCATCCGCCTCGACCTACGCGGTTCGGCTGGCCGAAGATCTTTGGCAGGACGGGCAAAATTGCGGA  702
                   X. bromi isolate LMG947 GGTACGCAGGCCCTGCAGAAACGCTGCACACCATG-CCAACCGTCGGCATAGGCGCGTCCGCCTCGACATACGCGGTTCGGCTGACCGAAGATCTCTGGCAGGGCGGAGAAAATTGCGGA  702
Acidovorax avenae subsp. avenae ATCC 19860 CCTGGGACGG-GCCGGACGCTGCCGGACCCGCATGGCAGCGCATCGGGGGCGGCGCGTCCGGCGAGGTATATGCCGTCCGGCTGGCAAGAAATTTCATGCAGGGCGGAGAGGACCATGGA  702
                                     ruler .......610.......620.......630.......640.......650.......660.......670.......680.......690.......700.......710.......720

                                           ** * ***  * ***** **  ** *        * **  **   *     **               *                **          *     * *     *  *   *
                X. fragariae isolate Fap21 CGGGACTTCGTCTTCAAAGCAATGTTGTGCCCGGATCCCCAAAAGCCGATTCCATGCGACTTGTATACCTCGCACCT---GCAGGATATATCAAATCCAAAGGAGGTGGTCTCGTTGGAA  825
                  X. gardneri str. JS749-3 CGGGACTTTGTCTTCAAGGCGATGTTGAACACAGACCCCAAAAATCCGATCCCGACCAACTTGTACGACAAGTACTTCCAGCAGGATGCGTCCGATCCGAAGGACCCTGTCTCGCTGCAC  828
               X. euvesicatoria str. 85-10 CGCGACTTCATCTTCAAGGCCTTGTTGCGCACCGACCCGCAACGGCCGATCCCGCCGACATTGTGCGACCCTGCCACCGCTGCAGACGCACCGGCGCTGCAGCAACGGGTCGCTGAGCGC  822
                  X. perforans str. 91-118 CGCGACTTCATCTTCAAGGCCTTGTTGCGCACCGACCCGCAACGGCCGATCCCGCCGACATTGTGCGACCCTGCCACCGCTGCAGACGCACCGGCGCTGCAGCAACGGATCGCTGCGCGC  822
              X. alfalfae str. GEV-Rose-07 CGCGACTTCATCTTCAAGGCCTTGTTGCGCACCGACCCGCAACGGCCGATCCCGCCGACATTGTGCGACCCTGCCACCGCTGCAGACGCACCGGCGCTGCAGCAACGGGTCGCTGAGCGC  822
                X. axonopodis str. Xac29-1 CGCGACTTCATCTTCAAGGCCTTGTTGTGCACGAACCCGCAACGGCCGATCCCACTGACACTGTACGACCCTGCCACCGTTGCAGACGCACCGACGCTGCAGGAACGGATCGCTGAGCGC  822
            X. citri subsp. citri str. jx4 CGCGACTTCATCTTCAAGGCCTTGTTGTGCACGAACCCGCAACGGCCGATCCCACTGACACTGTACGACCCTGCCACCGTTGCAGACGCACCGACGCTGCAGGAACGGATCGCTGAGCGC  822
     X. fuscans subsp. fuscans str. 4834-R CGCGACTTCATCTTCAAGGCCTTGTTGTGCACGGACCCGCAACGGCCGATCCCACCGACACTGTGCGACCCTGCCACCGTTGCAGACGCACCGACGCTGCAGGAGCGGATCGCCGAGCGC  822
               X. vasicola str. NCPPB 1060 CGTGACTTCATCTTCAAGGCCTTGTTGCGCACCGACCCGCAACCACCGACCCCACCGATACCGGGCGACCCCACTTCGGTTGCAGACGCACAGGCGCTGCAGCAACACATCGCTGCGCGC  822
            X. oryzae pv. oryzicola BLS256 CGCGCCTTCATCTTCAAGGCCTTGTTGCGCACCAACCCGCAACAGCCGGCCCCACCGACACTGGGCCACCCCACCTGCGTTGTAGACGCACAGGCGCTGCAGCAACGGATCGCTGCGCGC  822
              X. campestris str. NCPPB4346 CGCGCCTTCATCTTCAAGGCCTTGTTGCGCACCAACCCGCAACAGCCGGCCCCACCGACACTGGGCGACCCCACCTGCGTTGTAGACGCACAGGCGCTGCAGGAACGGATCGCTGCGCGC  822
                   X. bromi isolate LMG947 CGCGACTTCATCTTCAAGGCCTTGTTGTGCACAAATCCGCAACAGCCGATCCCACCGACCTTCGGCGGCGCTCACACCGTTGCAGACGCACCGGCGCTGCAGCAACGGATCGCTGCGCGC  822
Acidovorax avenae subsp. avenae ATCC 19860 CGAGACTTCGTGTTCAAGGCCATGCTCTCGCTCGATCCCGAAGACCGTCTGCCACCGCGGCTGCATG-CGCAGGCGCC--GCAGGATGGCGAGGGCCTCCGGCAAGCCATAGCGGCGCAC  819
                                     ruler .......730.......740.......750.......760.......770.......780.......790.......800.......810.......820.......830.......840

                                              *     **   *   ** *  **  *       *** *    *    * *     ** * *  *     **  * ** *******         * ** **  *    **    ***
                X. fragariae isolate Fap21 AAAGCCAAGATATACAAGGAATATCAGATGACGGTCTCGCTGGATGCAGGGTCTCGGGTCATGCGCGCGTACGGTCTAGTGCAGATCGATAATGTATTCGGAATACTACTGGAAAAGATC  945
                  X. gardneri str. JS749-3 GAAGCCAACATCTACAGGGAGTATCAGATGACCGTGTCGCTGGATGAGCGGTCTCGTATCATGCGTGCCTACGGCCTGGTGCAGATCGACGATGTATTCGGCATATTGCTTGAAAAAATC  948
               X. euvesicatoria str. 85-10 AAAGCCATGATCTTCCAGGAGTATCAAATGATCAGGTCGGTGGATGCAGTGCCGTATATCGTGCGCGCCCACGGCGTGGTGCAGATCGAACACACCTTCGGCATTTTGCTGGAGAAAATC  942
                  X. perforans str. 91-118 AAAGCCATGATCTTCCAGGAGTATCAAATGATCAGGTCGGTCGATGCAGTGCCGTATATCGTGCGCGCCCACGGCGTGGTGCAGATCGAACACACCTTCGGCATTTTGCTGGAGAAAATC  942
              X. alfalfae str. GEV-Rose-07 AAAGCCATGATCTTCCAGGAGTATCAAATGATCAGGTCGGTGGATGCAGTGCCGTATATCGTGCGCGCCCACGGCGTGGTGCAGATCGAACACACCTTCGGCATTTTGCTGGAGAAAATC  942
                X. axonopodis str. Xac29-1 AGAGCCATGATCTTCCAGGAGTATCAAATGATCAGGTCGGTGGATGCAGTGCCGTATATCGTCCACGCCCATGGCGTGGTGCAGATCGAACATACCTTCGGAATTTTGCTGGAGAAAATC  942
            X. citri subsp. citri str. jx4 AGAGCCATGATCTTCCAGGAGTATCAAATGATCAGGTCGGTGGATGCAGTGCCGTATATCGTCCACGCCCATGGCGTGGTGCAGATCGAACATACCTTCGGAATTTTGCTGGAGAAAATC  942
     X. fuscans subsp. fuscans str. 4834-R AAAGCCATGATCTTCCAGGAGTATCAAATGATCAGGTCGGTCGATGCGGTGCCGTATATCGTCCATGCCCATGGCGTGGTGCAGATCGAACACACCTTCGGAATTTTGCTGGAGAAAATC  942
               X. vasicola str. NCPPB 1060 AAAGCCATGATCTTCCAGGAGTATCAAATGATCAGGTCGGTGGATGCGGTGCCGTATATCGTCCGCGCCCACGGCGTGGTGCAGATCGAACACACCTTCGGAATTTTGCTGGAGAAAATC  942
            X. oryzae pv. oryzicola BLS256 AAGGCCATGATCTTCCAGGAATATCAAATGATCAGGTCGGTCGATGCGGTGCCCTATATCGTCCGCGCCCACGGCTTGGTGCAGATCGAACACACCTTCGGAATTTTGCTGGAAAGAATC  942
              X. campestris str. NCPPB4346 AAGGCCATGATCTTCCAGGAATATCAAATGATCAGGTCGGTCGATGCGGTGCCCTATATCGTCCGCGCCCACGGCGTGGTGCAGATCGAACACACATTCGGAATTTTGCTGGAAAAAATC  942
                   X. bromi isolate LMG947 AAGGCAATGATTTTCCAAGAGTATCAAATACTCAGGTCGGTAGATGCGGTGCCCTATATCGTCCGCGCCCATGGCGTGGTGCAGATCGAAGACGCATTCGGGATTCTGCTGGAGAAAATC  942
Acidovorax avenae subsp. avenae ATCC 19860 ACGGACCGCATCCACCAGGAGTTCCAGGTGGCCATCTCGCTGCGGGGAACGTCCCAGGTCATGCAGGTGCGCGGGCTCGTCCAGATCGGGAGCCGGCTGGGCATCCTGTCGGAGCGGATC  939
                                     ruler .......850.......860.......870.......880.......890.......900.......910.......920.......930.......940.......950.......960
```

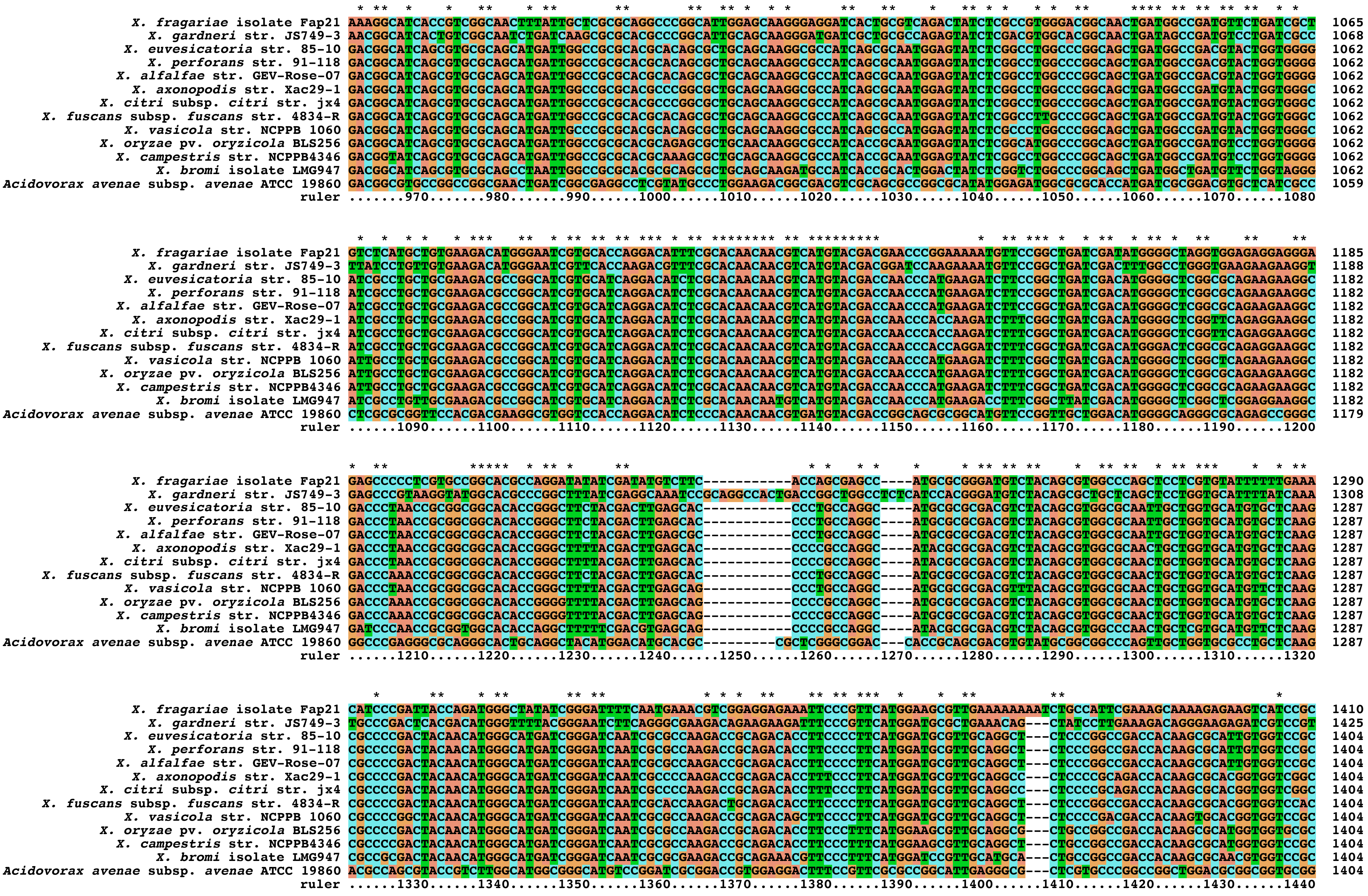

X. fragariae isolate Fap21 1065
X. gardneri str. JS749-3 1068
X. euvesicatoria str. 85-10 1062
X. perforans str. 91-118 1062
X. alfalfae str. GEV-Rose-07 1062
X. axonopodis str. Xac29-1 1062
X. citri subsp. citri str. jx4 1062
X. fuscans subsp. fuscans str. 4834-R 1062
X. vasicola str. NCPPB 1060 1062
X. oryzae pv. oryzicola BLS256 1062
X. campestris str. NCPPB4346 1062
X. bromi isolate LMG947 1062
Acidovorax avenae subsp. avenae ATCC 19860 1059
ruler 970 980 990 1000 1010 1020 1030 1040 1050 1060 1070 1080
X. fragariae isolate Fap21 1185
X. gardneri str. JS749-3 1188
X. euvesicatoria str. 85-10 1182
X. perforans str. 91-118 1182
X. alfalfae str. GEV-Rose-07 1182
X. axonopodis str. Xac29-1 1182
X. citri subsp. citri str. jx4 1182
X. fuscans subsp. fuscans str. 4834-R 1182
X. vasicola str. NCPPB 1060 1182
X. oryzae pv. oryzicola BLS256 1182
X. campestris str. NCPPB4346 1182
X. bromi isolate LMG947 1182
Acidovorax avenae subsp. avenae ATCC 19860 1179
ruler 1090 1100 1110 1120 1130 1140 1150 1160 1170 1180 1190 1200
X. fragariae isolate Fap21 1290
X. gardneri str. JS749-3 1308
X. euvesicatoria str. 85-10 1287
X. perforans str. 91-118 1287
X. alfalfae str. GEV-Rose-07 1287
X. axonopodis str. Xac29-1 1287
X. citri subsp. citri str. jx4 1287
X. fuscans subsp. fuscans str. 4834-R 1287
X. vasicola str. NCPPB 1060 1287
X. oryzae pv. oryzicola BLS256 1287
X. campestris str. NCPPB4346 1287
X. bromi isolate LMG947 1287
Acidovorax avenae subsp. avenae ATCC 19860 1287
ruler 1210 1220 1230 1240 1250 1260 1270 1280 1290 1300 1310 1320
X. fragariae isolate Fap21 1410
X. gardneri str. JS749-3 1425
X. euvesicatoria str. 85-10 1404
X. perforans str. 91-118 1404
X. alfalfae str. GEV-Rose-07 1404
X. axonopodis str. Xac29-1 1404
X. citri subsp. citri str. jx4 1404
X. fuscans subsp. fuscans str. 4834-R 1404
X. vasicola str. NCPPB 1060 1404
X. oryzae pv. oryzicola BLS256 1404
X. campestris str. NCPPB4346 1404
X. bromi isolate LMG947 1404
Acidovorax avenae subsp. avenae ATCC 19860 1404
ruler 1330 1340 1350 1360 1370 1380 1390 1400 1410 1420 1430 1440

```
                                                    **  ****  ***** *   *                ** *** **     *****     * * **  *            * *       **          *     **
         X. fragariae isolate Fap21                 TTTCTCAATCGCATGATTTCCATAGATGCCAATGGTCGTGCCAGGGCGGAGGATTTGCTGCTGGACTCGTTTATCAATGACCCGACGATTCCGCTACGGAATCATGTCCATGCAACCTAT  1530
         X. gardneri str. JS749-3                   TTTTTCAACCGCATGATTACCCCAAAGGAGAACGGCGGCGCCACGGCAGAGGATTTGCTGAAGGACCCGTTCCTCAACGCCGCCGCGATAGGGCCCCGGGACAATGTCCATGCAACTCTC  1545
         X. euvesicatoria str. 85-10                TTCTTCAACAGCATGCTGGACGACGGAACCGGCGAGCGCACCAAGGCAGAGAGACTGCTGCGCGACCCCTTTTTCACCGAACCGCCGCTCCCGCCCCGTGATCGGATGCACCGCACCTAC  1524
         X. perforans str. 91-118                   TTCTTCAACAGCATGCTGGACGACGGAACCGGCGAGCGCACCAAGGCAGAGAGACTGCTGCGCGACCCCTTTTTCACCGAACCGCCGCTCCCGCCCCGTGATCGGATGCACCGCACCTAC  1524
         X. alfalfae str. GEV-Rose-07               TTCTTCAACAGCATGCTGGACGACGGAACCGGCGAGCGCACCAAGGCAGAGAGACTGCTGCGCGACCCATTTTTTACCGAACCGCCGCTCCCGCCCCGTGATCGGATGCACCGCACCTAC  1524
         X. axonopodis str. Xac29-1                 TTCTTCAACAGCATGCTGGACGACGGAACCGGCGAACGCACCAAGGCCGAGAGACTGCTGCGCGCCCCCTTTTTCCCCGAACCGCCGCTCCCGCCCCGTGATCGGATGCATCGCACCTTC  1524
         X. citri subsp. citri str. jx4             TTCTTCAACAGCATGCTGGACGACGGAACCGGCGAACGCACCAAGGCCGAGAGACTGCTGCGCGCCCCCTTTTTCACCGAACCGCCGCTCCCGCCCCGTGATCGGATGCATCGCACCTTC  1524
         X. fuscans subsp. fuscans str. 4834-R      TTCTTCAACAGCATGCTGGACGACGGAACCGGCGAGCGCACCAAGGCCGAGATCCTGCTGCGCGACCCCTTTTTCACCGAACCGCCGCTCCCTCCCCGTGATCGGATGCATCGCACCTAT  1524
         X. vasicola str. NCPPB 1060                TTCTTCAACAGCATGCTGGACGATGGAACCGGCCAGCGCACCAAGGCGGAGACCTTGCTGCGCAACCCCTTTTTCACCCAACAGCCGCTCCCGCCCCGTGATCGGATGCATCGCACCTAC  1524
         X. oryzae pv. oryzicola BLS256             TTCTTCAACAGCATGCTGGACGATGGAACCGGCGAGCGCACCAAGGCCGAGGTCTTGCTGCGCGCCCCCTTTTTCACCGAAACGCCGCTTCCGCCCCGTGCTCGGATGCATCGCACCTAC  1524
         X. campestris str. NCPPB4346               TTCTTCAACAGCATGCTGGACGATGGAACCGGCGAGCGCACCAAGGCCGAGGTCTTGCTGCGCGACCCCTTTTTCACCGAAACACCGCTTCCGCCGCGTGCTCGGATGCATCGCACCTAC  1524
         X. bromi isolate LMG947                    TTCTTCAACCGCATGCTGAACGATGGAACGGGCAAGCGCACCAAGGCCGAAATCTTGCTGCGCGACCCCTTTTTCACCGAAACGCCGCTCCCGACCCGGGACCGGATTCATCGCACCTAC  1524
         Acidovorax avenae subsp. avenae ATCC 19860 TTCATCAACCGCATGATCGGCCGGCAACCGGACGCCCGCAGCACGGCCGAGGAGCTGCTGGGCGACCCGTTCCTGCAGGA-------GCTGGAGCCCCGCGCATTGACGCGTGCCACGGTG  1518
         ruler                                      ......1450......1460......1470......1480......1490......1500......1510......1520......1530......1540......1550......1560

                                                     * **   * *
         X. fragariae isolate Fap21                 AAAAA---ACTGGCTTGA------------------  1545
         X. gardneri str. JS749-3                   GAAAA---ATTGACCTGA------------------  1560
         X. euvesicatoria str. 85-10                GAAAA---ACTGAAGCGCTTGCCTTCTTTTTAG---  1554
         X. perforans str. 91-118                   GAAAA---ACTGAAGCGTTTGCCTTCTTTTTAG---  1554
         X. alfalfae str. GEV-Rose-07               GAAAA---ACTGAAACGCCTGCCTTCTTTTTAG---  1554
         X. axonopodis str. Xac29-1                 GAAAA---ACTGACGCGCTTGCCTTCTTTTTAG---  1554
         X. citri subsp. citri str. jx4             GAAAA---ACTGACGCGCTTGCCTTCTTTTTAG---  1554
         X. fuscans subsp. fuscans str. 4834-R      GAAAA---ACTGAAGCGCTTGCCTTCTTTTTAG---  1554
         X. vasicola str. NCPPB 1060                GAAAA---ACTAAGGCGCTTGCCTTCTTTTTAG---  1554
         X. oryzae pv. oryzicola BLS256             GAAAA---ACTGAAGCGCGTGCCTTCTTTTTAG---  1554
         X. campestris str. NCPPB4346               GAAAA---ACTGAAGCGCGTGCCTTCTTTTTAG---  1554
         X. bromi isolate LMG947                    GAAAA---ATTGAAGCGCTTGCCTTCTTTTTAG---  1554
         Acidovorax avenae subsp. avenae ATCC 19860 GAGAAGGTACTGAACCCCGAAACGCCGCCGGGATGA  1554
         ruler                                      ......1570......1580......1590......
```
