# Supplementary figures and images for: The Xanthomonas euvesicatoria type III effector XopAU is an active protein kinase that manipulates plant MAP kinase signaling
Source: PLoS Pathog. 2018 Jan 29;14(1):e1006880. doi: 10.1371/journal.ppat.1006880 (PMC5805367; doi:10.1371/journal.ppat.1006880)

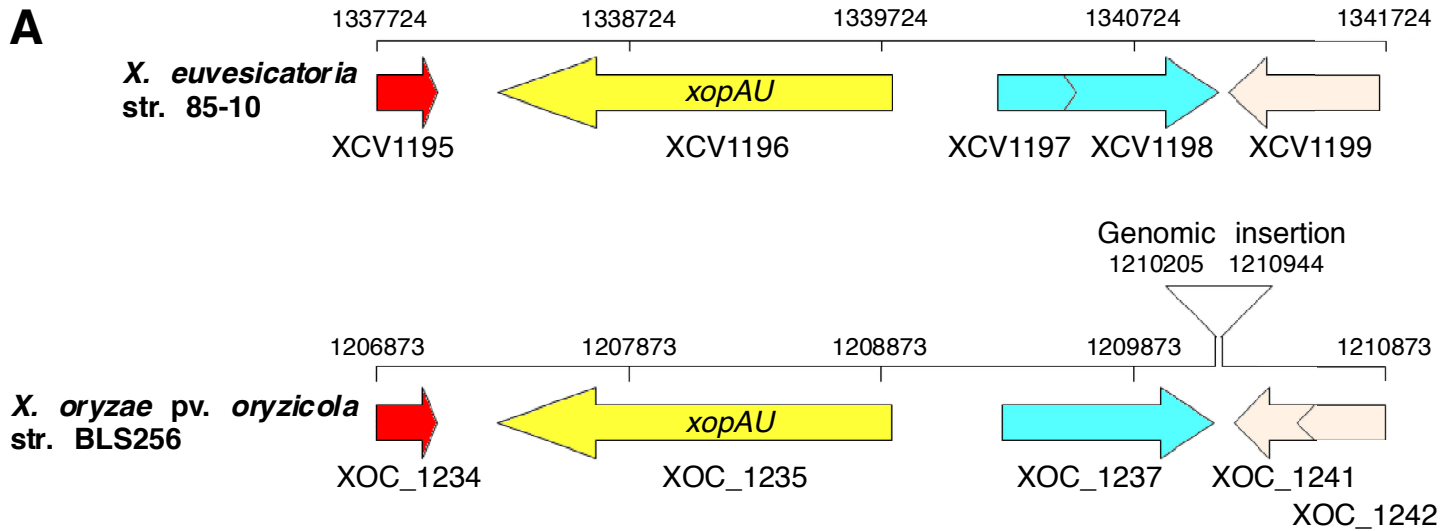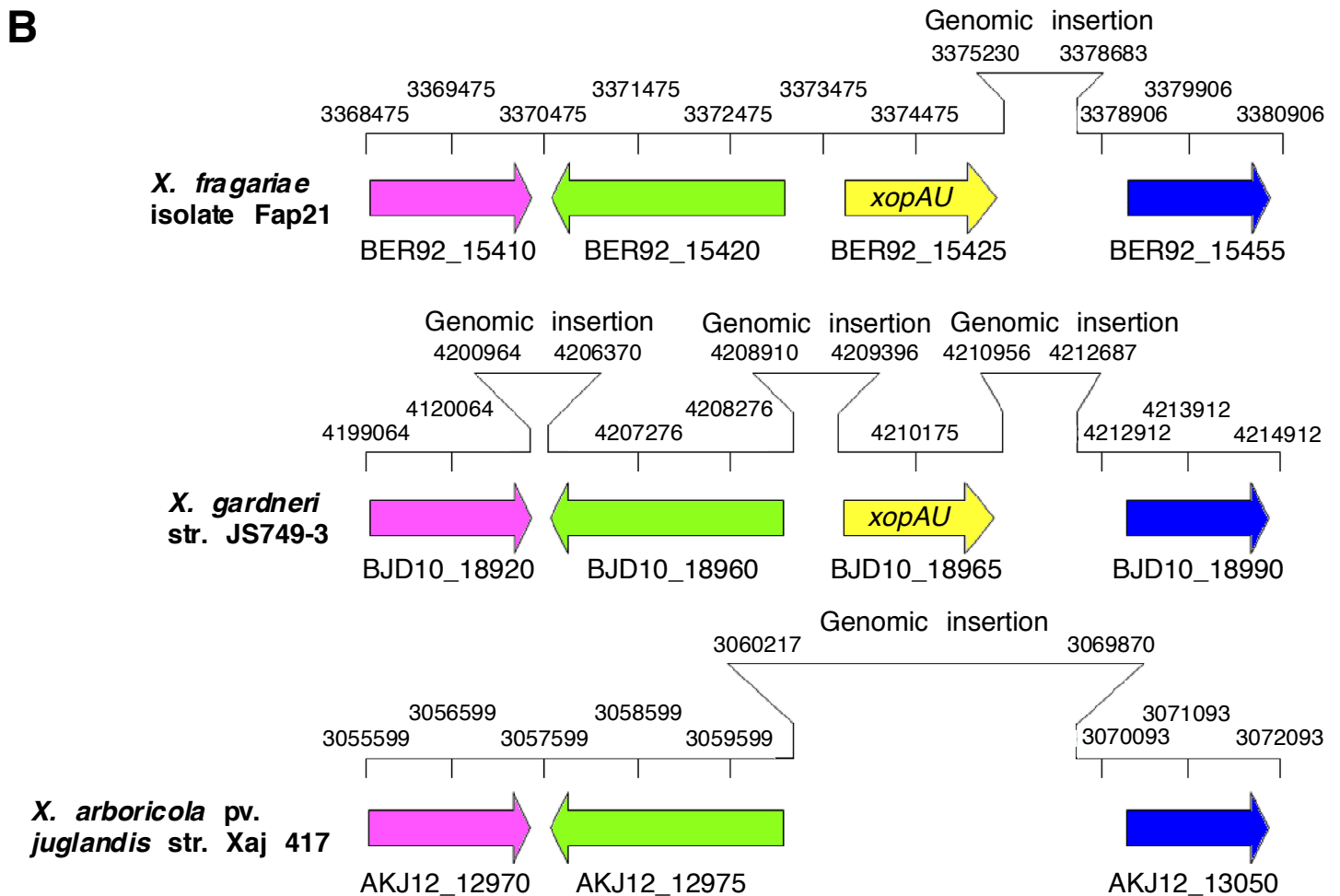

Supplement: S2 Fig — (A) Genomic location of the xopAU allelic variant of group 1 Xanthomonas strains in X. euvesicatoria (acc. num. NC_007508.1) and X. oryzae (acc. num. CP003057.2). (B) Genomic location of the xopAU group 2 allelic variant in X. fragariae (acc. num. CP016830.1) and X. gardneri (acc. num. CP018728.1), and corresponding genomic region in the X. arboricola strain (acc. num. CP012251.1), which does not contain the xopAU allele. Numbers and arrows represent genomic location and open reading frames (ORF), respectively. Locus tags are indicated below each ORF, which are colored based on DNA sequence homology. (PDF) [file ppat.1006880.s006.pdf]

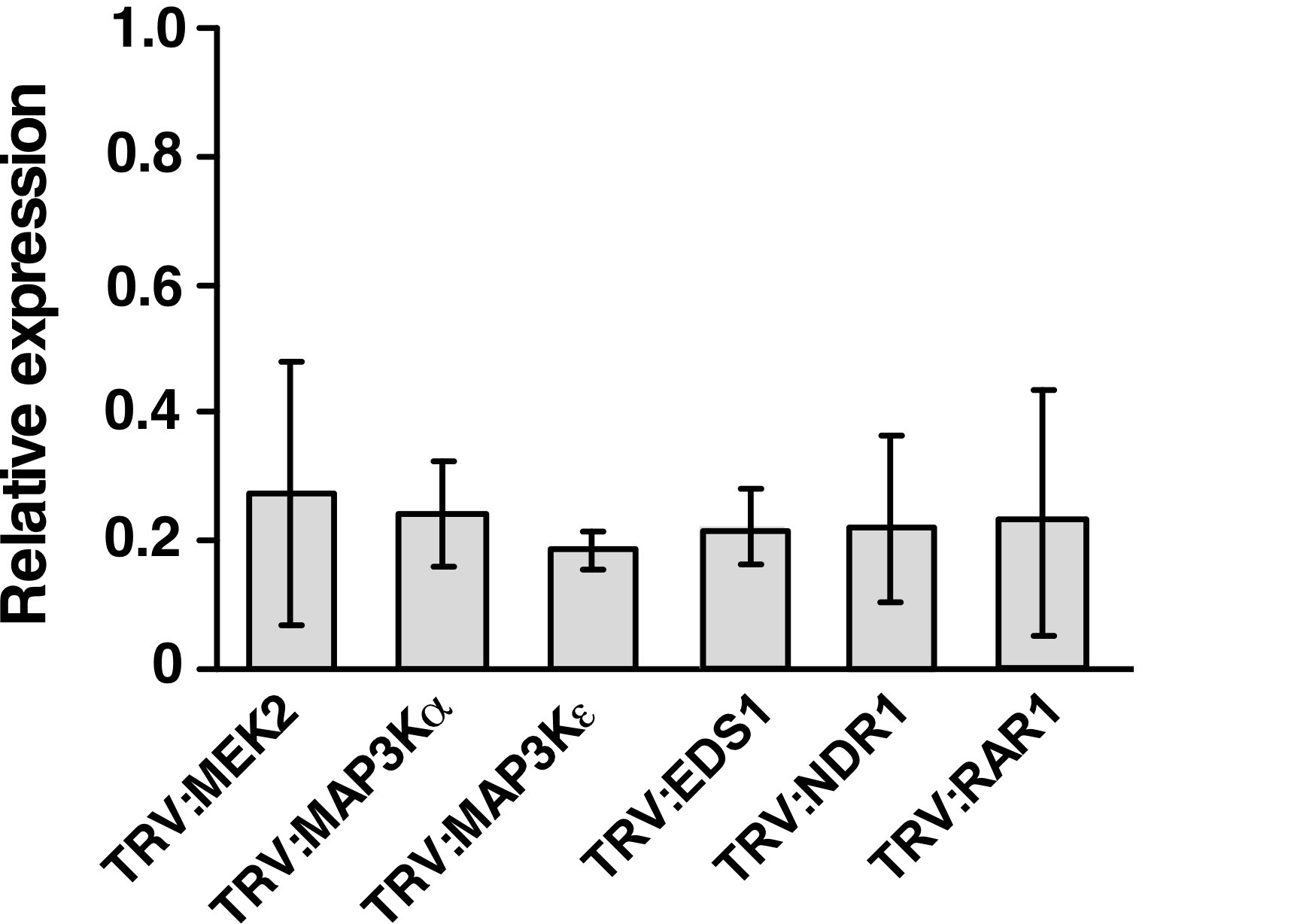

Supplement: S4 Fig — N. benthamiana plants were infected with TRV, TRV:MEK2, TRV:MAP3Kα, TRV:MAP3Kε, TRV:EDS1, TRV:NDR1, and TRV:RAR1. Four weeks after infection, qRT-PCR was used to assess the expression of the targeted gene in the silenced plants relative to plants infected with empty TRV. Values are means ± SE of three biological repeats. (TIF) [file ppat.1006880.s008.tif]

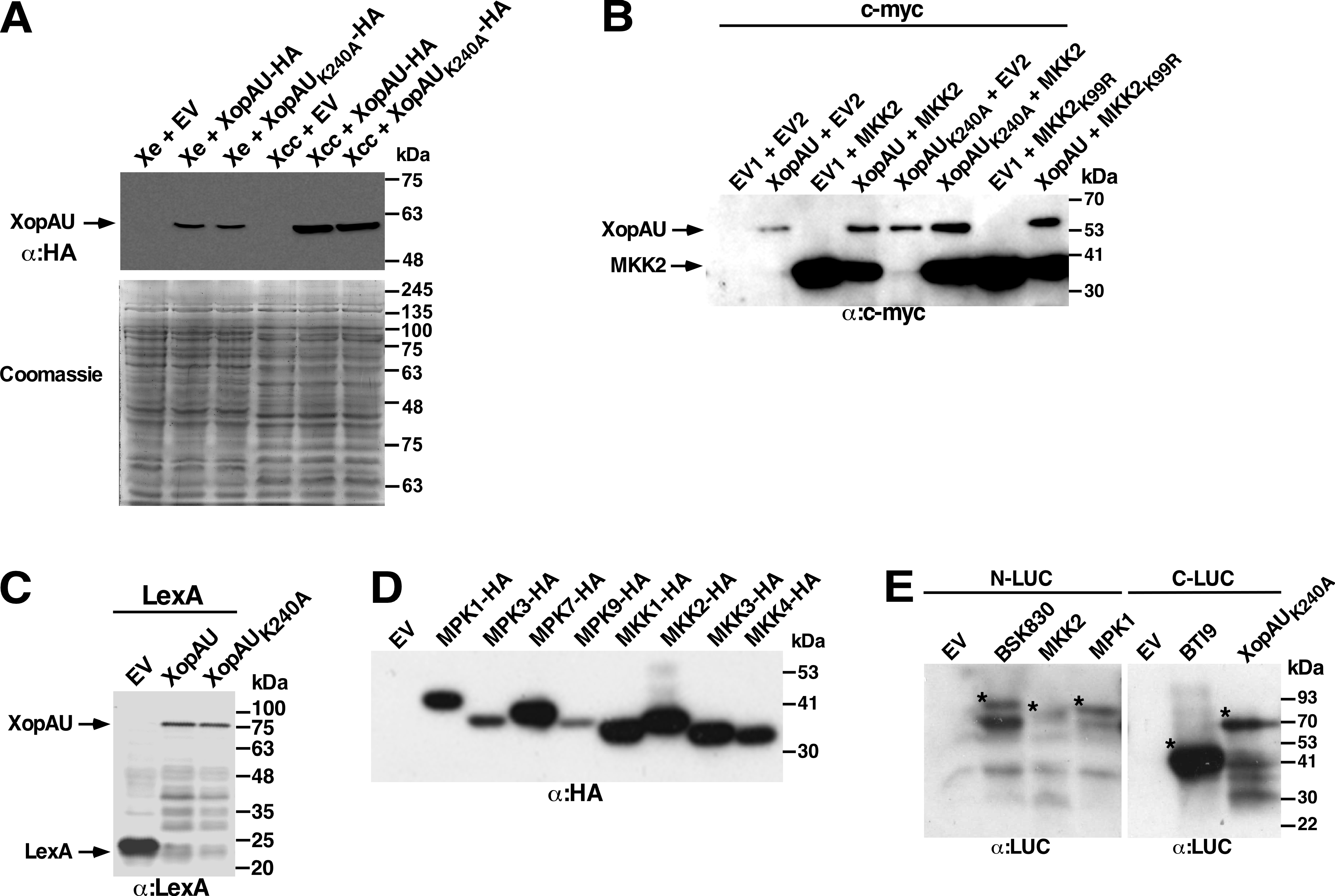

Supplement: S6 Fig — Total protein was extracted from Xe or Xcc bacteria (A), yeast (B, C and D), and N. benthamiana plants (E), separated by SDS-PAGE and immunoblotted with the indicated antibodies. In (E) asterisks indicate bands corresponding to the full-length proteins. (TIF) [file ppat.1006880.s010.tif]

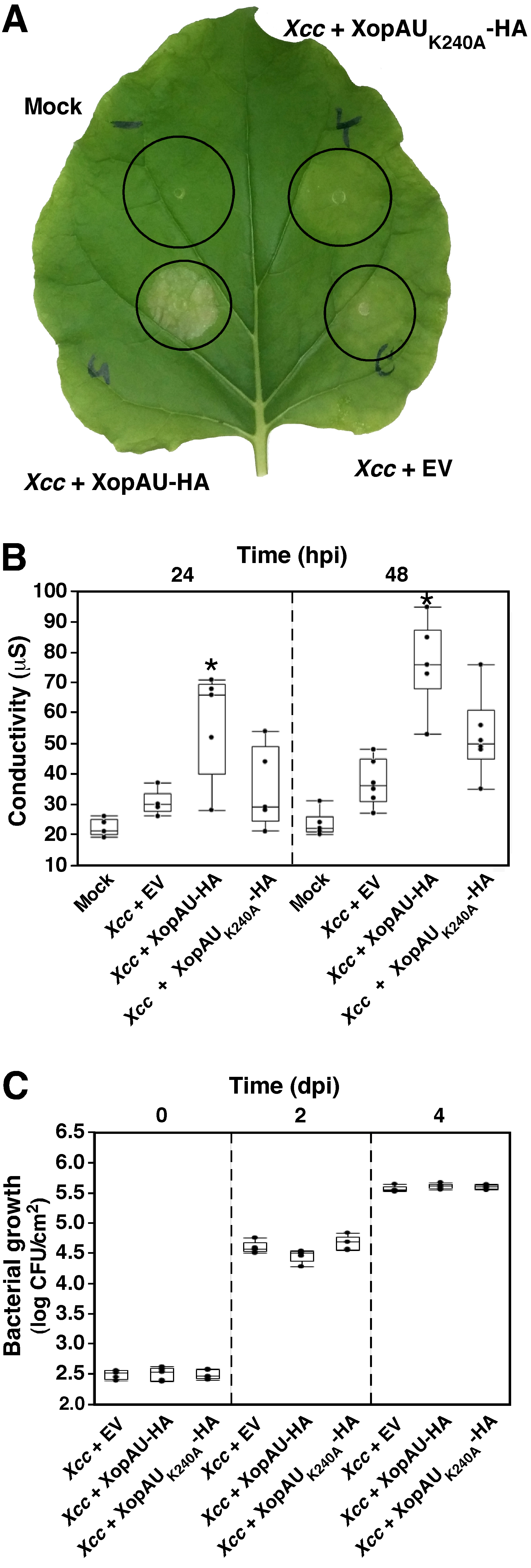

Supplement: S7 Fig — N. benthamiana leaves were syringe-infiltrated with a 10 mM MgCl2 mock solution (Mock) or with suspensions (5 x 10^7^ CFU/ml) of Xcc strains containing a vector for expression of XopAU-HA and XopAUK240A-HA, or an empty vector (EV). (A) Photograph of an inoculated leaf at two days post-inoculation (dpi). Electrolyte leakage (B) and bacterial growth (C) in the inoculated areas was quantified at the indicated hours (hpi) and days post-inoculation (dpi), respectively. The box plots display 25th, 50th (middle line) and 75th percentiles (n = 5). An asterisk indicates a significant difference (Mann-Whitney U test, p value <0.05) compared to Xcc containing an EV. (TIF) [file ppat.1006880.s011.tif]

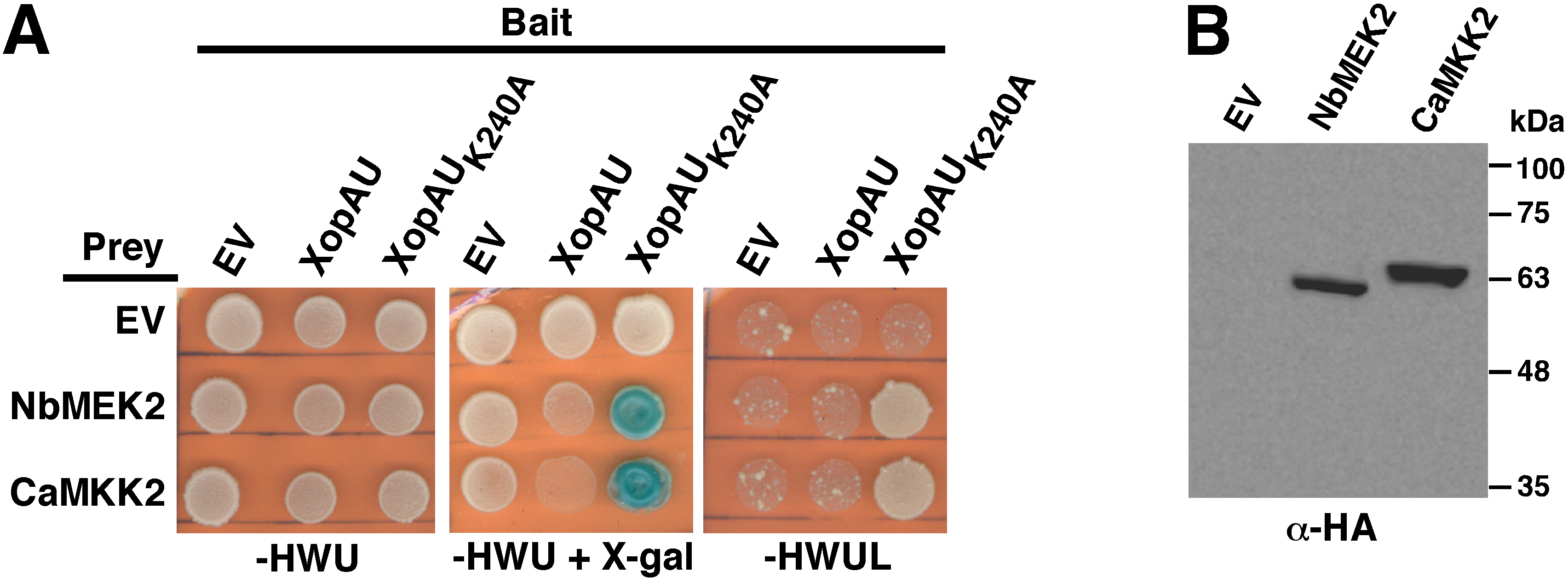

Supplement: S8 Fig — (A) Yeast expressing the indicated combinations of bait and prey were spotted on either selective medium (-HWUL) or non-selective medium (-HWU) with or without the addition of X-gal. (B) Western blot analysis to assess expression of NbMEK2 and CaMKK2 in yeast. Total protein was extracted from yeast, separated by SDS-PAGE and immunoblotted with α:HA antibodies. (TIF) [file ppat.1006880.s012.tif]
