# Supplementary material for: The Xanthomonas euvesicatoria type III effector XopAU is an active protein kinase that manipulates plant MAP kinase signaling
Source: PLoS Pathog. 2018 Jan 29;14(1):e1006880. doi: 10.1371/journal.ppat.1006880 (PMC5805367; doi:10.1371/journal.ppat.1006880)
Supplement: S3 Fig — Protein sequences of the XopAU homologs (S1 Table) were aligned with the COBALT multiple sequence alignment tool (https://www.ncbi.nlm.nih.gov/tools/cobalt/cobalt.cgi?) using default parameters. Blue fonts represent identical amino acids; yellow fonts represent nearly invariant residues in the protein kinase superfamily [38]. Roman numerals above the sequence indicate conserved kinase subdomains [38]. (PDF) [file ppat.1006880.s007.pdf]

X. euvesicatoria str. 85-10	MKQISGNFTLPSTALRA--DAERDA-PETAQAP--EPHTP-PHPVADHPL	44
Acidovorax avenae subsp. *avenae* ATCC 19860	---MAGLPPLSIPSVRPARDGEGTANPEAAPTG--PPRPPGPHAVAHVPL	45
X. alfalfae str. GEV-Rose-07	MKQISGNFTLPSTALRA--DAERDA-PETAQAP--EPHTP-PHPVADHPL	44
X. axonopodis str. Xac29-1	MKQISGNFTLPSTALRA--DAERDA-PETAQAP--EQHTP-PHPVADHPL	44
X. bromi isolate LMG947	MKHISGKPTLLSTAMHA--DAERDE-AETAYSP--EQDTL-LHPVADHPL	44
X. campestris str. NCPPB4346	MKQISGKFTPPSTALRA--DAERDA-PDTAQAP--EQHPP-PHPVADHPL	44
X. citri subsp. *citri* str. jx4	MKQISGNFTLPSTALRA--DAERDA-PETAQAP--EQHTP-PHPVADHPL	44
X. fragariae isolate Fap21	MKKISDTSPAFRIGMQA--EAEQVQ-TSMDRVP--EHQHV-AEPAQDHPL	44
X. fuscans subsp. *fuscans* str. 4834-R	MKQISGNVTLPSTALRA--DAERDA-PETAQAP--EQHTP-PHPVADHPL	44
X. gardneri str. JS749-3	MKKISPTSPVIPHGMQA----EGDQ-SETSMHPASEHHEPAATPGQDPLL	45
X. oryzae pv. *oryzicola* BLS256	MKQISGKFTPPSTALRA--DAKRDA-PDTAQVP--EQHPP-PHPVADHPL	44
X. perforans str. 91-118	MKQISGNFTLPSTALRA--DAERDA-PETAQAP--EPHTP-PHPVADHPL	44
X. vasicola str. NCPPB 1060	MKQISGNFTLPSTALRA--DAERDA-PETAQAT--EQHTP-PHPVADHPL	44
ruler	1.......10........20........30........40........50	

X. euvesicatoria str. 85-10	NLLEKRRSITGVLADERKRLEV--VVPRLQAALAP--HTWSSSSSDSPTT	90
Acidovorax avenae subsp. *avenae* ATCC 19860	LARRRGQSGEGPSAERAPRLAPLDALRRASTFVLPR----SPSSFDHGRR	91
X. alfalfae str. GEV-Rose-07	NLLEKRRSITGVLADERKRLEV--VVPRLQAALAP--HTWSSSSSDSPTT	90
X. axonopodis str. Xac29-1	NLLEKRRSTTGMLADERKRLEV--VVPHLQAALAP--HTWSSSSSDSPTT	90
X. bromi isolate LMG947	NLLEKRRSTTGVLADEHSQLQV--VVPRLQASLAP--QTWSSSSSGSPTT	90
X. campestris str. NCPPB4346	NLLEKRRTTIGLLADERKRLEV--VVPRLQPSLAP--QTWSSSSSDSPAT	90
X. citri subsp. *citri* str. jx4	NLLEKRRSTTGMLADERKRLEV--VVPHLQAALAP--HTWSSSSSDSPTT	90
X. fragariae isolate Fap21	NLLEKRLRKTAESSSEDPSLHI--SVPTISGRLNRFISRSFSSSSNAPTT	92
X. fuscans subsp. *fuscans* str. 4834-R	NLLEKRRSTTGVLADENTRLEV--VVPRLQAALAP--HTWSSSSSDSPTT	90
X. gardneri str. JS749-3	NLLEKRRIRSGESSVRELSVDIDRPPPRL---IVPRCLPRSLSSGSTSTT	92
X. oryzae pv. *oryzicola* BLS256	NLLEKRRTTIGLLADERKRLEV--VVPRLQPCLAP--QTWSSSSSDSPTT	90
X. perforans str. 91-118	NLLEKRRSITGVLADERKRLEV--VVPRLQAALAP--HTWSSSSSDSPTT	90
X. vasicola str. NCPPB 1060	NLLEKRRSTTGVLADERTHLEV--VVPRLQAALAP--HTWSSSSSDSPTT	90
ruler	........60........70........80........90.........100	

X. euvesicatoria str. 85-10	PVTGR-SPRLGPQPGSP-TWACLLETPARLSALAGADSSDAFQNLRNARR	138
Acidovorax avenae subsp. *avenae* ATCC 19860	PGAGRASPSHGAGSSQPPSLASLLDPSTPIARERDGDCWSPVRALRRERR	141
X. alfalfae str. GEV-Rose-07	PVTGR-SPRLGPQPGSP-TWACLLETPARLSALAGADSSDAFQNLRNARR	138
X. axonopodis str. Xac29-1	PVTGR-SPRLGQQPGSP-TWACLLETPARTSALAGADSNDAFQNLRNARR	138
X. bromi isolate LMG947	PVTGR-SPRSGARPGSP-ILASLLETPARASATVGADSSNAFQNLRNARR	138
X. campestris str. NCPPB4346	PLSSR-SPRLGTQPGLP-TWACLLETPTRTSALAGADSSDAFQNLRNARR	138
X. citri subsp. *citri* str. jx4	PVTGR-SPRLGQQPGSP-TWACLLETPARTSALAGADSNDAFQNLRNARR	138
X. fragariae isolate Fap21	PGSGF-SGRPTGYPGFP-TLASLLDPPTPRSHSPPSGLSHASDILRNERQ	140
X. fuscans subsp. *fuscans* str. 4834-R	PVTGR-SPRLGPQPGSP-TWACLLETPARTSALAGADSNDAFQNLRNARR	138
X. gardneri str. JS749-3	PGSDS-SRRSTPYSDLP-VLGSLLDPPTPGSLALTPPPSNAFGILRNERM	140
X. oryzae pv. *oryzicola* BLS256	PLSSR-SPRLGTQPGLP-TWACLLETPTRTSALAGTDSSDAFQNLRNARQ	138
X. perforans str. 91-118	PVTGR-SPRLGPQPGSP-TWACLLETPARLSALAGADSSDAFQNLRNARR	138
X. vasicola str. NCPPB 1060	SATGR-SPRLSPQPGSP-TWACLLETPAHTSALAGADSSDAFQNLRNARQ	138
ruler	.......110.......120.......130.......140.......150	
X. euvesicatoria str. 85-10	LVSDTRHARQHVVLSPRLVKSLTSRSERPDLIQKLSALKPGALDVDNGFL	188
Acidovorax avenae subsp. *avenae* ATCC 19860	MQAELRASSGAGVLSPHVVHSMGSP---PGWEARLAALKPGAAEVQQGFE	188
X. alfalfae str. GEV-Rose-07	LVSDTRHARQHVVLSPRLVKSLTSRSERPDLIQKLSALKPGALDVDNGFL	188
X. axonopodis str. Xac29-1	LVSEMRHARQHVVLSPRLVKSLTSRSERPDLIQKLSALKPGALDVDTGFL	188
X. bromi isolate LMG947	RVSDMRHARQHVVLSPRLVNSLTSRSERPDLILKLSALKPGALDVDGGFL	188
X. campestris str. NCPPB4346	LVSDMRHARQHVMLSPRLVKSLTSRSERPDLIQKLSALKPGALDVDSGFV	188
X. citri subsp. *citri* str. jx4	LVSEMRHARQHVVLSPRLVKSLTSRSERPDLIQKLSALKPGALDVDTGFL	188
X. fragariae isolate Fap21	RTRDLRYVQQDVVLSPRTVRSLAAKSQRPEVIERLSTLKPGALEIENGFL	190
X. fuscans subsp. *fuscans* str. 4834-R	LVSEMRHARQHVVLSPRLVKSLTSRSERPDLIQKLSALKPGALDVDTGFL	188
X. gardneri str. JS749-3	RMRETRYRRQDVVLSPRTLKDLASNSPRPELLTKLSLLKPGALEIENGFL	190
X. oryzae pv. *oryzicola* BLS256	LVSDMRHARQHVVLSPRLVKSLTSRSERPDLIQKLSALKPGALDVDSGFV	188
X. perforans str. 91-118	LVSDTRHARQHVVLSPRLVKSLTSRSERPDLIQKLSALKPGALDVDNGFL	188
X. vasicola str. NCPPB 1060	LVSDMRRARQHVVLSPRLVKSLTSRSERPDLIQKLSALKPGASDVDGGFL	188
ruler	.......160.......170.......180.......190.......200	

X. euvesicatoria str. 85-10	HVTLETGHAGTAGTLDAMPTVGTGASASTYAVRLAEDLWQGGQNCGRDFI	238
Acidovorax avenae subsp. *avenae* ATCC 19860	RVDLDTAWDGPDAAGPAWQRIGGGASGEVYAVRLARNFMQGGEDHGRDFV	238
X. alfalfae str. GEV-Rose-07	HVTLETGHAGTAGTLDAMPTVGTGASASTYAVRLAEDLWQGEQNCGRDFI	238
X. axonopodis str. Xac29-1	KVTLETGHAGTAGTLDAMPTVGTGASASTYAVRLAEDLWQGEQHCGRDFI	238
X. bromi isolate LMG947	QVTLGTRYAGPAETLHTMPTVGIGASASTYAVRLTEDLWQGGENCGRDFI	238
X. campestris str. NCPPB4346	RVTLETGHTGSAGTLDAMPTVGSGASASTYAVRLAEDLWQDGQNCGRAFI	238
X. citri subsp. *citri* str. jx4	KVTLETGHAGTAGTLDAMPTVGTGASASTYAVRLAEDLWQGEQHCGRDFI	238
X. fragariae isolate Fap21	PVRLERAHDSDPRTLQGMRPIGSGASGSAYAVRLAEDFWRAGENCGRDFV	240
X. fuscans subsp. *fuscans* str. 4834-R	QVTLETGHAGTAGTLHAMPTVGTGASASTYAVRLAEDLWQGGQHCGRDFI	238
X. gardneri str. JS749-3	PVRLEGTHNGDTQNLKDMQPLGKGASGRAYAVRLAEDFWRGEENCGRDFV	240
X. oryzae pv. *oryzicola* BLS256	RVTLETGHTGTAGMLDAMPTVGSGASASTYAVRLAEDLWQDGQNCGRAFI	238
X. perforans str. 91-118	HVTLETGHAGTAGTLDAMPTVGTGASASTYAVRLAEDLWQGGQNCGRDFI	238
X. vasicola str. NCPPB 1060	HVTLETGHAGTAGTLDAMPTVGTGASASTYAVRLAEDLWQGGQNCGRDFI	238
ruler	.......210.......220.......230.......240.......250	

X. euvesicatoria str. 85-10	FKALLRTDPQRPIPPTLCDPATAADAPALQQRVAERKAMIFQEYQMIRSV	288
Acidovorax avenae subsp. *avenae* ATCC 19860	FKAMLSLDPEDRLPPRLHAQAP-QDGEGLRQAIAAHTDRIHQEFQVAISL	287
X. alfalfae str. GEV-Rose-07	FKALLRTDPQRPIPPTLCDPATAADAPALQQRVAERKAMIFQEYQMIRSV	288
X. axonopodis str. Xac29-1	FKALLCTNPQRPIPLTLYDPATVADAPTLQERIAERRAMIFQEYQMIRSV	288
X. bromi isolate LMG947	FKALLCTNPQQPIPPTFGGAHTVADAPALQQRIAARKAMIFQEYQILRSV	288
X. campestris str. NCPPB4346	FKALLRTNPQQPAPPTLGDPTCVVDAQALQERIAARKAMIFQEYQMIRSV	288
X. citri subsp. *citri* str. jx4	FKALLCTNPQRPIPLTLYDPATVADAPTLQERIAERRAMIFQEYQMIRSV	288
X. fragariae isolate Fap21	FKAMLCPDPQKPIPCDLYTSH-LQDISNPKEVVSLEKAKIYKEYQMTVSL	289
X. fuscans subsp. *fuscans* str. 4834-R	FKALLCTDPQRPIPPTLCDPATVADAPTLQERIAERKAMIFQEYQMIRSV	288
X. gardneri str. JS749-3	FKAMLNTDPKNPIPTNLYDKYFQQDASDPKDPVSLHEANIYREYQMTVSL	290
X. oryzae pv. *oryzicola* BLS256	FKALLRTNPQQPAPPTLGHPTCVVDAQALQQRIAARKAMIFQEYQMIRSV	288
X. perforans str. 91-118	FKALLRTDPQRPIPPTLCDPATAADAPALQQRIAARKAMIFQEYQMIRSV	288
X. vasicola str. NCPPB 1060	FKALLRTDPQPPTPPIPGDPTSVADAQALQQHIAARKAMIFQEYQMIRSV	288
ruler	.......260.......270.......280.......290.......300	

IV V

X. euvesicatoria str. 85-10	DAVPYIVRAHGVVQIEHTFGILLEKIDGISVRSMIGRARTALQQGAISAM	338
Acidovorax avenae subsp. *avenae* ATCC 19860	RGTSQVMQVRGLVQIGSRLGILSERIDGVPAGELIGEASYALEDGDVAAP	337
X. alfalfae str. GEV-Rose-07	DAVPYIVRAHGVVQIEHTFGILLEKIDGISVRSMIGRARTALQQGAISAM	338
X. axonopodis str. Xac29-1	DAVPYIVHAHGVVQIEHTFGILLEKIDGISVRSMIGRARPALQQGAISAM	338
X. bromi isolate LMG947	DAVPYIVRAHGVVQIEDAFGILLEKIDGISVRSLIGRARAALQQDAITAL	338
X. campestris str. NCPPB4346	DAVPYIVRAHGVVQIEHTFGILLEKIDGISVRSMIGRARKALQQGAITAM	338
X. citri subsp. *citri* str. jx4	DAVPYIVHAHGVVQIEHTFGILLEKIDGISVRSMIGRARPALQQGAISAM	338
X. fragariae isolate Fap21	DAGSRVMRAYGLVQIDNVFGILLEKIKGITVGNFIARAGPALEQGRITAS	339
X. fuscans subsp. *fuscans* str. 4834-R	DAVPYIVHAHGVVQIEHTFGILLEKIDGISVRSMIGRARTALQQGAISAM	338
X. gardneri str. JS749-3	DERSRIMRAYGLVQIDDVFGILLEKINGITVGNLIKRARPALQQGMIAAP	340
X. oryzae pv. *oryzicola* BLS256	DAVPYIVRAHGLVQIEHTFGILLERIDGISVRSMIGRARRALQQGAITAM	338
X. perforans str. 91-118	DAVPYIVRAHGVVQIEHTFGILLEKIDGISVRSMIGRARTALQQGAISAM	338
X. vasicola str. NCPPB 1060	DAVPYIVRAHGVVQIEHTFGILLEKIDGISVRSMIARARTALQQGAISAM	338
ruler	.......310.......320.......330.......340.......350	

VI VII

X. euvesicatoria str. 85-10	EYLGLARQLMADVLVGIACCEDAGIVHQDISHNNVMYDQPMKIFRLIDMG	388
Acidovorax avenae subsp. *avenae* ATCC 19860	AHMEMARTMIADVLIALARFHDEGVVHQDISHNNVMYDRQRGMFRLLDMG	387
X. alfalfae str. GEV-Rose-07	EYLGLARQLMADVLVGIACCEDAGIVHQDISHNNVMYDQPMKIFRLIDMG	388
X. axonopodis str. Xac29-1	EYLGLARQLMADVLVGIACCEDAGIVHQDISHNNVMYDQPTKIFRLIDMG	388
X. bromi isolate LMG947	DYLGLARQLMADVLVGIACCEDAGIVHQDISHNNVMYDQPMKTFRLIDMG	388
X. campestris str. NCPPB4346	EYLGLARQLMADVLVGIACCEDAGIVHQDISHNNVMYDQPMKIFRLIDMG	388
X. citri subsp. *citri* str. jx4	EYLGLARQLMADVLVGIACCEDAGIVHQDISHNNVMYDQPTKIFRLIDMG	388
X. fragariae isolate Fap21	DYLAVGRQLMADVLIAVSCCEDMGIVHQDISHNNVMYDEPGKMFRLIDMG	389
X. fuscans subsp. *fuscans* str. 4834-R	EYLGLARQLMADVLVGIACCEDAGIVHQDISHNNVMYDQPTRIFRLIDMG	388
X. gardneri str. JS749-3	EYLDVARQLIADVLIALSCCEDMGIVHQDVSHNNVMYDGSKKMFRLIDFG	390
X. oryzae pv. *oryzicola* BLS256	EYLGMARQLMADVLVGIACCEDAGIVHQDISHNNVMYDQPMKIFRLIDMG	388
X. perforans str. 91-118	EYLGLARQLMADVLVGIACCEDAGIVHQDISHNNVMYDQPMKIFRLIDMG	388
X. vasicola str. NCPPB 1060	EYLALARQLMADVLVGIACCEDAGIVHQDISHNNVMYDQPMKIFRLIDMG	388
ruler	.......360.......370.......380.......390.......400	

```
                                                                      VIII               IX
               X. euvesicatoria str. 85-10 LGAEEGDPNRGGTPGFYDLSTPA--R---HARDVYSVAQLLVHVLKRPDY   433
Acidovorax avenae subsp. avenae ATCC 19860 QGAEPGGPRAQGTAGYMDMHAARAD----HRSDVYAAAQLLVRLLKTPAY   433
            X. alfalfae str. GEV-Rose-07 LGAEEGDPNRGGTPGFYDLSAPA--R---HARDVYSVAQLLVHVLKRPDY   433
             X. axonopodis str. Xac29-1 LGSEEGDPNRGGTPGFYDLSTPA--R---HTRDVYSVAQLLVHVLKRPDY   433
               X. bromi isolate LMG947 LGSEEGDPNRGGTPGFFDVSSPA--R---HTRDVYSVAQLLVHVLKRRDY   433
           X. campestris str. NCPPB4346 LGAEEGDPNRGGTPGFYDLSSPA--R---HARDVYSVAQLLVHVLKRPDY   433
          X. citri subsp. citri str. jx4 LGSEEGDPNRGGTPGFYDLSTPA--R---HTRDVYSVAQLLVHVLKRPDY   433
           X. fragariae isolate Fap21 LGGEEGEPPRAGTPGYIDMSSPA--S---HARDVYSVAQLLVYFLKHPDY   434
     X. fuscans subsp. fuscans str. 4834-R LGAEEGDPNRGGTPGFYDLSTPA--R---HARDVYSVAQLLVHVLKRPDY   433
              X. gardneri str. JS749-3 LGEEEGEPVRYGTPGFIEANPQATDRLASHPRDVYSAAQLLVHFIKCPTH   440
         X. oryzae pv. oryzicola BLS256 LGAEEGDPNRGGTPGFYDLSSPA--R---HARDVYSVAQLLVHVLKRPDY   433
              X. perforans str. 91-118 LGAEEGDPNRGGTPGFYDLSTPA--R---HARDVYSVAQLLVHVLKRPDY   433
          X. vasicola str. NCPPB 1060 LGSEEGDPNRGGTPGFYDLSSPA--R---HARDVYSVAQLLVHVLKRPGY   433
                                ruler .......410.......420.......430.......440.......450

                                                                             X          XI
               X. euvesicatoria str. 85-10 NMGMIGINRAKTADTFPFMDALQA-LPADHKRIVVRFFNSMLDDGTGERT   482
Acidovorax avenae subsp. avenae ATCC 19860 RLGMAGMSGSRTVEDFPFAPALRA-LVPGRLDAAVRFINRMIGRQPDARS   482
            X. alfalfae str. GEV-Rose-07 NMGMIGINRAKTADTFPFMDALQA-LPADHKRIVVRFFNSMLDDGTGERT   482
             X. axonopodis str. Xac29-1 NMGMIGINRPKTADTFPFMDALQA-LPADHKRTVVGFFNSMLDDGTGERT   482
               X. bromi isolate LMG947 NMGMIGINRAKTAETFPFMDPLHA-LPADHKRNVVRFFNRMLNDGTGKRT   482
           X. campestris str. NCPPB4346 NMGMIGINRAKTADTFPFMEALQA-LPADHKRMVVRFFNSMLDDGTGERT   482
          X. citri subsp. citri str. jx4 NMGMIGINRPKTADTFPFMDALQA-LPADHKRTVVGFFNSMLDDGTGERT   482
           X. fragariae isolate Fap21 QMGYIGIFNETSEEKFPFMEALKKNLPFESKREVIRFLNRMISIDANGRA   484
     X. fuscans subsp. fuscans str. 4834-R NMGMIGINRTKTADTFPFMDALQA-LPADHKRTVVHFFNSMLDDGTGERT   482
              X. gardneri str. JS749-3 DMGFTGIFRAKTEEDFPFMDALKQ-LSLKDREEIVRFFNRMITPKENGGA   489
         X. oryzae pv. oryzicola BLS256 NMGMIGINRAKTADTFPFMEALQA-LPADHKRMVVRFFNSMLDDGTGERT   482
              X. perforans str. 91-118 NMGMIGINRAKTADTFPFMDALQA-LPADHKRIVVRFFNSMLDDGTGERT   482
          X. vasicola str. NCPPB 1060 NMGMIGINRAKTADSFPFMDALQA-LPDDHKCTVVRFFNSMLDDGTGQRT   482
                                ruler .......460.......470.......480.......490.......500
```

X. euvesicatoria str. 85-10	KAERLLRDPFFTEPPLPPRDRMHRTYEKL--KRLPSF	517
Acidovorax avenae subsp. *avenae* ATCC 19860	TAEELLGDPFLQE--LEPRALTRATVEKVLNPETPPG	517
X. alfalfae str. GEV-Rose-07	KAERLLRDPFFTEPPLPPRDRMHRTYEKL--KRLPSF	517
X. axonopodis str. Xac29-1	KAERLLRAPFFPEPPLPPRDRMHRTFEKL--TRLPSF	517
X. bromi isolate LMG947	KAEILLRDPFFTETPLPTRDRIHRTYEKL--KRLPSF	517
X. campestris str. NCPPB4346	KAEVLLRDPFFTETPLPPRARMHRTYEKL--KRVPSF	517
X. citri subsp. *citri* str. jx4	KAERLLRAPFFTEPPLPPRDRMHRTFEKL--TRLPSF	517
X. fragariae isolate Fap21	RAEDLLLDSFINDPTIPLRNHVHATYKKL--A-----	514
X. fuscans subsp. *fuscans* str. 4834-R	KAEILLRDPFFTEPPLPPRDRMHRTYEKL--KRLPSF	517
X. gardneri str. JS749-3	TAEDLLKDPFLNAAAIGPRDNVHATLEKLT-------	519
X. oryzae pv. *oryzicola* BLS256	KAEVLLRAPFFTETPLPPRARMHRTYEKL--KRVPSF	517
X. perforans str. 91-118	KAERLLRDPFFTEPPLPPRDRMHRTYEKL--KRLPSF	517
X. vasicola str. NCPPB 1060	KAETLLRNPFFTQQPLPPRDRMHRTYEKL--RRLPSF	517
ruler	.......510.......520.......530.......	
